# Primary sclerosing cholangitis displays distinct colonic mucosa topography yet a shared mast cell state with ulcerative colitis

**DOI:** 10.1038/s41467-026-75231-1

**Published:** 2026-07-07

**Authors:** Jacqueline LE Tearle, Ekaterina Sviriaeva, Fan Zhang, Katherine JL Jackson, Joshua Kaye, Paris Tavakoli, Sabrina Koentgen, Joanna Warren, Raymond R. Liang, Pratibha Malhotra, Cameron Williams, Ashraful Haque, Arteen Arzivian, Kavitha K. Sudhakar, Drew Neavin, Nicodemus Tedla, Andrew Kim, Craig Haifer, Hamish W. King, Georgina L. Hold, Simon Ghaly, Kylie R. James

**Affiliations:** 1 Translational Genomics Program, Garvan Institute of Medical Research, Darlinghurst, Sydney, NSW Australia; 2 School of Biomedical Sciences, University of New South Wales, Sydney, NSW Australia; 3 Microbiome Research Centre, St George and Sutherland Campuses, School of Clinical Medicine, University of New South Wales, Sydney, NSW Australia; 4 Immune Biotherapies Program, Garvan Institute of Medical Research, Darlinghurst, Sydney, NSW Australia; 5 St. Vincent’s Clinical School, University of New South Wales, Sydney, NSW Australia; 6 Cellular Genomics Platform, Garvan Institute of Medical Research, Darlinghurst, Sydney, NSW Australia; 7 Department of Microbiology and Immunology, The Peter Doherty Institute for Infection and Immunity, Parkville, Melbourne, VIC Australia; 8 University of Melbourne, Parkville, Melbourne, VIC Australia; 9 Department of Gastroenterology and Hepatology, St. Vincent’s Hospital Sydney, Darlinghurst, Sydney, NSW Australia; 10 School of Clinical Medicine, Faculty of Medicine and Health, University of New South Wales, Sydney, NSW Australia; 11 Genetics and Gene Regulation Division, Walter and Eliza Hall Institute of Medical Research, Parkville, Melbourne, VIC Australia; 12 Present Address: Frazer Institute, Faculty of Health, Medicine and Behavioural Sciences, The University of Queensland, Brisbane, QLD Australia; 13 Present Address: Institute for Molecular Bioscience, University of Queensland, Brisbane, QLD Australia

**Keywords:** Ulcerative colitis, Mast cells, Transcriptomics, Microbiome

## Abstract

Primary sclerosing cholangitis (PSC) is a chronic, progressing cholestatic disease that often co-occurs with inflammatory bowel disease (PSC-IBD). PSC-IBD affecting the colon (PSC-ulcerative colitis or PSC-UC) resembles clinical UC, but is characterised by less severe disease flares, right-colon predominance, and a greater lifetime risk of colorectal cancer than UC alone. To elucidate differences in the underlying biology between PSC-UC and UC, here we combine single-cell mRNA and antigen receptor sequencing, 16S ribosomal RNA gene analysis and spatial transcriptomics on biopsies from four colon regions of patients with PSC-UC and UC during endoscopic remission or at the time of relapse. We show that the PSC-UC colon, compared to healthy control (HC) or UC colon, harbours distinct and region-specific mucosal-adherent microbial communities and an enrichment of activated CD8 T and γδ T cells at the right colon, even in the absence of histological inflammation. By contrast, a *TMEM176B*^+^ mast cell population that may be pro-tumourigenic is enriched in the colon during disease relapse in both PSC-UC and UC colon. These results highlight that the PSC-UC and UC colonic mucosa are fundamentally different while sharing similar cell programmes during active disease. Our data thus provide insights to guide tailored clinical management and precision therapies.

## Abstract (web summary)

Patients with primary sclerosing cholangitis often manifest colon inflammation similar to ulcerative colitis (PSC-UC), but it is nevertheless pathologically distinct from conventional UC. Here the authors combine single cell sequencing, microbiota analysis and spatial transcriptomics on patient biopsies to dissect the molecular similarities and differences between PSC-UC and UC.

## Introduction

Primary sclerosing cholangitis (PSC) is a rare and chronic cholestatic disease characterised by stricturing of bile ducts, destruction of the biliary tract and fibrosis. Approximately 70% of PSC individuals have concomitant inflammatory bowel disease (PSC-IBD; approximately 2% of IBD cases)^1,2^. 80% of PSC-IBD resembles ulcerative colitis (UC), with colonic involvement (hereafter referred to as PSC-UC), while only 10% of PSC-IBD resembles Crohn’s disease^3^. However, while UC predominantly causes inflammation of the rectum that extends along the sigmoid and descending colon (left colon), PSC-UC is more likely to be pancolitis with a right-sided dominance, and features rectal sparing and backwash ileitis. Furthermore, PSC-UC causes less severe bowel symptoms, but has a greater lifetime risk of progressing to colitis-associated colorectal cancer compared to UC^4^. This has led to the postulation of PSC-IBD as a distinct disease entity from IBD^5,6^.

Studies aimed at understanding PSC-IBD and UC disease mechanisms have highlighted disease-specific intestinal cell signatures^7,8^, microbiota abundances^9^ and risk-associated genetic variants^10^. However, to date, no published study has comprehensively compared these diseases at single-cell resolution or across relevant colonic regions. Furthermore, no study has compared the colonic mucosa in the absence of active inflammation, missing potential baseline differences between these diseases. The cellular and microbial basis for clinical differences between PSC-UC and UC remains poorly defined and is instrumental in guiding disease-specific clinical management.

To comprehensively assess differences and similarities in PSC-UC and UC disease mechanisms, we performed integrated single-cell transcriptomics and bacterial amplicon analysis of inflamed and noninflamed colonic mucosa biopsies from disease-relevant anatomic regions. We reveal a more conserved microbiome in UC than PSC-UC, even in the absence of active inflammation. During histological remission in PSC-UC, we identify unique anatomical patterning of the microbiome and a cytotoxic T cell signature that mirrors the colonic regions most frequently inflamed. During inflammation, cellular programmes converge across diseases, including the emergence of a *TMEM176B+* mast cell state shared between PSC-UC and UC, with evidence for involvement of these cells in immune cell recruitment and platelet activation. These data support unique underlying disease mechanisms of UC and PSC-UC, with common inflammatory programmes at play during disease flare.

## Results

### Patient cohort

**Fig. 1:**
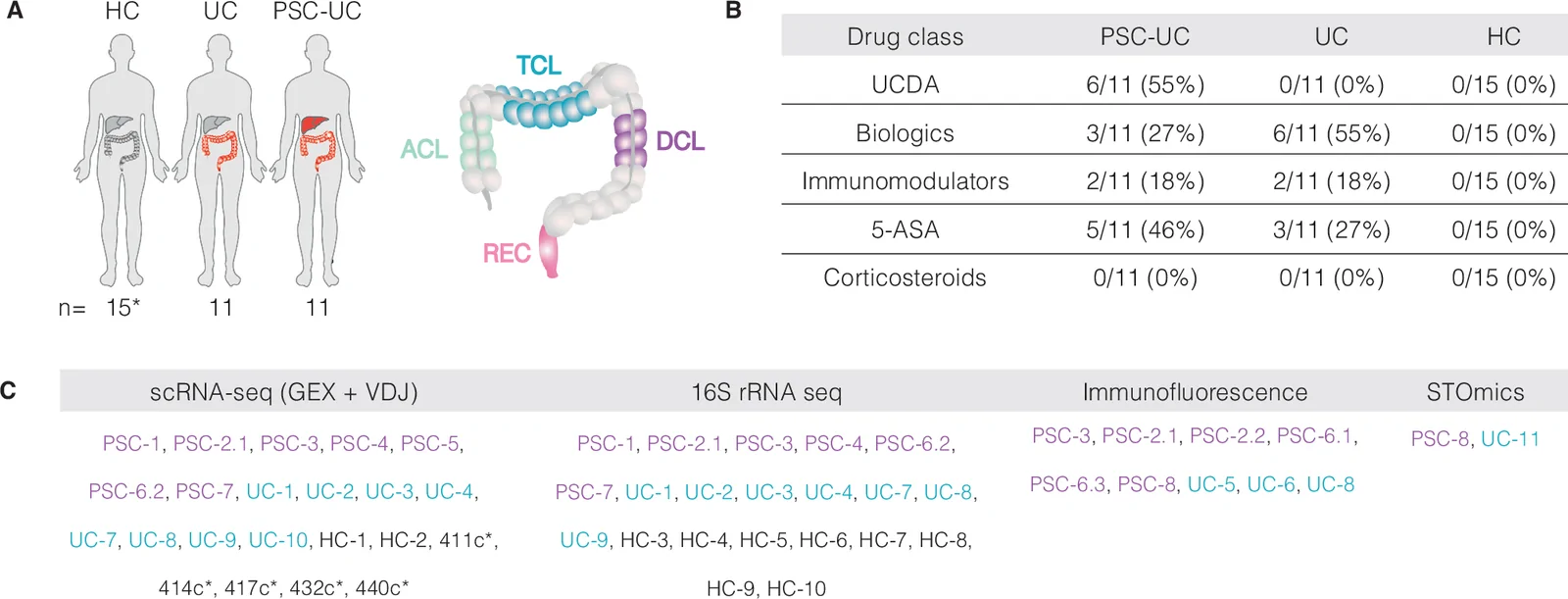
Study overview and patient cohort. **A** Study overview. HC healthy control, UC ulcerative colitis, PSC-UC primary sclerosing cholangitis concomitant with colitis, ACL ascending colon, TCL transverse colon, DCL descending colon, REC rectum. *Published single-cell RNA sequencing data^14^ from four healthy controls was incorporated to contextualise our findings. **B** Therapeutic class use in each patient cohort. **C** Patient IDs used for each analysis.

Biopsies of the mucosa of ascending, transverse and descending colon and rectum were collected from eight PSC-UC and 11 UC patients (Fig. 1A) during routine colonoscopies, and 10 individuals with no reported intestinal pathology (healthy controls; HCs). PSC-2 and PSC-6 were sampled annually over two and three years, respectively. Exclusion criteria included use of antibiotics in the four weeks leading up to colonoscopy and prior liver transplantation. Treatment regimes for each cohort at the time of sampling were roughly comparable between disease groups except for ursodeoxycholic acid (UDCA) used by a subset of PSC-UC participants (Fig. 1B). Further covariates (age, BMI, ethnicity) are summarised in Supplementary Data 1 and full patient metadata, including age at diagnosis, Nancy Index scores at time of sampling, medical history and treatment regimes are provided in Supplementary Data 2. At the time of sampling, three PSC-UC patients and four UC patients experienced active inflammation in one or more colon regions as determined by the gastroenterologist at the time of colonoscopy and histologically confirmed by hospital pathology. Patient cohorts for each assay type are summarised in Fig. 1C and detailed in Supplementary Data 3.

### PSC-UC exhibits distinct colonic microbiome signatures

We previously demonstrated distinct microbial niches throughout the healthy human colon, with a greater abundance of *Enterococcus* at the right colon and *Bacteroides*, *Coprobacillus* and *Escherichia-Shigella* at the sigmoid colon^11^. Moreover, faecal microbiomes^12^ and mucosa-associated microbiomes of the ileum and right colon^13^ have been reported to differ between PSC-IBD and UC. Therefore, we compared the microbial composition of colonic biopsies from a subset of eight HCs, six PSC-UC, and seven UC patients using 16S rRNA gene sequencing

**Fig. 2:**
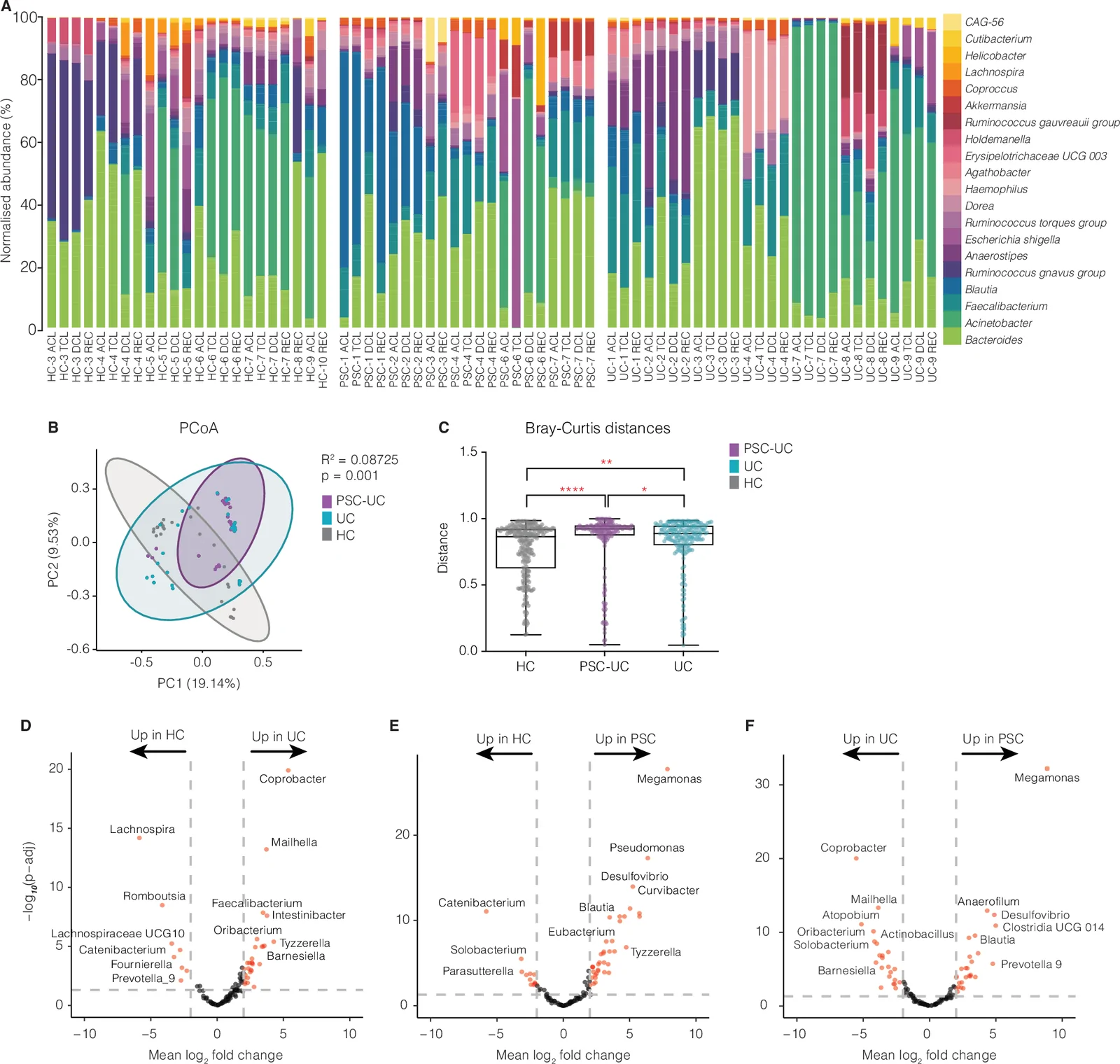
Microbial analyses. **A** Top 20 most abundant genera across all patient samples. **B** Principal coordinate analysis (PCoA) plot coloured by disease state. Each dot represents a single biopsy. **C** Bray-Curtis distance between all biopsy combinations for HC, PSC-UC and UC. Each dot represents a comparison between two biopsies. *N* = 253 for HC, 171 for PSC-UC, 210 for UC. *P* values as determined by two-sided Kruskal-Wallis test = 0.0001 for HC vs. PSC-UC, 0.0038 for HC vs. UC, 0.0176 for PSC-UC vs. UC. Boxes show median and interquartile range, whiskers show minimum and maximum values. **D–F** Volcano plots showing differentially-abundant bacterial genera for UC vs. HC, PSC-UC vs. HC, and PSC-UC vs UC patients. Significant genera are shown in red and indicated by dotted lines at y = 1.3 (*p* > 0.05) and x = 2. Each dot represents values for a single genus.

Overall, we identified 545 bacterial genera across 71 mucosal samples (Fig. 2A, Supplementary Data 4). Principal coordinate analysis revealed that disease was a major source of variability in bacterial genera (Fig. 2B), and overall microbial compositions of each anatomical region were largely conserved within each patient, even during inflammation (Fig. 2A, Supplementary Fig. 1A, B). Bray-Curtis analysis showed that UC patients exhibit significantly reduced interpersonal variation in mucosal microbiome genera compared to PSC-UC or HC (Fig. 2C). We pooled regional samples per patient to compare the overall mucosal adherent microbiomes of HC, PSC-UC and UC (Fig. 2E, F, Supplementary Data 5–7). The UC microbiome was characterised by the consistent enrichment of *Coprobacter*, *Atopobium*, *Oribacterium*, *Mailhella*, *Acinetobacter, Barnesiella, Lachnoanaerobaculum* and *Intestinibacter* compared to PSC-UC and HC. The PSC-UC microbiome was characterised by consistent enrichment of *Pediococcus*, *Agathobacter*, *Paludicola*, *Dialister*, *Bacillus*, *Fusicanibacter*, *Akkermansia*, *Curvibacter*, *Schillibacter*, *Ansertotruncus*, *Pseudomonas*, *Eubacterium*, *Blautia*, *Eisenbergiella*, *Anaerofilum*, *Prevotella 9*, *Desulfovibrio* and *Megamonas* compared to HC and UC. Together, this highlights disease-specific mucosal-adherent microbiomes in remission and during flare, with a more conserved microbiome between UC patients, pointing to fundamentally different microenvironments in PSC-UC and UC.

### Regional microbiome differences in the PSC-UC and UC colon

We next compared mucosal-adherent bacterial communities across anatomical colon regions (Supplementary Fig. 2A–C). This demonstrated pan-colon enrichment of *Akkermansia*, *Megamonas* and *Pseudomonas* in PSC-UC, in line with their enrichment during pooled analyses (Fig. 2E, F). However, most genera displayed regional specificity. For example, *Desulfovibrio, Haemophilus*, and *Oscillibacter*, which are enriched in the right colon (ascending colon and/or transverse colon) of PSC-UC patients compared to both HC and UC patients. Only *Oribacterium* showed consistent anatomical enrichment in the left colon (descending and/or rectum) in UC compared to both HC and PSC-UC (Fig. 2E, F and Supplementary Fig. 2A–C). These results show regional distinction of the microbiome in PSC-UC and UC, with possible relevance to their distinct disease involvements.

### Enriched cytotoxic immune signature in the noninflamed PSC-UC right colon

**Fig. 3:**
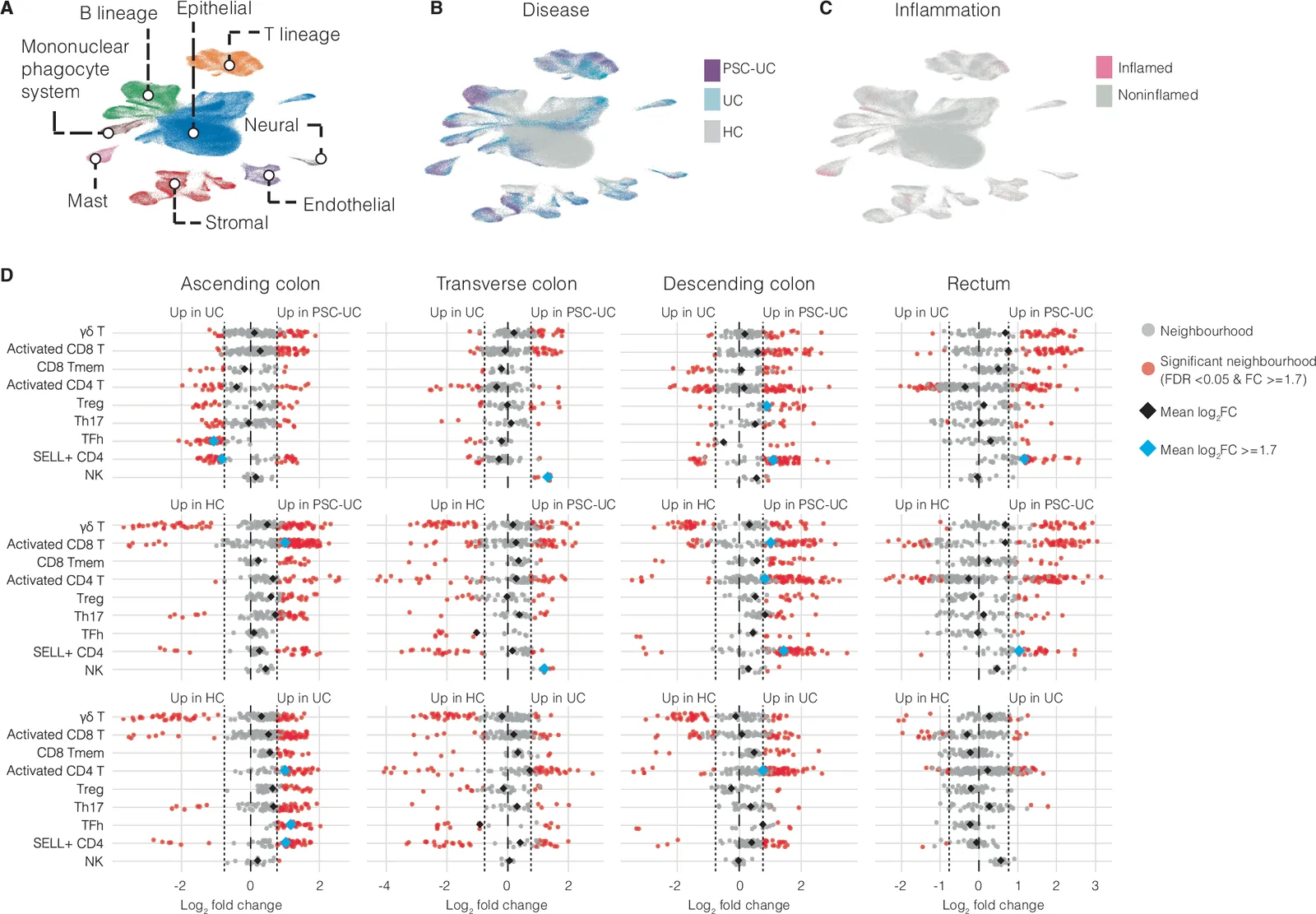
Altered T cell topographies of the UC and PSC-UC gut. **A** UMAP of the scRNA-sequencing dataset coloured by broad cell type, disease (**B**) and inflammation (**C**). **D** Milo analysis showing differential T cell subcluster abundance in the ascending colon, transverse colon, descending colon and rectum. Top shows differential analysis for PSC-UC vs. UC, middle shows PSC-UC vs. HC, bottom shows UC vs. HC. Each dot represents a neighbourhood and significantly altered neighbourhoods are shown in red. Dotted lines represent + or − 1.7.

Having noted regionally distinct microbiome communities in PSC-UC and UC, we wondered whether the co-localising host cell landscapes also show regional specificity in these diseases. To this end, we performed single-cell gene expression analysis on PSC-UC and UC patient biopsies from the same anatomical regions as used for bacterial analysis (*n* = 7 PSC-UC and 8 UC, with 4 colonic regions per patient; Fig. 1A, Fig. 1C and Supplementary Data 3). Additionally, we generated data from two HCs and integrated data from five HC individuals from our published scRNA-seq data^14^. Stringent quality control was followed by Leiden clustering and differential gene expression analysis to define broad cell lineages (Fig. 3A). Cell lineages were serially subclustered for fine-grain annotation of cell subtypes and states (Supplementary Fig. 3 and Supplementary Fig. 4). The final dataset consisted of 320,979 high-quality cells across 58 cell types or states, including 69,510 of PSC-UC origin, 62,968 cells of UC origin (Fig. 3B), 12,262 cells from inflamed regions and 308,717 from noninflamed regions (Fig. 3C). We restricted our analyses to cells from biopsies with no histological inflammation unless otherwise stated.

**Fig. 4:**
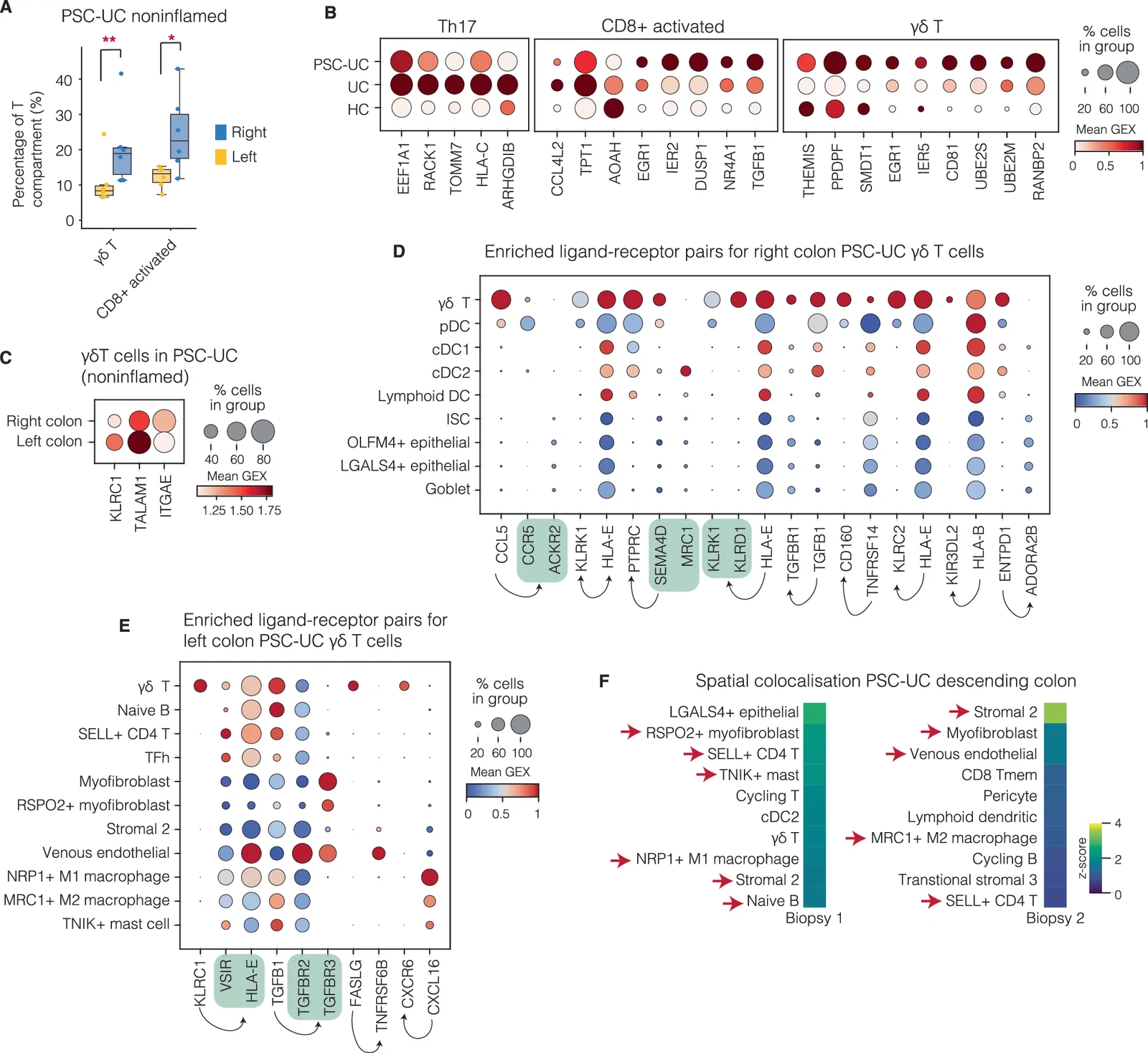
Region and disease-specific T cell gene expression signatures in PSC. **A** Significantly altered relative cell type proportions for PSC-UC right colon (pooled noninflamed ascending and transverse biopsies) vs. left colon (pooled noninflamed descending and rectum biopsies). Each dot represents a single patient pool. *P* values as determined by two-sided paired T tests (paired by patient) = 0.008566 for γδ T cells and 0.036511 for activated CD8 T. Boxes show mean and interquartile range, whiskers show minimum and maximum, excluding outliers. *N* = 6 for left 6 for right. **B** Dot plot showing differentially expressed genes of interest between HC, UC and PSC-UC T cell subtypes. **C** Dot plot showing differentially expressed genes for left colon vs. right colon gdT cells. **D** Dot plot showing gene expression of noninflamed right-sided PSC-UC receptor and ligand transcripts identified as significant by CellphoneDB. **E** Dot plot showing gene expression of noninflamed left-sided PSC-UC receptor and ligand transcripts identified as significantly enriched by CellphoneDB. **F** Top 10 cell types co-localising with left-sided PSC-UC γδ T cells in two biopsies from the descending colon of patient PSC-8.

Within the conventional and unconventional T lymphocyte compartment (Supplementary Fig. 5A) we observed an enrichment of cytotoxic subtypes (activated CD8 T cells and NK cells) in the ascending and transverse colons in PSC-UC (Fig. 3D). On the other hand, UC donors displayed an enrichment of T helper subtypes in the ascending and descending colon (Fig. 3D). Additionally, when comparing right (pooled ascending and transverse) and left (pooled descending and rectum) colon within donors, PSC-UC donors exhibited increased proportions of γδ T cells and activated CD8 T cells in the right colon (Fig. 4A and Supplementary Fig. 5B), which was not seen in HC or UC (Supplementary Fig. 5C, D).

We next performed differential gene expression analyses of T cell subsets between PSC-UC, UC and HCs to determine whether they were transcriptionally as well as regionally distinct (Supplementary Data 8-10). PSC-UC CD8 T cells exhibited reduced expression of immune cell-attracting chemokine *CCL4L2*, anti-apoptotic protein *TPT1*, *AOAH* (recently shown to increase TCR sensitivity^15^), together with increased expression of immediate early genes associated with recent or chronic activation (*EGR1*, *IER2*, *DUSP1*) and tolerance or exhaustion (*NR4A1*, *TGFB1*) compared to HCs (Fig. 4B). PSC-UC γδ T cells similarly displayed an upregulation of genes associated with development and maturation (*THEMIS*), signalling (*PPDPF*), proliferation and cell cycle regulation (*SMDT1*, *UBE2M*, *UBE2S*) and recent or chronic activation (*EGR1*, *IER5*, *CD81*) relative to UC and HCs (Fig. 4B). In contrast we identified downregulation of genes involved in protein synthesis (*EEF1A1*, *RACK1*), mitochondrial function (*TOMM7*), antigen presentation (*HLA-C*) and cell motility (*RACK1*, *ARHGDIB*) in PSC-UC Th17 cells, pointing towards reduced functional activity in PSC-UC Th17 cells compared to UC.

We next asked whether T cell populations also displayed regional transcriptional specialisation in PSC-UC. Significant right versus left differential gene expression was observed only within PSC-UC γδ T cells, where upregulation of epithelial homing transcript *ITGAE* and downregulation of migratory^16^ and proliferatory^17^ transcript *TALAM1* and inhibitory receptor *KLRC1* in the right colon (Fig. 4C) suggests the adoption of a more activated, tissue-resident phenotype in this region.

We next performed ligand-receptor expression analysis to determine whether PSC-UC γδ T cells exhibited regional differences in their predicted cellular interactions. This identified 237 reactions involving γδ T cells that were uniquely significant to the right colon (Supplementary Data 11) and 591 unique to the left (Supplementary Data 12). Notably, γδ T cells in the right colon had a higher proportion of unique interactions with epithelial cells (16% versus 11% in the left) and pDCs (11% versus 0.7% in the left). Expression of *CCL5* by right colon γδ T cells could facilitate recruitment of plasmacytoid DCs to the right colon, and a mix of activating (*KLRK1*, *PTPRC*, *KLRD1/KLRK1* complex *(*CD94/NKG2D*)*, *TGFBR*, *CD160*) and inhibitory (*KLRC2*, *KIR3DL2*) interactions between γδ T cells and DC subtypes could enable DCs to fine tune γδ T cell inflammatory potential (Fig. 4D). Expression of *SEMA4D* and *HLA-E* by epithelial subtypes may influence adhesion and tissue residency of right-sided γδ T cells, and stimulation of epithelial ADORA2B by right-sided γδ T cells could support epithelial barrier integrity during subclinical inflammation^18^ (Fig. 4D). Conversely, T cell interactions unique to the left colon were enriched for T (59%) and stromal (8.5%) lineage cells (Supplementary Data 12). Enrichment of multiple ligand-receptor interactions involving immune checkpoint KLRC1 in the left colon suggests a role for left-sided PSC-UC γδ T cells in regulating local immune responses, specifically via HLA-E and VSIR (Fig. 4E). Binding of FASLG to TNFRSF6B, a decoy receptor, on venous endothelial cells may prevent left-sided endothelial cell death to preserve vasculature, and CXCL16 expression by left-sided myeloid subsets may anchor these anti-inflammatory γδ T cells in the left colon (Fig. 4E). Interaction between left-sided γδ T cells and stromal, myeloid, T cell, and endothelial subsets was further supported by spatial colocalisation data from the descending colon of a noninflamed PSC-UC patient (Fig. 4F).

**Fig. 5:**
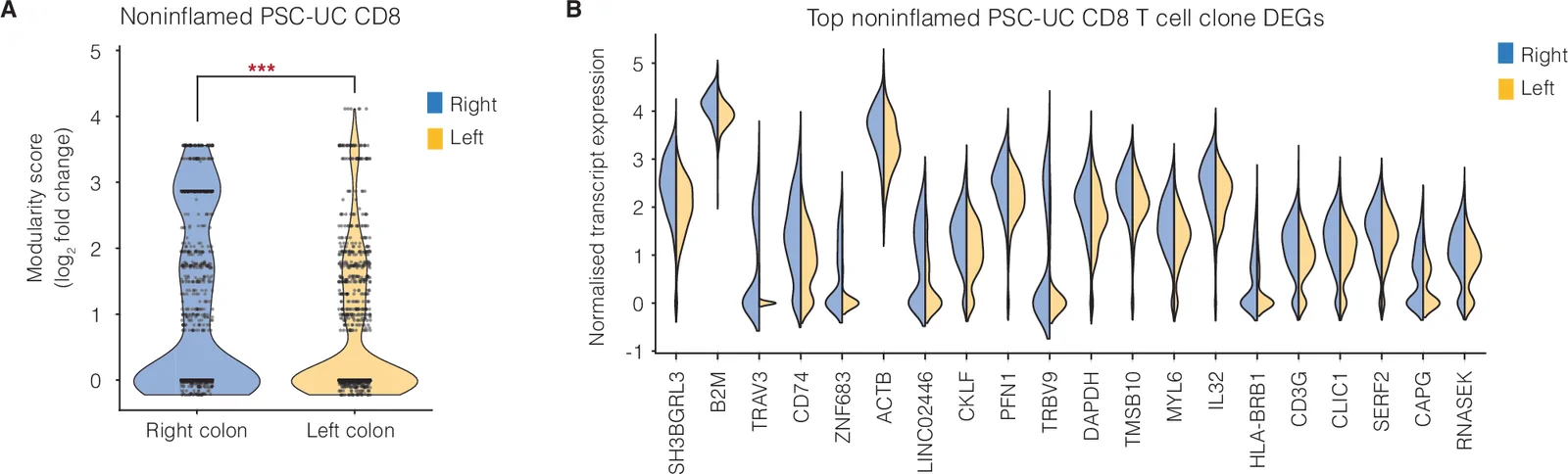
Distinct CD8 T cell state in the remitting PSC-UC right colon. **A** Noninflamed left colon and right colon CD8 T cell modularity scores for PSC-UC. *P* value as determined by two-sided Mann-Whitney U test = 6.252 ×10−^12^. *N* = 1232 for left and 1027 for right. **B** Violin plots for the top 20 differentially expressed genes defined by comparing the top 30 (5 per patient) most highly expanded noninflamed right colon CD8 T cell clones vs. the top 30 (5 per patient) most highly expanded noninflamed left colon CD8 T cell clones.

Although regional gene expression differences were not prominent within ɑβ T cells, we considered that disease-relevant variation may instead be encoded via clonal architecture. We therefore analysed paired ɑβ T cell receptor sequences, which revealed that PSC-UC patients in remission exhibited greater CD8 T cell clonal expansion in the right colon compared to UC patients (Supplementary Fig. 6A). However, when normalising clonotype size to the overall number of CD8 T cells, no difference was detected (Supplementary Fig. 6B). We next examined repertoire overlap as a measure of clonal sharing across regions. UC patients displayed the lowest degree of clonal repertoire overlap for both CD4 and CD8 T cells, indicating greater compartmentalisation of T cell clones between colonic regions. On the other hand, HCs displayed the highest degree of overlap, consistent with greater circulation of shared clones throughout the colon (Supplementary Fig. 6C–F). To determine whether clonally-related cells occupied similar transcriptional states, we assessed T cell receptor modularity, a measure of transcriptional similarity within individual clones. This revealed higher CD8 T cell modularity in PSC-UC than in UC (Supplementary Fig. 7A). Furthermore, for PSC-UC, the modularity of CD8 T cells (but not CD4 T cells) was significantly higher in the right colon compared to the left (Fig. 5A, Supplementary Fig. 7B), suggesting the unique right-sided PSC-UC microenvironment drives clonally-related CD8 T cells into a highly synchronised state. Interestingly, UC patients displayed an inverse pattern of modularity, with no difference between left and right colon CD8 T cells, and increased modularity in left colon CD4 T cells (Supplementary Fig. 7B-D). Finally, to investigate the functional characteristics of these right-sided PSC-UC CD8 T cell clones, we compared gene expression profiles of the five largest clones from the right and left colon of each patient. Right-sided clones upregulated genes associated with tissue residency (*ZNF683*), cytotoxic activation (*B2M*, *CD74*) and inflammation (*IL32*), further supporting the notion of a distinct, inflammatory CD8 T cell state in the remitting PSC-UC right colon (Fig. 5B). Analysis of B cell receptor sequences revealed an increase in IgG1 usage for both disease cohorts, as expected, but no difference in the clonal diversity (Shannon equitability) compared with HCs for either colon side (Supplementary Fig. 8A–C).

Collectively, these findings indicate that the PSC-UC right colon harbours a specialised cytotoxic immune niche characterised by enriched tissue-resident γδ T cells and transcriptionally synchronised CD8 T-cell clones. The presence of this niche in histologically noninflamed tissue suggests ongoing subclinical disease and aligns with the characteristic right-sided disease distribution of PSC-UC.

### Distinct non-immune cellular and transcriptional signatures of the remitting PSC-UC colonic mucosa

**Fig. 6:**
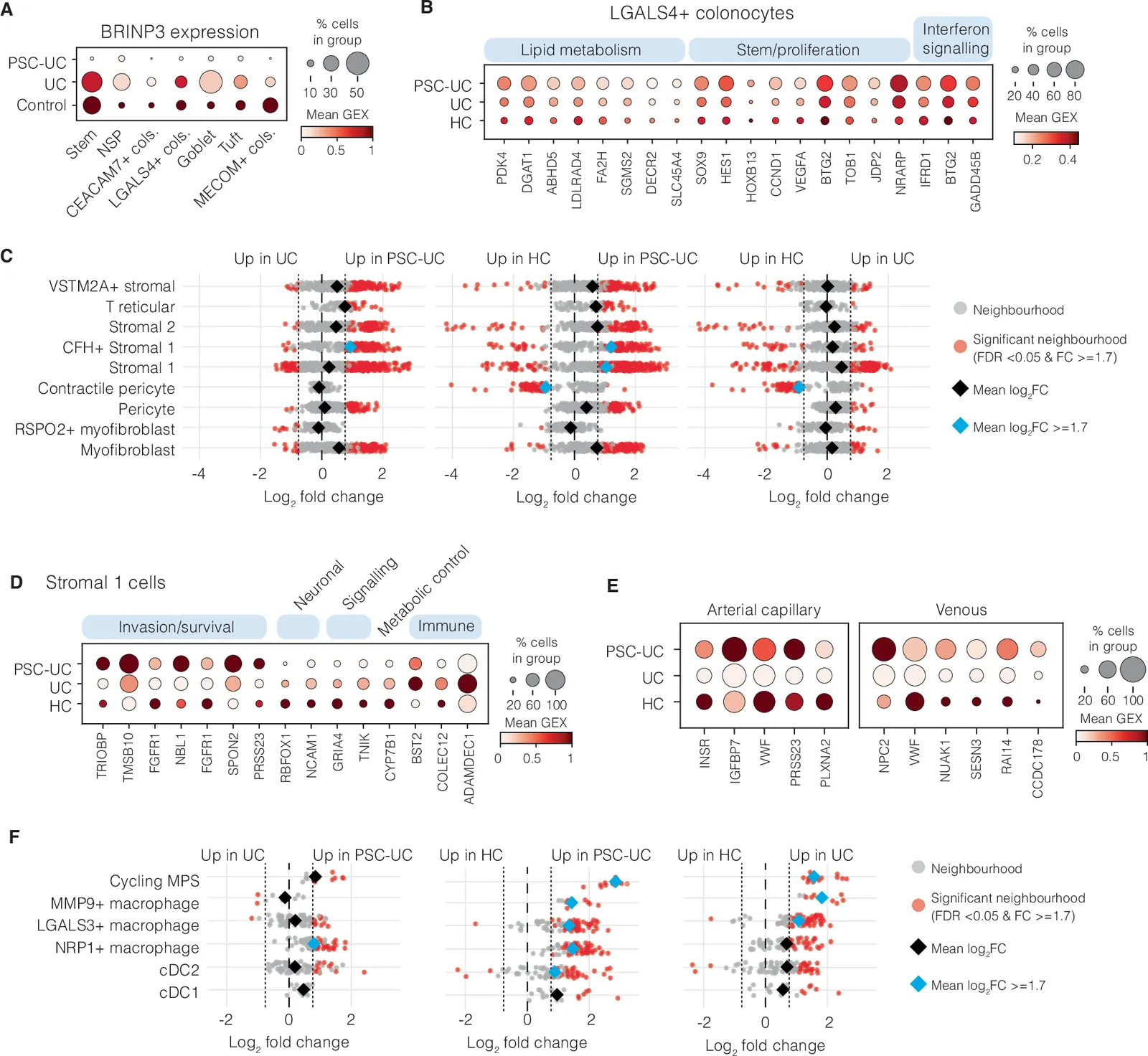
Epithelial, stromal, vascular and mononuclear phagocyte system cells of the UC and PSC-UC gut. **A** Dot plot showing mean expression of *BRINP3* in remitting UC, PSC-UC and HC epithelial cell subtypes. **B** Dot plot displaying gene expression for key upregulated genes in PSC-UC *LGALS4*+ epithelial cells. **C** Milo analysis showing differential stromal cell subcluster abundance for UC vs. PSC-UC, PSC-UC vs. HC and UC vs. HC. Each dot represents a neighbourhood and significantly altered neighbourhoods are shown in red. **D** Dot plot showing key differentially expressed genes in stromal 1^14^ cells. **E** Dot plot showing expression of transcripts of interest in noninflamed PSC-UC, UC and HC arterial capillary and venous cells. **F** Milo analysis showing differential mononuclear phagocyte system subcluster abundance for UC vs. PSC-UC, PSC-UC vs. HC and UC vs. HC. Each dot represents a neighbourhood and significantly altered neighbourhoods are shown in red.

Given the growing appreciation for the role of epithelial, stromal and endothelial cell types in intestinal dysbiosis^19^, we next compared, for the first time at single-cell resolution, these cellular compartments in the PSC-UC and UC gut. To assess differences in underlying disease biology, we determined relative cell type proportions of biopsies taken from regions with no histological evidence of inflammation. Within the epithelial compartment (Supplementary Fig. 9A), this revealed an enrichment of tuft cells in UC compared to PSC-UC and HC, and an enrichment of almost all major epithelial subtypes in HCs (Supplementary Fig. 9B). To examine potential differences in epithelial cell function between cohorts, we next compared gene expression for epithelial cells between remitting UC, PSC-UC and HC donors (Supplementary Data 13-15). While downregulation of *BRINP3* has previously been reported in the IBD colon compared to HCs^20^, we report a further and striking downregulation of *BRINP3* in PSC-UC samples compared to UC and HCs across all colonocyte populations, including tuft and goblet cells (Fig. 6A). We also identify upregulation of genes involved in lipid transport and metabolism (*PDK4*, *DGAT1*, *ABHD5*, *LDLRAD4*, *FA2H*, *SGMS2*, *DGAT1*, *DECR2*, *SLC45A4*), stemness and proliferation (*SOX9*, *HES1*, *HOXB13*, *CCND1*, *VEGFA*, *BTG2*, *TOB1*, *JDP2*, *NRARP*), and interferon signalling (*IFRD1*, *BTG2*, *GADD45B*) in PSC-UC *LGALS4*+ colonocytes (the most prevalent epithelial subtype) compared to UC and HC (Fig. 6B). Overall, suggesting that PSC-UC associated colonocytes exhibit a distinct phenotype characterised by enhanced bioenergetic supply, capacity for self-renewal, and inflammatory response.

Within the stromal compartment (Supplementary Fig. 10A, B), PSC-UC patients exhibited an increased proportion of *CFH*+ stromal 1^14^ cells compared to both UC and HCs, and an increase in stromal 1 cells compared to HCs (Fig. 6C). Both PSC-UC and UC patient samples displayed a reduction in contractile pericytes compared to HCs. To explore potential differences in cell state and function, we next assessed differences in stromal cell gene expression between cohorts (Supplementary Data 16–18). While PSC-UC and UC patients displayed equitable proportions of stromal 1 cells, PSC-UC patient stromal 1 cells exhibited an enrichment of genes related to invasion, survival and remodelling (*TRIOBP*, *TMSB10*, *FGFR1*, *NBL1*, *FGFR1*, *SPON2*, *PRSS23*) along with reduced expression of genes related to neuronal-like differentiation, (*RBFOX1*, *NCAM1*), complex signalling (*GRIA4*, *TNIK*), metabolic control (*CYP7B1*), and immune surveillance (*BST2*, *COLEC12*, *ADAMDEC1*, Fig. 6D), overall implying a shift towards a more simplified, pathologic or de-differentiated state.

Within the vascular compartment (Supplementary Fig. 3, Supplementary Fig. 10C, D), we observed no difference in cell type proportions between PSC-UC and UC cohorts (Supplementary Fig. 9D), however HC samples displayed enrichment of mature venous and arterial cells when compared to both PSC-UC and UC. Comparison of gene expression between disease cohorts identified an upregulation in genes related to insulin signalling (*INSR*, *IGFBP7*) and vascular activation and remodelling (*VWF*, *PRSS23*, *PLXNA2*) in arterial capillary cells, and an upregulation of genes related to cholesterol metabolism (*NPC2*), stress response and proliferation (*NUAK1*, *VWF*, *SESN3*, *RAI14*, *CCDC178*) in venous cells in PSC-UC (Fig. 6E, Supplementary Data 19–21). These gene enrichments could imply that the PSC-UC endothelial compartment is under metabolic and oxidative stress, and may be attempting repair and proliferation in response to injury.

Previous works have implicated macrophage function in PSC pathogenesis^21,22^. To explore the cellular diversity and function of the mononuclear phagocyte system (MPS) in the PSC-UC gut, we performed additional subclustering of the MPS compartment (Supplementary Fig. 3 and Supplementary Fig. 10E, F). Comparison of cell type proportions in noninflamed samples (Fig. 6F) revealed that both UC and PSC-UC patients displayed an enrichment of cycling, *MMP9*+ and *LGALS3*+ macrophages compared to HCs. However, PSC-UC patients also displayed an enrichment of *NRP1*+ macrophages compared to UC and HCs. *NRP1*+ macrophages were characterised by elevated expression of *SASH1*, pro-tumourigenic macrophage transcript *NRP1*^23^, pro-inflammatory M1 markers *FOLR2* and *CD163L1*^24^ as well as *OTULINL* and *KLF2* (Supplementary Fig. 3), matching a population recently reported in the PSC liver^22^, highlighting potential convergence between liver and gut pathologies.

### Shared TMEM176B+ mast cell signature in inflamed PSC-UC and UC colon

**Fig. 7:**
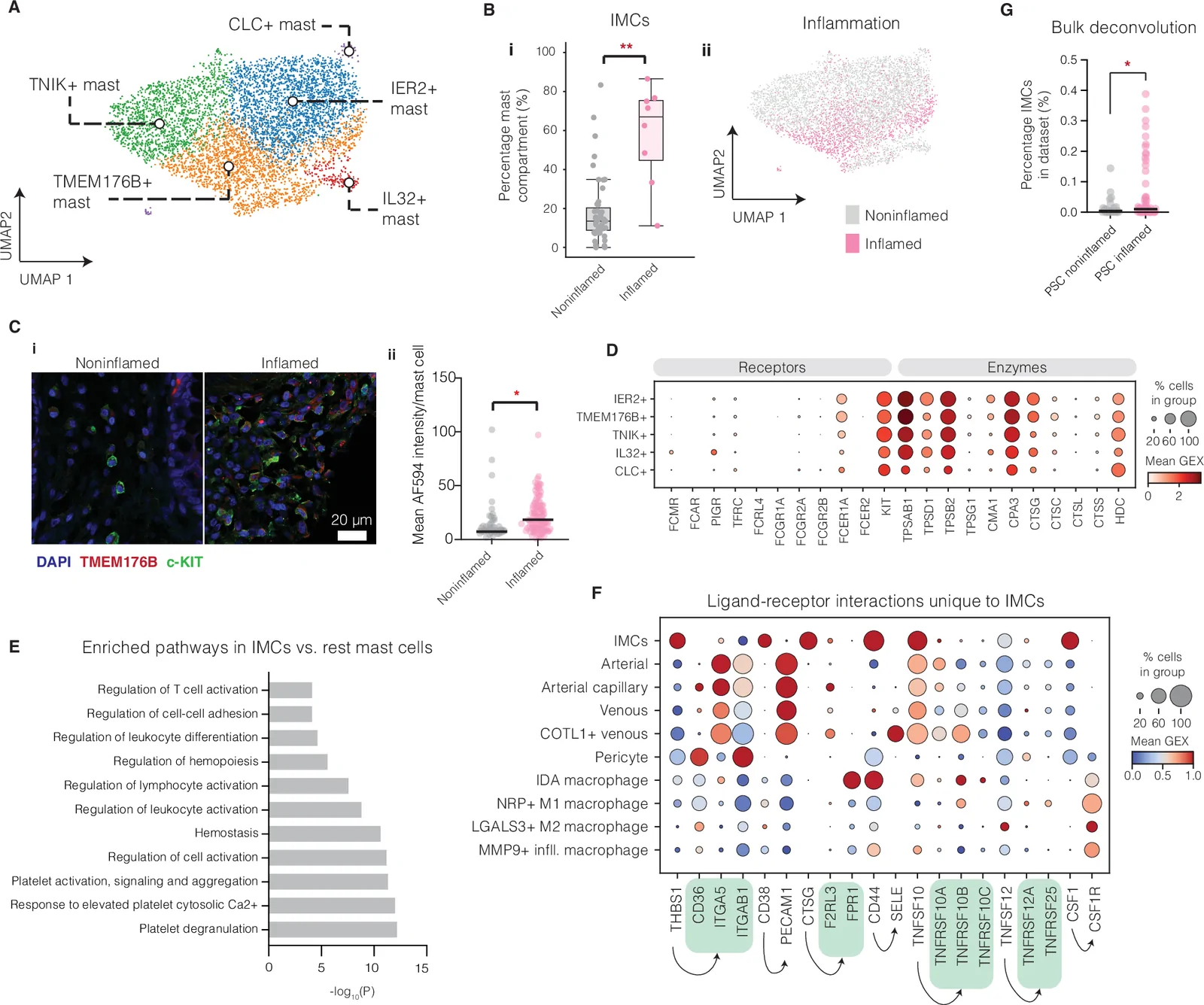
UC and PSC-UC inflammation is characterised by infiltration of *TMEM176B+* mast cells. **A** UMAP of the mast cell compartment, coloured by mast cell subtype. **B** i. Percentage of inflammatory mast cells (IMCs) in the mast cell compartment of the scRNA-sequencing dataset for noninflamed and inflamed samples. Each dot represents one biopsy. *N* = 47 for noninflamed and 8 for inflamed. *P* value as determined by two-sided Mann-Whitney U test = 0.002806. Boxes show mean and interquartile range, whiskers show minimum and maximum, excluding outliers. Biopsies containing <5 mast cells (mainly HCs) were excluded from analysis. ii. UMAP of the mast cell compartment, coloured by inflammation. **C** i. Representative immunofluorescence images of noninflamed and inflamed PSC-UC tissue. A total of four inflamed biopsies and five noninflamed biopsies were tested as shown in Supplementary Fig. 11. ii. Mean AF594 fluorescence intensity (TMEM176B staining) per mast cell defined by DAPI and c-KIT using Imaris software. Each dot represents one cell within the larger representative immunofluorescence image. *P* value as determined by two-sided unpaired T test = 0.017. *N* = 45 for noninflamed and 107 for inflamed. Line shows mean. **D** Dot plot showing transcript expression for common mast cell receptors and enzymes in each mast cell subtype. **E** Enriched pathway terms in IMCs based on the top 100 upregulated genes in IMCs vs. other mast cells. **F** Dot plot showing gene expression for possible ligand-receptor pair transcripts identified by CellphoneDB for IMCs and other cell types. **G** Deconvolution of Shaw et al.’s^8^ PSC-IBD bulk RNA sequencing dataset using our own scRNA-seq data identified an enrichment of the IMC signature in PSC-IBD patients with intestinal inflammation. *P* value as determined by two-sided unpaired T test = 0.0028. *N* = 27 for noninflamed, 57 for inflamed. Each dot represents one bulk tissue sample.

After deeply contrasting cells of the noninflamed PSC-UC and UC intestinal environments, we next explored how they compared in the context of active inflammation. Leiden clustering of our entire single cell gene expression dataset showed clear separation by tissue inflammation status for plasma cells (separating predominately by IGHG1 isotype expression) and mast cells (Fig. 3C). Subclustering of the mast cell compartment revealed five subtypes delineated by the expression of *TNIK*, *CLC*, *IER2*, *IL32* and intracellular cation channel *TMEM176B* (Supplementary Fig. 3 and Fig. 7A). *TMEM176B*+ mast cells (hereafter termed inflammation-associated mast cells; IMCs) were notably enriched during inflammation in both PSC-UC and UC (Fig. 7B, C and Supplementary Fig. 11).

Both IMCs and other detected mast cell subtypes expressed tryptases (*TPSAB1* and *TPSB2*), carboxypeptidase (*CPA3*) and low levels of chymase (*CMA1*) (Fig. 7D) in contrast to the classical mucosal signature of tryptase alone^25,26^. Notably, IMCs displayed significantly decreased expression of the relatively unstudied delta tryptase enzyme (*TPSD1*; Fig. 7D). Furthermore, all subsets (except *IL32*+ mast cells) expressed the FcERI receptor but no other immunoglobulin-activated receptors (Fig. 7D). We did not detect associated IgE transcript expression in plasma cells of inflamed patients, although we detected small amounts in cycling and memory B cells (Supplementary Fig. 12A). However, in the single cell V(D)J sequencing data, no B cells or plasma cells were observed to express IgE immunoglobulin heavy chain transcripts. Interestingly, pathway analysis of the top 100 enriched genes in IMCs compared to other mast cells identified enrichment of gene programmes involved in platelet degranulation, haemostasis and hemopoiesis, and leucocyte differentiation and activation (Fig. 7E and Supplementary Data 22). Additionally, ligand-receptor analyses for mast cells in our inflamed dataset identified many interactions with endothelial cells that were unique to the *TMEM176B*+ subset (Fig. 7F, Supplementary Data 23), including via IMC-enriched transcript thrombospondin 1 (*THBS1*), known to drive platelet activation^27^, regulate angiogenesis^28^, activate latent TGFβ, and recruit immune cells^29^. Predicted interactions involving *CD38* and *CD44* suggest enhanced communication with endothelial *PECAM1*- and *SELE*-expressing cells, pointing to the localisation of IMCs to the vasculature. Furthermore, expression of *THBS1*, *CSF1*, *TNFSF10* and *ANXA1* by IMCs was predicted to mediate interactions with macrophages, potentially promoting their recruitment and polarisation^29,30^.

Compared to other mast cells, the IMC gene signature was characterised by expression of many transcripts associated with FcERI-mediated activation, such as cytokine-dependent hematopoietic cell linker (*CLNK*)^31^, hematopoietic cell signal transducer (*HCST*, encoding the DAP10 protein) and *PDE3B*^32^ (Supplementary Data 24 and Supplementary Fig. 12B). However, for each of these transcripts, genes encoding known interacting partners and downstream mediators (*PLCG1*, *GRB1*, *LAT* for *CLNK*^31^ and *KLRK1*, *SIRPB1* for *HCST*^33–35^) were not enriched in IMCs, pointing either toward a yet uncharacterised pathway occurring during intestinal inflammation, or discrepancies between mRNA expression and protein levels. Notably, *HCST* expression has been described in tumour-infiltrating mast cells in renal cell carcinoma^36^. The IMC signature was also observed in an independent inflamed UC single-cell RNA-seq study^24^ (Supplementary Fig. 13A, B) and enriched in deconvoluted UC and PSC-UC vs. healthy bulk RNA-seq data^7^ (Supplementary Fig. 13C) and inflamed versus noninflamed PSC-IBD colonic bulk RNA-seq data (Fig. 7G)^8^.

**Fig. 8:**
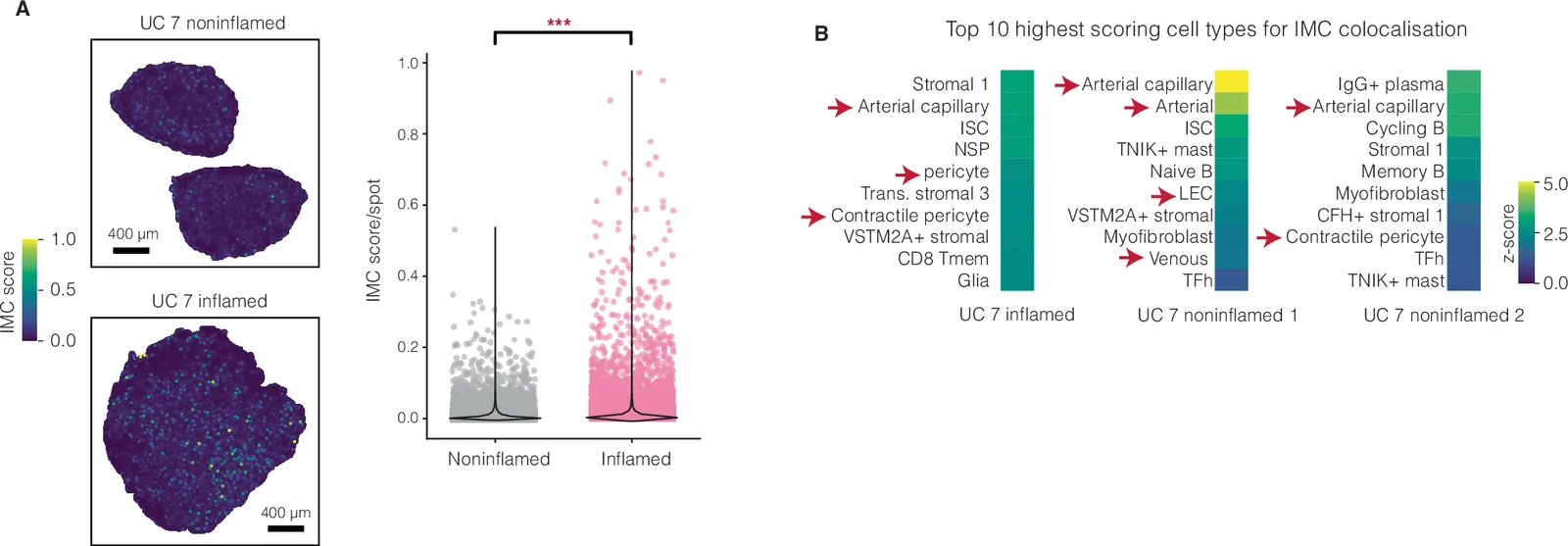
Spatial transcriptomics. **A** Spatial transcriptomics sequencing of inflamed and noninflamed UC tissue deconvoluted using our scRNA-seq dataset and coloured by IMC score (left) and quantified (right). *P* value as determined by two-sided Mann-Whitney U test = 3.93e^−30^. *N* = 39469 for inflamed and 25310 for noninflamed. Each dot represents one spatial spot from images shown to the left. **B** Co-localisation scores generated from the inflamed UC spatial transcriptomics dataset for inflammatory mast cells (IMCs). Top 20 highest scoring cell types co-localising with IMCs.

Spatial transcriptomic analysis of biopsies from inflamed and neighbouring noninflamed regions of an additional UC patient further supported the enrichment of IMCs in inflammation (Fig. 8A). Furthermore, IMCs most commonly colocalised with endothelial cell types in all regions (Fig. 8B), fitting with their enriched GO terms (Fig. 7E), ligand-receptor analyses (Fig. 7F) and reported positioning near vessels^26^. Overall, these analyses suggest that IMCs adopt a specialised phenotype geared towards local immune recruitment and vascular remodelling in the inflamed colon.

### TMEM176B expression negatively regulates mast cell degranulation and activation

**Fig. 9:**
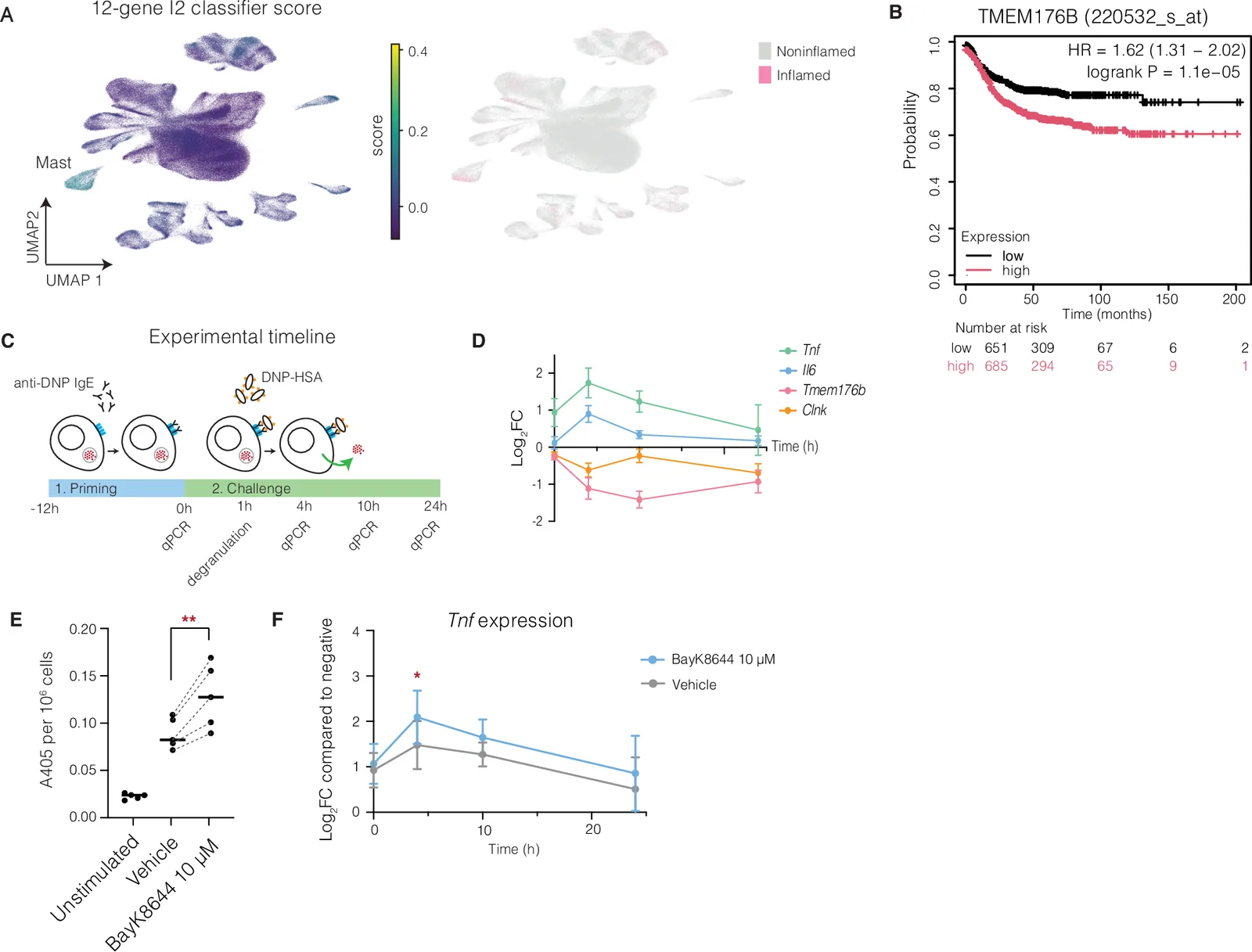
TMEM176B as a negative regulator of classical mast cell activation. **A** UMAPs coloured by expression of Shaw et al.’s 12-gene I2 classifier gene list predictive of colorectal cancer in PSC-IBD (left) and inflammation (right). **B** Kaplan-Meier plot for TMEM176B expression in colorectal cancer sourced from kmplot.com. **C** Experimental design to assess gene expression by qPCR and degranulation by quantification of β-hexosaminidase in mast cell supernatant. **D** Time course expression of *Tnf*, *Tmem176b*, *Clnk* and *Il6* in murine mast cells following activation via FCERI stimulation. Each dot represents the mean of six biological repeats (*n* = 6), each consisting of at least two technical replicates. Bars show SEM. **E** Assessment of mast cell degranulation following FCERI-mediated activation in the presence of vehicle (DMSO) and TMEM176B inhibitor BayK8644. Each dot represents a separate biological replicate (*n* = 5) and is the mean of at least two technical replicates. *P* value as determined by two-tailed paired T test = 0.0091. **F** Time course expression of *Tnf* in murine mast cells following activation via FCERI stimulation in the presence of vehicle (DMSO) and 10 µm BayK8644. Each dot represents the mean of five biological repeats, each consisting of three technical replicates. Error bars represent SEM for the five biological repeats at each timepoint. *P* value as determined by two-tailed paired T test = 0.0150.

A recent study comparing the inflamed PSC-UC and UC colonic mucosa defined a gene signature predictive of dysplasia in PSC-UC^8^. We therefore took advantage of our single cell data to determine the cell type origin of this inflammatory signature. By scoring each cell type by the combined expression of the published 12-gene signature, we revealed mast cells as a major expressor (Fig. 9A). Furthermore, mast cells expressing *TMEM176B* (as well as IMC-specific transcript *CD52*) are enriched in colorectal cancer^37^, and *TMEM176B* expression correlates with decreased survival in this patient group (Fig. 9B), together supporting the notion that *TMEM176B*+ mast cells may be pro-tumourigenic.

We therefore explored the function of *TMEM176B* expression in mast cells to understand the significance of IMCs in colitis. We performed RT-qPCR to quantify expression of activation (*Tnf, Il6*) and IMC (*Tmem176b*, *Clnk*) markers in the Cl.MC/C57.1 mast cell line^38^ following FcERI-mediated activation (Fig. 9C). This revealed a reduction in both *Tmem176b* and *Clnk* following stimulation with cognate antigen compared to negative controls (Fig. 9D), suggesting that these transcripts may be negative regulators of classical FcERI-mediated mast cell activation. We next assessed the role of TMEM176B in mast cell activation via the use of BayK8644, an L-type voltage-dependent calcium channel agonist shown to inhibit TMEM176B activity^39^. Inhibition of TMEM176B resulted in increased degranulation (Fig. 9E) and increased expression of *Tnf* at 4 hours following FcERI-mediated activation (Fig. 9F). Together, this suggests that TMEM176B functions as a negative regulator of classical IgE-mediated mast cell activation. When considered alongside the transcriptional profile of IMCs, these data raise the possibility that attenuation of canonical mast cell activation is accompanied by the acquisition of alternative effector functions, such as *THBS1*-dependent platelet activation, with potential pro-tumourigenic consequences^40,41^.

## Discussion

A challenge in developing targeted therapeutics for PSC-UC is the limited understanding of how its pathogenesis compares to that of IBD. Previous studies have shown differences at cellular and microbial levels between PSC-IBD and UC, but with limited appreciation of the contribution of diverse cell lineages to intestinal immunity^19^ and consideration of distinct anatomical locations. Our pioneering paired single-cell and microbial assessment of PSC-UC and UC, covering the right and left colon and including active and remittent disease, reveals that the intestinal cellular landscape of these patient cohorts is distinct, even in the absence of active disease, and displays anatomical patterning that mirrors the colon regions most commonly involved.

We demonstrate notable differences in microbial composition between UC, PSC-UC and HC cohorts, which contrast with previous studies reporting no difference in mucosal microbial diversity between UC and PSC-IBD at different colon locations^13,42,43^. These discrepancies may be due to differences in inflammatory status of biopsies across the studies. In line with previous studies^44–46^, we report an enrichment of *Acinetobacter* in UC, many species of which are associated with healthcare-related infections and antibiotic resistance. We also report an enrichment of oral genera *Oribacterium* and *Atopobium* in UC, adding to the growing body of evidence supporting the role of oral microbial dysbiosis and translocation in IBD^47,48^. Previous studies have similarly reported enrichment in *Pseudomonas*^7^ and *Blautia*^42^ in the PSC-UC mucosal microbiome, as well as *Desulfovibrio* in patients with chronic liver disease^49,50^. *Desulfovibrio*, a sulphate-reducing bacterium, is a major generator of cytotoxic hydrogen sulphide and has been shown to induce expression of pro-inflammatory cytokines IL6 and IL8 in epithelial cells^51^, though whether these effects are evident in the PSC-UC intestinal mucosa is yet to be determined. Limited overlap in bacterial populations between our study and previous works may be due to the effect of geographical location- no previous studies have characterised the Australian PSC-UC mucosal microbiome. Our observation of a more conserved microbiome in UC compared to PSC-UC suggests that in PSC-UC, microbiome composition may play a less dominant role in intestinal dysbiosis than in UC. Nevertheless, further work is needed to decipher whether the microbial alterations described in PSC-UC are an initiating factor for colitis in PSC patients, or concomitant with cholestatic liver disease in general.

We revealed a distinct cell signature in the right colon of PSC-UC patients marked by increased proportions of NK and γδ T cells and expansion of pro-inflammatory CD8 T cell clones, pointing towards an active right-colon immune response even during symptomatic remission. This low-level, subclinical inflammation presents a challenge for current standard clinical care, which relies primarily on histology or visible endoscopic mucosal characteristics, particularly given the links between inflammation extent and colitis-associated colorectal cancer occurrence^52^. The adoption of specific diagnostic biomarkers capable of detecting this subclinical state in PSC-UC could enable proactive, targeted therapeutic intervention, potentially reducing colorectal cancer incidence in this high-risk population. Previous works have identified expanded γδ T cells in the peripheral blood^53^ and liver^54^ of PSC patients, pointing toward shared cellular mechanisms between liver and gut disease manifestations. From these findings, it is tempting to speculate that a yet unknown autoantigen initiates inflammation of the biliary tree and disrupts bile acid secretion, which results in an altered microbiome^55^. This in turn drives a distinct local immune cell response which contributes to the right-sided dominance^56^ of colon inflammation and perpetuates liver fibrosis.

We identified a conserved *TMEM176B+* mast cell subtype (IMCs) in our and other published IBD datasets. IMCs were characterised by a non-classical and CRC-associated^37^ transcriptomic signature, gene set enrichment for platelet degranulation and vascular repair processes, and spatial colocalisation with endothelial and intestinal crypt cells, suggesting a role in local haemostasis and tissue remodelling. We propose that TMEM176B is a negative regulator of classical mast cell activation, which could in turn support non-canonical and pro-tumourigenic functions suggested from our expression analyses. While we did not detect notable levels of IGHE in our dataset, IgE-expressing B and plasma cells are rare and largely short-lived^57,58^, and IgE generated outside the gut may have initiated an IMC response. While previous works have demonstrated elevated IgE in blood of PSC^59–61^ and IBD^62,63^ patients, further work is needed to determine the specific drivers of the IMC state. The enrichment of IMCs during inflammation and colorectal cancer^37^, and a likely contribution of mast cells to a pro-dysplasia inflammatory signature in PSC-IBD, provide compelling evidence for the involvement of mast cells in colon tumourigenesis, and may constitute a valuable therapeutic or predictive target.

Analysis of patient samples in this study presents notable limitations. Firstly, it was infeasible to limit this prospective study to treatment-naive IBD patients, and their diverse therapeutic histories likely impact their cellular responses and microbiome compositions. Biologic, immunomodulator and 5-ASA use at the time of sampling are broadly comparable between disease cohorts in our study. Therefore, these specific therapeutic differences are unlikely to be the primary drivers of the differences in immune and microbial compositions reported between PSC-UC and UC. To further limit the effects of diverse therapeutic histories and strengthen the likelihood that reported signatures reflect fundamental, underlying disease biology, we performed inpatient comparisons of right vs. left when analysing anatomical differences between diseases and prioritised results consistent across all participants in a cohort. The primary exception to therapeutic comparability is UDCA, which was exclusively used by a subset of PSC-UC patients. UDCA, a therapeutic bile acid commonly used to treat chronic cholestatic liver diseases, has previously been demonstrated to alter the gut microbiome composition^64^, and therefore presents a potential confounder for the observed PSC-UC microbiome signature. Routine bowel preparation before colonoscopy is also likely to reduce microbial diversity of patient samples. While PSC is more often diagnosed before the onset of colitis^6^, it is possible that UC patients included in this study go on to develop PSC. Such cases would shed more light on whether the distinct right colon cell profile of PSC-UC is the result of or contributing to liver disease. We used the best available murine mast cell line that expresses high FcεRI and key markers, such as KIT, CD33, and CD203c^65^ as isolating and culturing patient-derived primary mast cells was infeasible, and none of the few human mast cell lines available to date^66^ was suitable for this study. Future work may look to animal models to further study the role of IMCs in intestinal inflammation, though these present their own challenges in their relatability to human intestinal disease.

In summary, we have generated the first unbiased parallel single-cell mRNA and bacterial sequencing dataset of the PSC-UC and UC large intestine. In doing so, we have demonstrated that the PSC-UC and UC colons are microbially and cellularly distinct even in the absence of active disease. In particular, PSC-UC patients display distinct anatomical patterning not seen in UC, with unique immune cell enrichments in the right colon. Finally, we identify a shared *TMEM176B+* mast cell response during inflammation and provide evidence for the involvement of these cells in intestinal tumourigenesis. The disease-specific cell and microbial signatures identified from this work represent tools for patient stratification and avenues for precision therapies.

## Methods

### Sample collection and patient cohort

Use of human material in this study was conducted in accordance with relevant ethical regulations and approved by the South Eastern Sydney Local Health District Research Ethics Committee (Australian IBD Microbiome (AIM) study^67^; HREC ref no. 18/173) and the St Vincent’s Hospital Human Research Ethics Committee (2024/ETH01125). All participants provided informed written consent before enrolment. Mucosal pinch biopsies were collected from the ascending colon, transverse colon, descending colon and rectum of patients with either UC or PSC-UC (classified as resembling UC at the clinical level) during routine colonoscopy procedures. Mucosal pinch biopsies were similarly collected from colonoscopy patients with no history of IBD undergoing surveillance procedures due to age or family history (healthy controls, HC). Prior to colonoscopy, *Picoprep* bowel preparation was used, which contains active ingredients sodium picosulfate, magnesium carbonate hydrate, and citric acid. All patients were Australian and of typically white European descent (Supplementary Data 2) and had undergone no antibiotic use in the 28 days prior to colonoscopy. Inflammatory state was recorded by the treating gastroenterologists during colonoscopy and histologically confirmed by anatomical pathologists with subspecialty interest in gastrointestinal pathology (Nancy Scores indicated in Supplementary Data 2). Mucosal pinch biopsies were immediately transferred to ice-cold RPMI medium containing 10% heat-inactivated (HI) faecal bovine serum (FBS, #SFBS-AU, Bovogen).

### 16S rRNA gene sequencing

Colonic biopsies were washed in 1x PBS and cryopreserved at −80C in FBS containing 10% DMSO. For DNA isolation, biopsies were washed in PBS and digested overnight in a thermoshaker at 700 rpm/56 °C in 180 µL Buffer ATL (#56304, QIAGEN) with 30 µL proteinase K (QIAGEN). DNA extraction was then performed using the QIAamp DNA Mini Kit (#56304, QIAGEN) as per the manufacturer’s instructions. The V3-V4 hypervariable region of the 16S rRNA gene was targeted for amplicon sequencing using the 341f-805r primer pair (forward 5’-

TCGTCGGCAGCGTCAGATGTGTATAAGAGACAGCCTACGGGNGGCWGCAG, reverse 5’-

GTCTCGTGGGCTCGGAGATGTGTATAAGAGACAGGACTACHVGGGTATCTAATCC) as outlined in Illumina’s (San Diego, CA) 16 s Metagenomic Sequencing Library Preparation guide^68^. Due to low microbial biomass in biopsy samples, five PCR reactions per biopsy sample were run in the 1st stage PCR. These five reactions were combined, and 260-575 base pair amplicon selection was performed using the SPRIselect bead-based reagent kit (#B23317, Beckman Coulter). 16S amplicon libraries were prepared and sequenced on a MiSeq i100 Plus system to generate paired-end 300 bp reads at the Ramaciotti Centre for Genomics (UNSW).

### Analysis of microbial amplicon sequencing

16S sequencing was processed using QIIME2 2025.7 amplicon distribution^69^, and host-derived reads were removed using the inbuilt filter functions and GRCh38.p14 human genome assembly as a reference. The filtered reads were then denoised using dada2^70^. Taxonomic annotation was performed using QIIME2 feature-classifier and the Silva v138.1 database as a reference. The relative abundances and diversity metrics were then analysed using the phyloseq package in R^71^. Relative abundances at the genus level were calculated for each biopsy by dividing genus counts by total counts. Adonis2 PERMANOVA test from the vegan package was used to rank the effect of disease on microbial composition in Bray-Curtis distances. To analyse the differences in genus abundance between diseases, differential abundance analysis was performed using DESeq2 package^72^ with correction for biopsy location and donor sex. For differential abundance analysis in each region, genus-level counts were used to create an Anndata object in Scanpy (version 1.9.1) with metadata as observations and genera as variables and counts normalised per biopsy and log transformed. Wilcoxon and Kruskal-Wallis tests were used to compare feature abundances and diversity measures. Where appropriate, *P* values were adjusted using the Benjamin-Hochberg method^70^.

### Single cell RNA-sequencing library preparation

Two to three mucosal pinch biopsies per colon region per patient/individual were processed for single-cell RNA sequencing either immediately following colonoscopy or from cryopreserved samples as detailed in Supplementary Data 3. Biopsies from each colon region were processed separately until pooled for capture and were demultiplexed either via incorporation of hashtag oligos or single nucleotide polymorphism information (detailed in Supplementary Data 25). Biopsies were washed in 1x DPBS (#14040133, Gibco) before incubation in 1.5 mL digestion medium (1.16 mL HBSS (#14025092, Gibco), 246.9 µL liberase DH (#LIBDH-RO, Roche), 60 µL hyaluronidase, 36 µL DNase I) in 15 mL tubes with rotation at 600 rpm for 25 min at 37 °C. Biopsies were homogenised for 10 min via trituration and vortexing. Digests were halted by adding 5 mL of ice-cold neutralisation medium (20% HI-FBS in RPMI 1640 (#11875093, Gibco) and cell suspensions passed through 40 µM cell strainers. Digestion tubes were washed with a further 5 mL neutralisation medium passed through the same strainer to transfer any remaining cells. Cell suspensions were then centrifuged at 300 g for 10 min at 4 °C. Supernatants were discarded and cells resuspended in 1 mL resuspension medium (1% FBS in DPBS) and centrifuged again as before. For sample hashing, supernatants were then discarded, and cell pellets resuspended in 45 µL Cell Staining Buffer (#420201, BioLegend) with 5 µL TruStain Fc Blocking Reagent (#422301, BioLegend) and incubated for 10 min at 4 °C. TotalSeq C0251 anti-human hashtag 1 (#394661, BioLegend) and C0252 anti-human hashtag 2 (#394663, BioLegend) antibody pools were prepared as per the manufacturer’s instructions and added to 50 µL blocked cell suspensions and incubated for 30 min at 4 °C. Hashed cell suspensions were then washed three times by centrifugation at 400 g at 4 °C for 5 min with 3 mL Cell Staining Buffer. Cell pellets were then resuspended in 100 µL resuspension medium and counted.

### Single nucleotide polymorphism genotyping and preprocessing

DNA was isolated from dissociated cells using the DNeasy Blood & Tissue Kit (QIAGEN) and submitted for single nucleotide polymorphism (SNP) genotyping using the UK Biobank Axiom Array (Thermo Fisher Scientific) at the Ramaciotti Centre for Genomics, University of New South Wales. The Axiom Analysis Suite (5.3.0.45) from Thermo Fisher Scientific was used to generate VCF files from raw.CEL files. Imputation was run using the SNP_imputation_100g_hg38 pipeline^73^ with the 1000 G Project as the reference and variants filtered for minor allele frequency > 0.05 using bcftools^74^.

### Preprocessing of scRNA-seq data

The Cell Ranger (v9.0) *cellranger multi* analysis pipeline with default settings was used to generate counts files from gene expression and V(D)J library files, specifying BioLegend hashtag barcodes in the feature reference file where relevant as recommended in official Cellranger documentation. SNP information was used to demultiplex patients using the Souporcell^75^ tool within the Demuxafy^76^ framework and information read into the Anndata object. Raw gene expression and V(D)J sequencing data from healthy adult control patients^14^ (Array Express E-MTAB-9543, patient IDs 411c, 414c, 417c, 432c, and 440c) were similarly processed from fastq files using Cell Ranger (v9.0). All further single-cell analyses were performed using the Scanpy package^77^ (v1.9.1) in Python (v3.9.4) with all function parameters at default settings unless described otherwise. Filtered counts files from each capture were first subjected to doublet detection using Scrublet with a manually-adjusted doublet score cut-off threshold determined using the scrub.plot_histogram() function. Individual counts matrices were merged into a single AnnData object and cells were filtered for more than 200 genes and less than 50% mitochondrial reads. Genes expressed in fewer than three cells were also removed, as well as mitochondrial and ribosomal genes. The dataset was then normalised and log-transformed before being filtered for highly variable genes determined using the standard Scanpy workflow. The number of counts per cell and percentage of mitochondrial genes expressed were regressed before data scaling and principal component analysis. Batch-corrected nearest neighbours and dimensionality reduction were performed using Scanpy’s bbknn and UMAP functions.

### Cell type annotation and analysis of scRNA-seq data

Leiden clustering was first performed at resolution 0.3 on full datasets, and highly expressed genes for each cluster were defined using the sc.tl.rank_gene_groups() function and used to manually annotate broad cell lineages based on established marker genes from previously published data. Broad cell lineage annotations were then read into the original, non-normalised counts files, which were subsequently sliced into separate counts files based on cell lineage. For each cell lineage, batches with fewer than three cells were removed to allow appropriate batch-corrected nearest neighbours via sc.external.pp.bbknn. Individual lineage files were then further subclustered and annotated with increased resolution as described above. While the full Anndata object was used to annotate cell types, only cells from noninflamed regions were included for cell type proportion analyses unless otherwise stated. Differential abundance analysis was performed in MiloR^78^, which assigns cells to partially overlapping neighbourhoods on a k-nearest neighbour graph and performs multiple-comparison hypothesis testing with SpatialFDR correction. Milo neighbourhoods were assigned to a cell type if more than 70% of its cells belonged to it. The abundance of a cell type was considered significant if the absolute value of the median fold change of its neighbourhoods was greater than 1.7. For comparing left colon vs. right colon, cell type proportions were calculated for pooled ascending and transverse (right colon) and descending and rectum (left colon) samples, and a paired t-test was used to compare between left and right regions of the same patient. Due to unavoidable differences in transcriptional signatures between fresh and frozen batches for UC and PSC-UC samples (Supplementary Data 25), we performed differential gene expression analyses between disease cohorts on each batch individually (via sc.tl.rank_genes_groups specifying Wilcoxon rank sum), and considered only genes that were identified as significantly altered (adjusted *p* value of <0.05) in the same direction in both frozen and fresh batches. Our previously published HC data were incorporated for both fresh and frozen batch differential gene expression analyses. We reasoned that this method ensures technical robustness whilst avoiding unnecessary data manipulation. For ligand-receptor prediction, the full raw dataset was subsetted by disease and inflammatory state, normalised, and analysed using CellphoneDB’s (v5.0.1) statistical method^79^. Cell type proportions and spatial deconvolution scores were visualised using seaborn boxplots, violin plots, and stripplots in Python. Statistical comparisons were performed using SciPy (scipy.stats), including paired Student’s t-tests (ttest_rel), unpaired Student’s t-tests (ttest_ind), and Mann–Whitney U tests (mannwhitneyu) as appropriate for each analysis.

### TCR analysis

TCR analysis was performed using the SCIRPY package (v0.22.3)^80^ on the T cell lineage Anndata object using default parameters. Note that V(D)J library preparation failed for patient PSC-1, so this patient is excluded from TCR analyses. For analyses on noninflamed patients only, we excluded patients experiencing inflammation in any region due to immune cell motility.

### Gene ontology and pathway analysis

Lists of cell type-specific differentially expressed genes were generated using SCANPY’s sc.tl.rank_genes_groups function, and significantly upregulated gene lists were used to generate analysis reports on the Metascape^81^ web browser using the ‘Express Analysis’ function. Results were downloaded and depicted graphically using Prism (v10).

### Deconvolution from bulk data

Published bulk RNA sequencing count files^8^ were manually sliced according to disease and inflammatory state and deconvoluted using our own single-cell RNA sequencing data using the cellanneal^82^ (v1.1.0) command-line tool as per the relevant documentation. Relative percentages of cell types in each bulk dataset were displayed graphically using Prism.

### BCR analysis

For UC and PSC-UC samples, Cell Ranger V(D)J contigs for B cells were post-processed with stand-alone IgBLAST^83^ (v1.21) aligning against the IMGT human reference directory (release 202330-1). For cells with multiple heavy or light chains, the productive chain with the highest UMI count was retained. Non-productive reads, reads lacking a full-length IGHV and those without a defined CDR3 were discarded from analysis. Only cells with paired heavy and light chains were used for analysis. B cell clonal lineages were built with SCOPer^84^ (v1.3.0) using single linkage for the CDR3 nucleotide sequences at a clustering threshold of 0.2, with clones defined by both heavy and light chains. For previously published healthy controls^14^, FASTQs were obtained from ArrayExpress under accession E-MTAB-9532 and processed with Cell Ranger’s *cellranger vdj* (v8.0.0). V(D)J contigs were post-processed and filtered as for UC and PSC-UC samples. Datasets were merged and summarised in R (v4.3.0) using RStudio (version 2024.04.2 + 764) utilising the tidyverse package^85^ (v2.0.0).

### Spatial transcriptomics data generation

STOmics *Stereo-seq Transcriptomics Set for Chip-on-a-slide user manual* (version B) was applied to optimal cutting temperature medium (OCT)-embedded fresh-frozen colonic mucosa biopsies. All samples were sectioned using the Leica CM1860 UV cryostat, and two to three serial cuts were included on each Stereo-seq slide. Tissue samples were selected based on disease and inflammation status of the colon region and morphology (based on H&E). Tissue sections were adhered to the Stereo-seq chip and incubated in −20 °C methanol (#34860, Sigma) for 30 minutes. Chip IDs used in this study were D01654C4 (UC noninflamed), D01654C5 (UC inflamed) and D01654C2 (PSC noninflamed). Stereo-seq slides were fluorescently stained with Qubit ssDNA Assay Kit (#Q10212, ThermoFisher) as per the manual and imaged using a Leica DM6 microscope with a CTR6 LED electronics box. The Stereo-seq *Permeabilisation Set for Chip-on-a-slide user manual* (version B) was followed to obtain an optimal permeabilisation time for colonic mucosa tissue of 8 minutes. The cDNA was purified using SPRIselect Beads (#B23318, Beckman Coulter). cDNA quality was quantified before and following cDNA library preparation using a Qubit 4 fluorometer with Qubit dsDNA mix (#Q33230, Invitrogen) and Agilent Tapestation High Sensitivity D5000 ScreenTape Assay (#5067-5592 & #5067-5593, Agilent Technologies). cDNA libraries were pooled to a final concentration of 13.0 ng/µL (24.63 µL volume) and sequenced on one MGI G400 FCL PE100 flow cell at the South Australian Genomics Centre.

### Spatial transcriptomics data analysis

Raw FASTQ files were processed using the Stereo-seq Analysis Workflow (SAW) pipeline (BGI). Stereopy (v1.5.1) in Python (v3.8.20) was used to create individual Anndata objects per tissue section with a bin size of 20 (approximately 10 x 10 µm). TACCO (v0.4.0.post1) was used to score spatial coordinates for each cell type using the tc.tl.annotate function with our scRNA-seq dataset as a reference. A Mann-Whitney U test was used to test for significant differences between spot scores for each sample. Hard labels per coordinate were assigned using the highest scoring cell type per spot, and Squidpy (v1.6.3) was used to score colocalisation between cell types using the sq.gr.spatial_neighbors and sq.gr.nhood_enrichment functions.

### Immunofluorescence

Immunofluorescence analysis was performed on study patient FFPE tissue (as indicated in Supplementary Data 3) mounted on slides with 30-minute antigen retrieval at pH 6. Slides were rinsed 3 times for 10 min in PBS, followed by two 10-minute incubations in permeabilisation buffer (250 µL PBS, 200 µL H_2_O, 50 µL 2% bovine gelatin (#G9391, Sigma-Aldrich) solution, 1.25 mL Triton-X 100 (#TA9H9A9A7460, Sigma-Aldrich), and 1 h blocking with 5% bovine serum albumin (BSA, #A4737, Sigma-Aldrich) in permeabilisation buffer. Slides were treated with primary antibodies in 1% BSA permeabilisation buffer overnight, rinsed in 10-minute intervals twice with PBS and once with permeabilisation buffer before secondary antibody solution treatment, also in 1% BSA^86^. Primary and secondary antibody concentrations were as follows: C-KIT, 1:2000 (3306 s, Cell Signalling); TMEM176B, 1:100 (ab236860, Abcam), goat anti-rabbit IgG AF594, 1:400 (ab150080, Abcam), goat anti-mouse IgG1 AF488, 1:400 (ab150113, Abcam). Nuclei were stained via addition of DAPI at 1:1000 during secondary antibody incubation. Two drops of Prolong Diamond Antifade Mountant (#P36961, ThermoFisher) were added to stained sections before mounting coverslips. Slides were imaged in a single plane on a Leica SP8 DMI6000 confocal imaging system using 20x/0.7 immersion and HC PL APO CD2 40x/1.30 oil objectives, and diode 405 nm, OPSL 488 nm, OPSL 552 nm and diode 638 nm lasers with PMT detectors. Images were processed and analysed using Imaris software (Bitplane). For quantification of IMCs, mast cells were defined using DAPI and c-KIT fluorescence, and mean AF594 fluorescence (TMEM176B) was calculated per mast cell.

### FcERI activation of mast cells

The Cl.MC/C57.1 cell line^38^ (Galli cells; generated from the BL6 mouse and generously gifted by Professors John Hunt and Stephen Galli) were cultured in DMEM (high glucose and pyruvate, Gibco #11995073) supplemented with 10% FBS (SFBS, Bovogen) heat-inactivated at 56 °C for 30 minutes, 1x MEM nonessential amino acids (#11140050, Gibco), 2 mM L-glutamine (#25030081, Gibco) penicillin-streptomycin (#PO781, Sigma), 36 mg/L L-asparagine, 87.1 ng/L L-arginine, 6 mg/L folic acid and 50 µM 2-ME. Galli cells were suspended in complete medium at 1 million cells/mL, and 15 mL was used for each condition (5 mL/replicate). For sensitisation^87^, cells were incubated overnight with 0.1 µg DNP-IgE (#D8406, Sigma-Aldrich) per mL cell suspension. Individual samples (5 mL) were then pelleted and resuspended in 2 mL complete medium for a second 20-minute sensitisation at 0.2 µg DNP-IgE per mL. Cells were then centrifuged and washed twice with PGE buffer^88^ (25 mM PIPES (#P6757, Sigma-Aldrich), 120 mM NaCl, 5 mM KCl, 50 mM NaOH, 5.6 mM glucose (#A2494001, Gibco), 0.2% BSA, 1 mM CaCl_2_). To induce degranulation, cells were suspended in 300 µL PGE buffer containing 100 ng/mL DNP-HSA (#A6661, Sigma) and incubated for 1 hour at 37 °C with 5% CO_2_. Control samples were resuspended in PGE with no DNP-HSA. For BayK8644 and vehicle samples, cells were subjected to 30 minutes pre-incubation with 10 mM BayK8644 (#B112, Sigma-Aldrich) or equivalent volume DMSO before sensitisation and 10 µM BayK8644 was maintained in complete medium until degranulation.

### Assessment of mast cell degranulation

Cell suspensions were counted and centrifuged at 300 rpm and supernatants were harvested. To determine supernatant β-hexosaminidase content, 25 µL of supernatant was combined with 25 µL 5 mM P-nitrophenyl N-acetyl-β-D-glucosaminide (#N9376, Sigma-Aldrich) in 50 mM sodium citrate (pH 4.5) and incubated at 37 °C for 1 h^87^. After 1 hour, reactions were terminated with 250 µL 0.2 M glycine-NaOH (pH 10.6) and absorbance at 405 nm determined using a Nanodrop. Due to uptake of fluorescent BayK8644 within cells, cell lysate β-hexosaminidase content could not be accurately measured via spectrophotometry. Instead, degranulation was measured as supernatant β-hexosaminidase content (A405) per 10^6^ cells.

### Quantitative real-time PCR

RNA was isolated from fresh cell lysates using the RNeasy Mini Kit (#74104, QIAgen) as per manufacturer’s instructions. RNA concentrations were measured via Nanodrop and cDNA generated from 2 µg RNA using the High-Capacity cDNA Reverse Transcription Kit (#4368814, ThermoFisher Scientific) as per manufacturer’s instructions. RT-qPCR was performed in 10 µL reactions containing 2 µg cDNA, 5 µL 2X HotStart PCR Master Mix (HMM, MCLAB), 0.7 µL SYTO9 and mouse primer pairs topped up with nuclease-free H_2_O. Primer information and concentrations are shown in Supplementary Table 1. Reactions were analysed in a LightCycler 480 System (Roche) with 15 min heat inactivation at 95 °C followed by 45 PCR cycles (15 s at 95 °C, 30 s at 58 °C, 15 s at 72 °C).

### Reporting summary

Further information on research design is available in the Nature Portfolio Reporting Summary linked to this article.

## Supplementary information

Supplementary Information

Description of Additional Supplementary Files

Supplementary Data 1

Supplementary Data 2

Supplementary Data 3

Supplementary Data 4

Supplementary Data 5

Supplementary Data 6

Supplementary Data 7

Supplementary Data 8

Supplementary Data 9

Supplementary Data 10

Supplementary Data 11

Supplementary Data 12

Supplementary Data 13

Supplementary Data 14

Supplementary Data 15

Supplementary Data 16

Supplementary Data 17

Supplementary Data 18

Supplementary Data 19

Supplementary Data 20

Supplementary Data 21

Supplementary Data 22

Supplementary Data 23

Supplementary Data 24

Supplementary Data 25

Reporting Summary

Transparent Peer Review file

## Source data

Source Data

## Data Availability

The raw single-cell gene expression sequencing data generated in this study are available at ArrayExpress under accession numbers E-MTAB-14955 and E-MTAB-16401. The raw single-cell V(D)J sequencing data are available at ArrayExpress under accession numbers: E-MTAB-14901, E-MTAB-14899, E-MTAB-17148, and E-MTAB-17213. Processed single-cell gene expression and V(D)J sequencing data are available at CELLxGENE at https://cellxgene.cziscience.com/collections/75e35754-bd21-4562-a4f8-39eaa4dfce36. Raw and processed STOmics spatial transcriptomics data are available at ArrayExpress under accession number E-MTAB-17162. Raw 16S ribosomal RNA sequencing data are available at ENA under accession number PRJEB86073 (ERP169448) and processed genus-level counts are available in Supplementary Data 4 of this manuscript. All data are included in the Supplementary Information or available from the authors, as are unique reagents used in this Article. The raw numbers for charts and graphs are available in the Source Data file whenever possible. Previously published raw single-cell gene expression and V(D)J sequencing data were accessed from ArrayExpress under accession numbers E-MTAB-9543 and E-MTAB-9532. Source data are provided with this paper.

## Code availability

Custom code used for SNP imputation^73^ is accessible via Zenodo [https://zenodo.org/records/20634235]
