# Supplementary material for: Primary sclerosing cholangitis displays distinct colonic mucosa topography yet a shared mast cell state with ulcerative colitis
Source: Nat Commun. 2026 Jul 7;17:10107. doi: 10.1038/s41467-026-75231-1 (PMC13597510; doi:10.1038/s41467-026-75231-1)
Supplement: Supplementary file 1 [file 41467_2026_75231_MOESM1_ESM.pdf]

Supplementary Information

Primary sclerosing cholangitis colonic mucosa displays distinct topography yet a shared mast cell state with ulcerative colitis

Jacqueline LE Tearle, Ekaterina Sviriaeva, Fan Zhang, Katherine JL Jackson, Joshua Kaye, Paris Tavakoli, Sabrina Koentgen, Joanna Warren, Raymond R. Liang, Pratibha Malhotra, Cameron Williams, Ashraful Haque, Arteen Arzivian, Kavitha K. Sudhakar, Drew Neavin, Nicodemus Tedla, Andrew Kim, Craig Haifer, Hamish W King, Georgina L Hold, Simon Ghaly, Kylie R James*

***Corresponding author:** Translational Genomics Program, Garvan Institute of Medical Research, The Kinghorn Cancer Centre, 370 Victoria Street, Darlinghurst, NSW, 2010. k.james@garvan.org.au

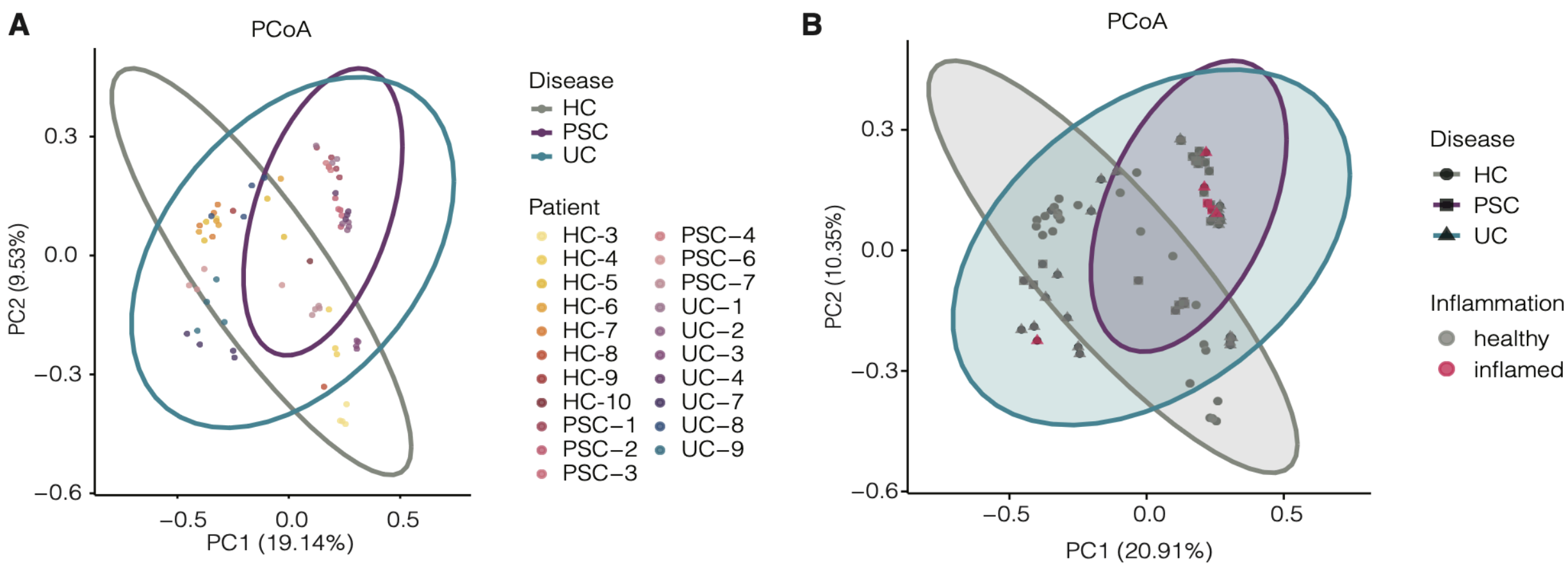

Supplementary Figure 1

Principal coordinate analysis (PCoA) plot of 16S rRNA gene sequencing of colonic mucosa biopsies from the ascending, transverse, descending colon and rectum of UC, PSC-UC and HC patients coloured by patient (**A**) and inflammation status (**B**). Each dot represents a single biopsy.

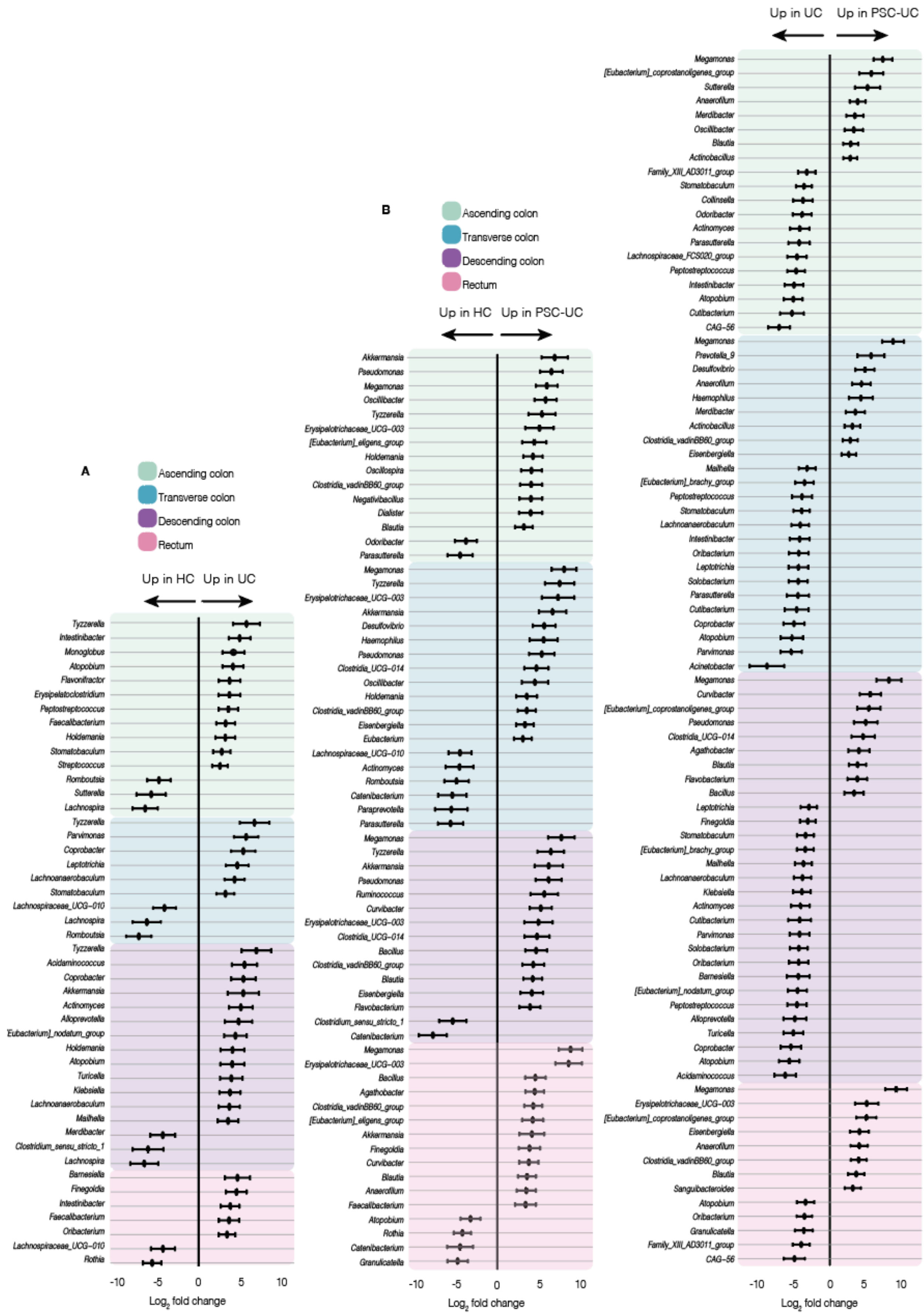
A
Ascending colon
Transverse colon
Descending colon
Rectum
Up in HC
Up in UC
Tyzzerella
Intestinibacter
Monoglobus
Atopobium
Flavonifractor
Erysipelatoclostridium
Peptostreptococcus
Faecalibacterium
Holdemania
Stomatobaculum
Streptococcus
Romboutsia
Sutterella
Lachnospira
Tyzzerella
Parvimonas
Coprobacter
Leptotrichia
Lachnoanaerobaculum
Stomatobaculum
Lachnospiraceae_UCG−010
Lachnospira
Romboutsia
Tyzzerella
Acidaminococcus
Coprobacter
Akkermansia
Actinomyces
Alloprevotella
[Eubacterium]_nodatum_group
Holdemania
Atopobium
Turicella
Klebsiella
Lachnoanaerobaculum
Mailhella
Merdibacter
Clostridium_sensu_stricto_1
Lachnospira
Barnesiella
Finegoldia
Intestinibacter
Faecalibacterium
Oribacterium
Lachnospiraceae_UCG−010
Rothia
-10 -5 0 5 10
Log2 fold change
B
Ascending colon
Transverse colon
Descending colon
Rectum
Up in HC
Up in PSC-UC
Akkermansia
Pseudomonas
Megamonas
Oscillibacter
Tyzzerella
Erysipelotrichaceae_UCG−003
[Eubacterium]_eligens_group
Holdemania
Oscillospira
Clostridia_vadinBB60_group
Negativibacillus
Dialister
Blautia
Odoribacter
Parasutterella
Megamonas
Tyzzerella
Erysipelotrichaceae_UCG−003
Akkermansia
Desulfovibrio
Haemophilus
Pseudomonas
Clostridia_UCG−014
Oscillibacter
Holdemania
Clostridia_vadinBB60_group
Eisenbergiella
Eubacterium
Lachnospiraceae_UCG−010
Actinomyces
Romboutsia
Catenibacterium
Paraprevotella
Parasutterella
Megamonas
Tyzzerella
Akkermansia
Pseudomonas
Ruminococcus
Curvibacter
Erysipelotrichaceae_UCG−003
Clostridia_UCG−014
Bacillus
Clostridia_vadinBB60_group
Blautia
Eisenbergiella
Flavobacterium
Clostridium_sensu_stricto_1
Catenibacterium
Megamonas
Erysipelotrichaceae_UCG−003
Bacillus
Agathobacter
Clostridia_vadinBB60_group
[Eubacterium]_eligens_group
Akkermansia
Finegoldia
Curvibacter
Blautia
Anaerofilum
Faecalibacterium
Atopobium
Rothia
Catenibacterium
Granulicatella
-10 -5 0 5 10
Log2 fold change
Up in UC
Up in PSC-UC
Megamonas
[Eubacterium]_coprostanoligenes_group
Sutterella
Anaerofilum
Merdibacter
Oscillibacter
Blautia
Actinobacillus
Family_XIII_AD3011_group
Stomatobaculum
Collinsella
Odoribacter
Actinomyces
Parasutterella
Lachnospiraceae_FCS020_group
Peptostreptococcus
Intestinibacter
Atopobium
Cutibacterium
CAG−56
Megamonas
Prevotella_9
Desulfovibrio
Anaerofilum
Haemophilus
Merdibacter
Actinobacillus
Clostridia_vadinBB60_group
Eisenbergiella
Mailhella
[Eubacterium]_brachy_group
Peptostreptococcus
Stomatobaculum
Lachnoanaerobaculum
Intestinibacter
Oribacterium
Leptotrichia
Solobacterium
Parasutterella
Cutibacterium
Coprobacter
Atopobium
Parvimonas
Acinetobacter
Megamonas
Curvibacter
[Eubacterium]_coprostanoligenes_group
Pseudomonas
Clostridia_UCG−014
Agathobacter
Blautia
Flavobacterium
Bacillus
Leptotrichia
Finegoldia
Stomatobaculum
[Eubacterium]_brachy_group
Mailhella
Lachnoanaerobaculum
Klebsiella
Actinomyces
Cutibacterium
Parvimonas
Solobacterium
Oribacterium
Barnesiella
[Eubacterium]_nodatum_group
Peptostreptococcus
Alloprevotella
Turicella
Coprobacter
Atopobium
Acidaminococcus
Megamonas
Erysipelotrichaceae_UCG−003
[Eubacterium]_coprostanoligenes_group
Eisenbergiella
Anaerofilum
Clostridia_vadinBB60_group
Blautia
Sanguibacteroides
Atopobium
Oribacterium
Granulicatella
Family_XIII_AD3011_group
CAG−56
-10 -5 0 5 10
Log2 fold change

Supplementary Figure 2

A | Differentially abundant (adjusted p value < 0.05) bacterial genera per colonic region for UC vs. HC. **B |** Differentially abundant (adjusted p value < 0.05) bacterial genera per colonic region for PSC-UC vs. HC. **C |** Differentially abundant (adjusted p value < 0.05) bacterial genera per colonic region for PSC-UC vs. UC. For HC, n= 6 for ascending colon, 5 for descending colon, 5 for transverse colon, and 7 for rectum. For UC, n= 7 for ascending colon, 4 for descending colon, 6 for transverse colon, and 6 for rectum. For PSC-UC, n= 5 for ascending colon, 5 for descending colon, 4 for transverse colon, and 5 for rectum. Points indicate the log2 fold change (log2FoldChange) in relative abundance for each taxon, estimated using DESeq2's negative binomial generalized linear model. Horizontal lines represent the 95% confidence interval, calculated as log2FoldChange ± 1.96 × lfcSE (the standard error of the model-based fold-change estimate).

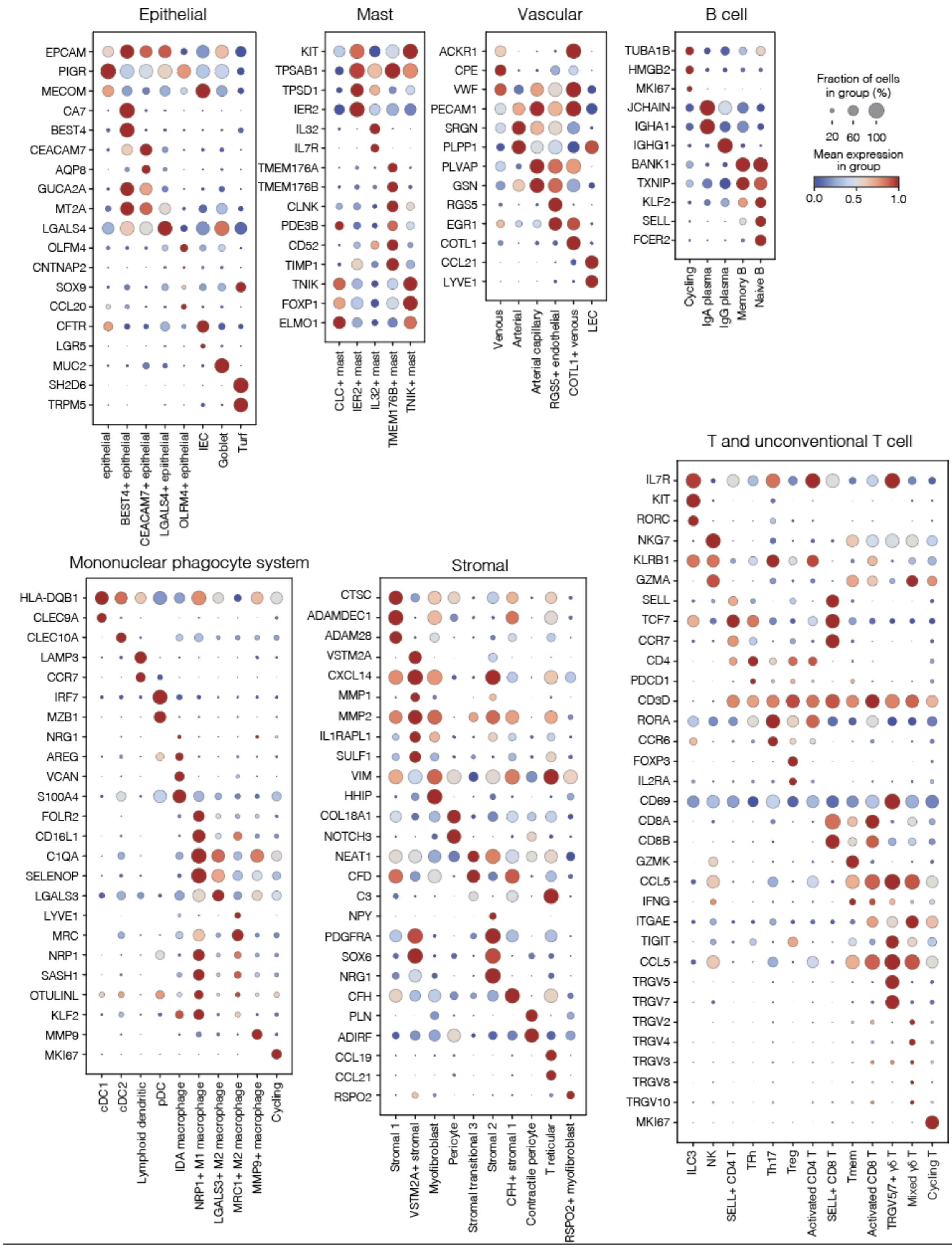

Epithelial
EPCAM
PIGR
MECOM
CA7
BEST4
CEACAM7
AQP8
GUCA2A
MT2A
LGALS4
OLFM4
CNTNAP2
SOX9
CCL20
CFTR
LGR5
MUC2
SH2D6
TRPM5
epithelial
BEST4+ epithelial
CEACAM7+ epithelial
LGALS4+ epithelial
OLFM4+ epithelial
IEC
Goblet
Turf
Mast
KIT
TPSAB1
TPSD1
IER2
IL32
IL7R
TMEM176A
TMEM176B
CLNK
PDE3B
CD52
TIMP1
TNIK
FOXP1
ELMO1
CLC+ mast
IER2+ mast
IL32+ mast
TMEM176B+ mast
TNIK+ mast
Vascular
ACKR1
CPE
VWF
PECAM1
SRGN
PLPP1
PLVAP
GSN
RGS5
EGR1
COTL1
CCL21
LYVE1
Venous
Arterial
Arterial capillary
RGS5+ endothelial
COTL1+ venous
LEC
B cell
TUBA1B
HMGB2
MKI67
JCHAIN
IGHA1
IGHG1
BANK1
TXNIP
KLF2
SELL
FCER2
Cycling
IgA plasma
IgG plasma
Memory B
Naive B
Fraction of cells in group (%)
20 60 100
Mean expression in group
0.0 0.5 1.0
T and unconventional T cell
IL7R
KIT
RORC
NKG7
KLRB1
GZMA
SELL
TCF7
CCR7
CD4
PDCD1
CD3D
RORA
CCR6
FOXP3
IL2RA
CD69
CD8A
CD8B
GZMK
CCL5
IFNG
ITGAE
TIGIT
CCL5
TRGV5
TRGV7
TRGV2
TRGV4
TRGV3
TRGV8
TRGV10
MKI67
ILC3
NK
SELL+ CD4 T
Tfh
Th17
Treg
Activated CD4 T
SELL+ CD8 T
Tmem
Activated CD8 T
TRGV5/7+ γδ T
Mixed γδ T
Cycling T
Mononuclear phagocyte system
HLA-DQB1
CLEC9A
CLEC10A
LAMP3
CCR7
IRF7
MZB1
NRG1
AREG
VCAN
S100A4
FOLR2
CD16L1
C1QA
SELENOP
LGALS3
LYVE1
MRC
NRP1
SASH1
OTULINL
KLF2
MMP9
MKI67
cDC1
cDC2
Lymphoid dendritic
pDC
IDA macrophage
NRP1+ M1 macrophage
LGALS3+ M2 macrophage
MRC1+ M2 macrophage
MMP9+ macrophage
Cycling
Stromal
CTSC
ADAMDEC1
ADAM28
VSTM2A
CXCL14
MMP1
MMP2
IL1RAPL1
SULF1
VIM
HHIP
COL18A1
NOTCH3
NEAT1
CFD
C3
NPY
PDGFRA
SOX6
NRG1
CFH
PLN
ADIRF
CCL19
CCL21
RSPO2
Stromal 1
VSTM2A+ stromal
Myofibroblast
Pericyte
Stromal transitional 3
Stromal 2
CFH+ stromal 1
Contractile pericyte
T reticular
RSPO2+ myofibroblast

Supplementary Figure 3

Marker genes for cell types detected in the scRNA-seq dataset separated by broad lineage.

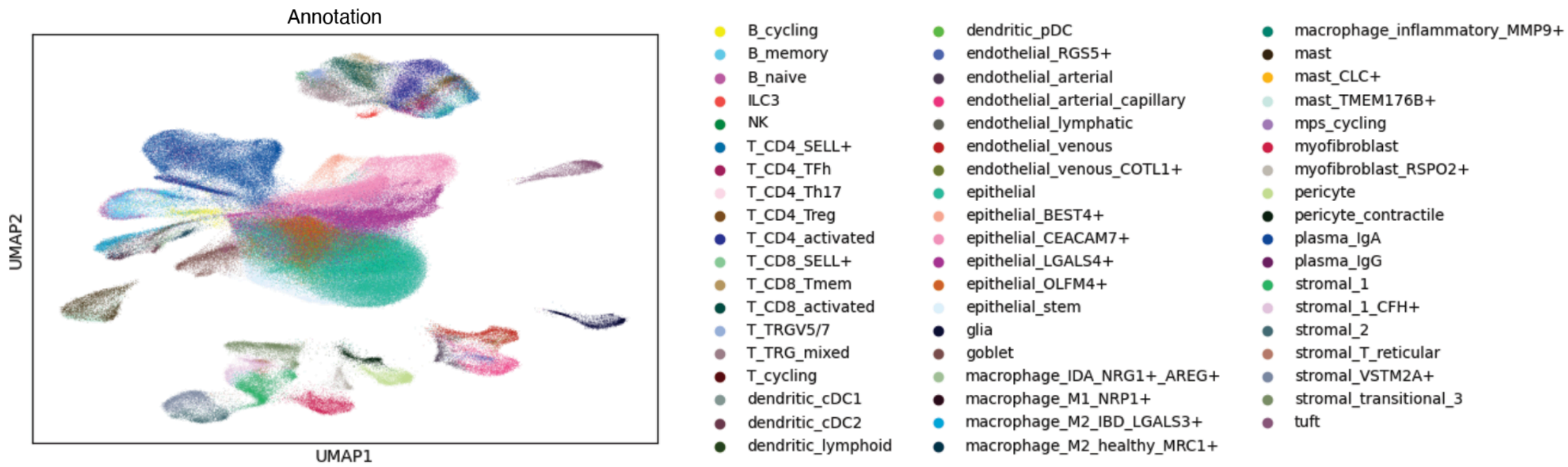

Supplementary Figure 4

A | UMAP of the entire scRNA-sequencing dataset coloured by cell type annotation.

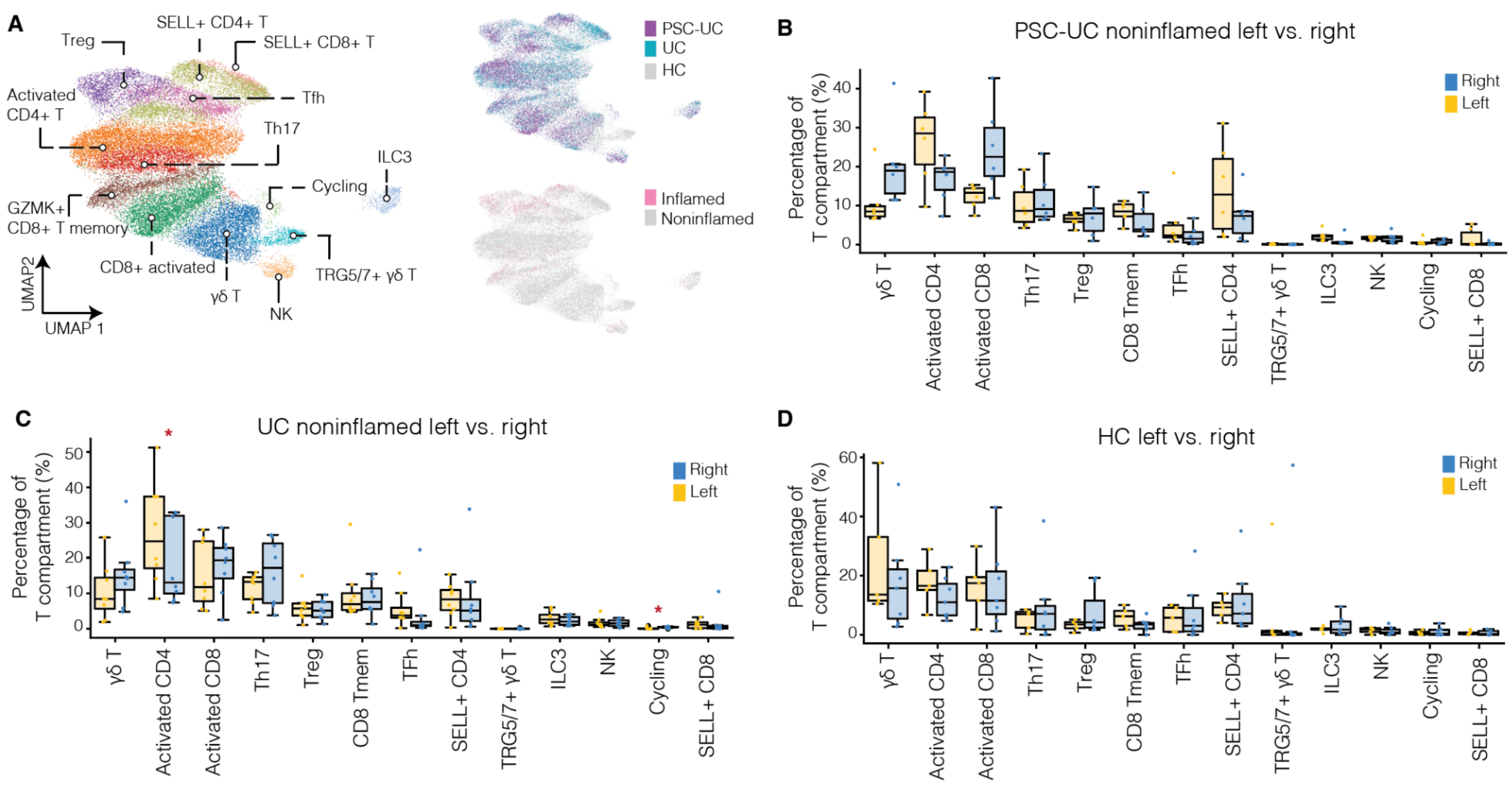

Supplementary Figure 5

A | UMAP of the T cell compartment coloured by T cell subtype annotation, disease and inflammation. **B-D |** Box plots for relative cell type proportions for T cell subsets for noninflamed samples in the left and right colons of PSC-UC (B), UC (C) and HC (D) donors. Each dot represents pooled ascending and transverse (right) or pooled descending and rectum (left) biopsies of a single patient. Boxes show median and interquartile range and whiskers show minimum and maximum, excluding outliers.

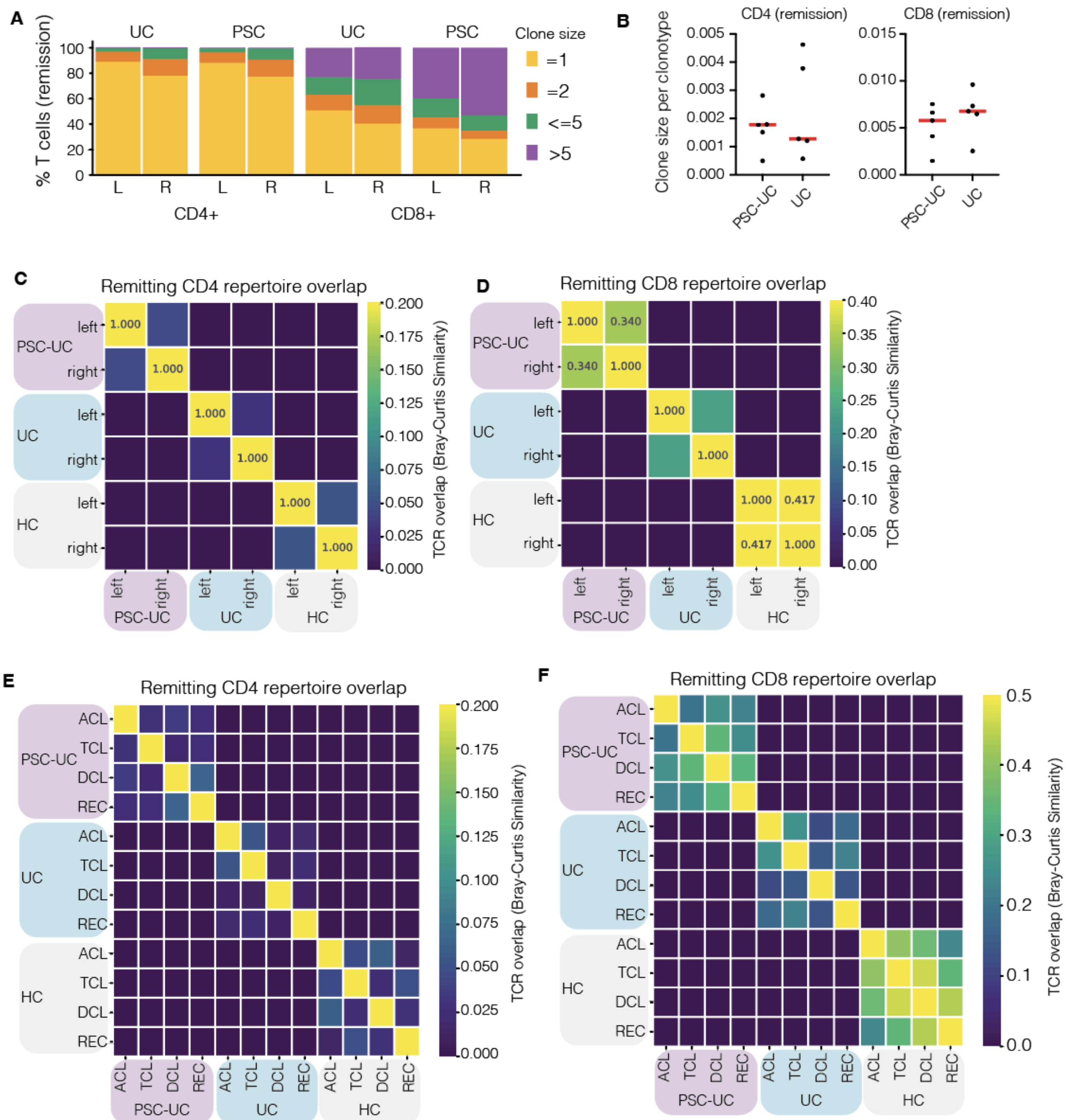

Supplementary Figure 6

A | Bar charts displaying percentage UC and PSC-UC CD4 and CD8 T cells in the right and left colon by clonotype size. **B |** Dot plots displaying average clone size normalised to the total number of CD4 or CD8 T cells in the noninflamed dataset. Each dot represents combined biopsies from one noninflamed patient. N=5 for both PSC-UC and UC. **C |** Heatmap displaying left colon vs. right colon repertoire overlap for noninflamed CD4 T cells as determined by Bray-Curtis similarity where 1 indicates that repertoires of two regions are identical and 0

indicates they are completely distinct. N=5 for both PSC-UC and UC. **D |** Heatmap displaying left colon vs. right colon repertoire overlap for noninflamed CD8 T cells as determined by Bray-Curtis similarity where 1 indicates that repertoires of two regions are identical and 0 indicates they are completely distinct. N=5 for both PSC-UC and UC. **E |** Heatmap displaying ascending colon, transverse colon, descending colon and rectum repertoire overlap for noninflamed CD4 T cells as determined by Bray-Curtis similarity where 1 indicates that repertoires of two regions are identical and 0 indicates they are completely distinct. N=5 for both PSC-UC and UC. **F |** Heatmap displaying ascending colon, transverse colon, descending colon and rectum repertoire overlap for noninflamed CD8 T cells as determined by Bray-Curtis similarity where 1 indicates that repertoires of two regions are identical and 0 indicates they are completely distinct. N=5 for both PSC-UC and UC.

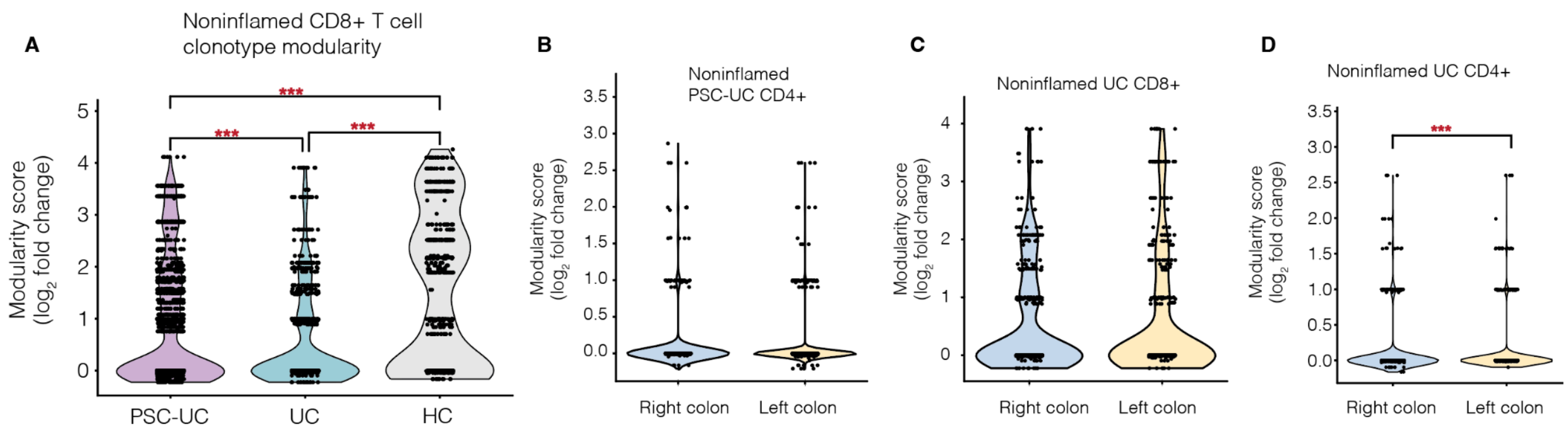

Supplementary Figure 7

A | Noninflamed CD8 T cell clonotype modularity. P values as determined by two-sided Mann-Whitney U test followed by Benjamini-Hochburg correction = $1.8x10^{-5}$ for PSC-UC vs. UC, $1.7x10^{-26}$ for PSC-UC vs. HC and $2.97x10^{-36}$ for UC vs. HC. Eat dot represents one cell. N = **B |** Violin plots displaying CD4 T cell modularity scores for PSC-UC right colon and left colon T cells. Each dot represents one cell. **C |** Violin plots displaying CD8 T cell modularity scores for UC right colon and left colon T cells. Each dot represents one cell. **D |** Noninflamed left colon and right colon CD4 T cell modularity scores of UC. Each dot represents one cell. P value as determined by two-sided Mann-Whitney U test = $5.05x10^{-8}$.

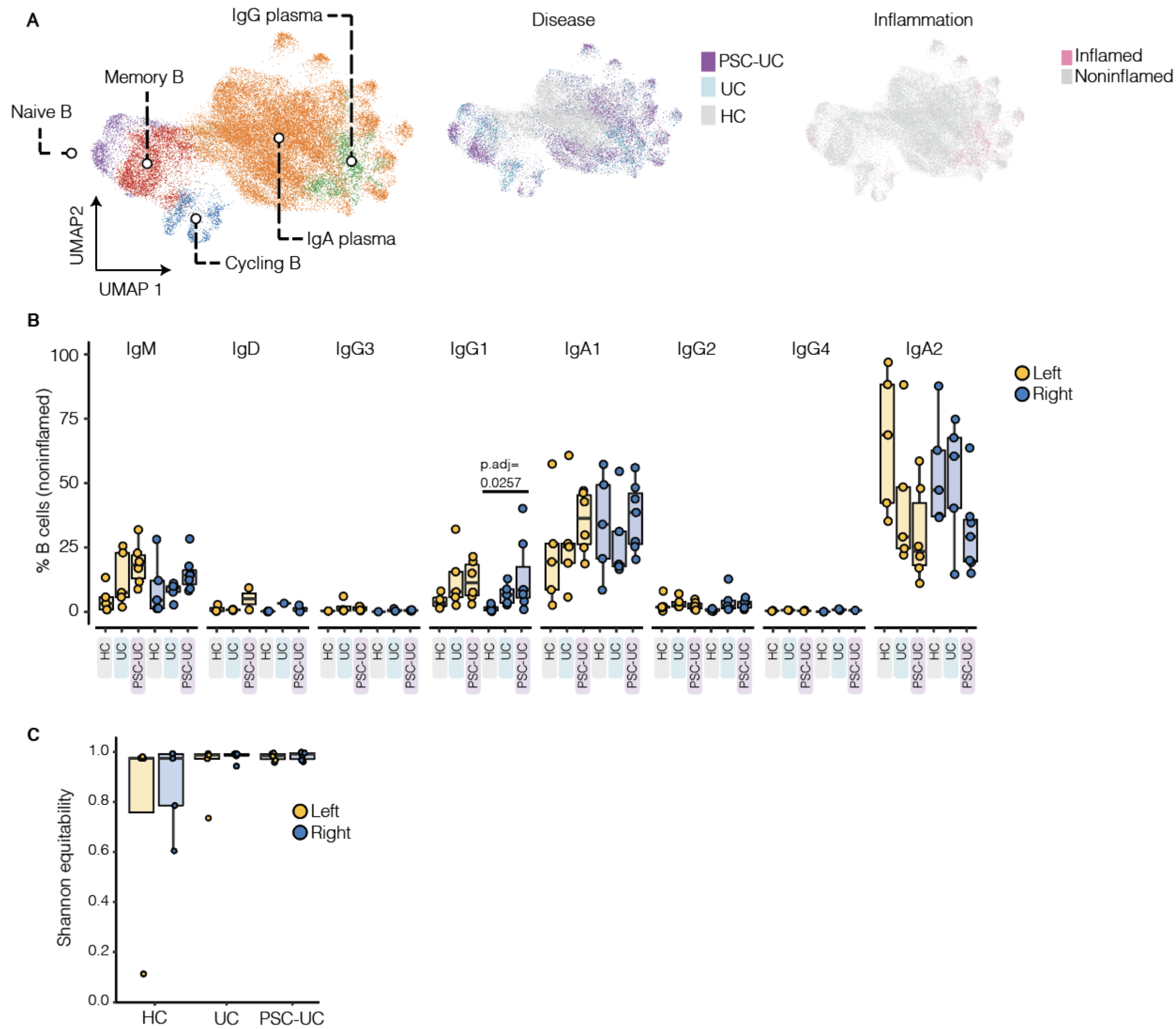

Supplementary Figure 8

A | UMAP of B lineage cells from the scRNA-seq dataset coloured by cell type, disease, and inflammation. **B |** Percent abundance of each isotype class within total functional B cell receptor (BCR) sequences from scRNA-sequencing data of healthy controls (HC) and UC and PSC-UC patients. Colour points represent pooled colonic biopsies from right (ascending or transverse colon) or left (descending colon or rectum) colon. Only sequences from noninflamed colon regions are included. Boxes show median and interquartile range and whiskers show minimum and maximum, excluding outliers. N = 5 for HC left, 5 for HC right, 5 for UC left, 5 for UC right, 6 for PSC-UC left, 6 for PSC-UC right. **C |** Shannon Equitability scores of BCR sequences separated by isotype of noninflamed colon regions of UC and PSC-UC patients. Colour points represent pooled colonic biopsies from right (ascending and transverse colon) or left (descending colon and rectum) colon. ANOVA (cohort + side + isotype) with Tukey HSD

post-test and multi hypothesis corrections showed no significance. Boxes show median and interquartile range and whiskers show minimum and maximum, excluding outliers. N = 4 for HC left, 5 for HC right, 5 for UC left, 5 for UC right, 6 for PSC-UC left, 6 for PSC-UC right.

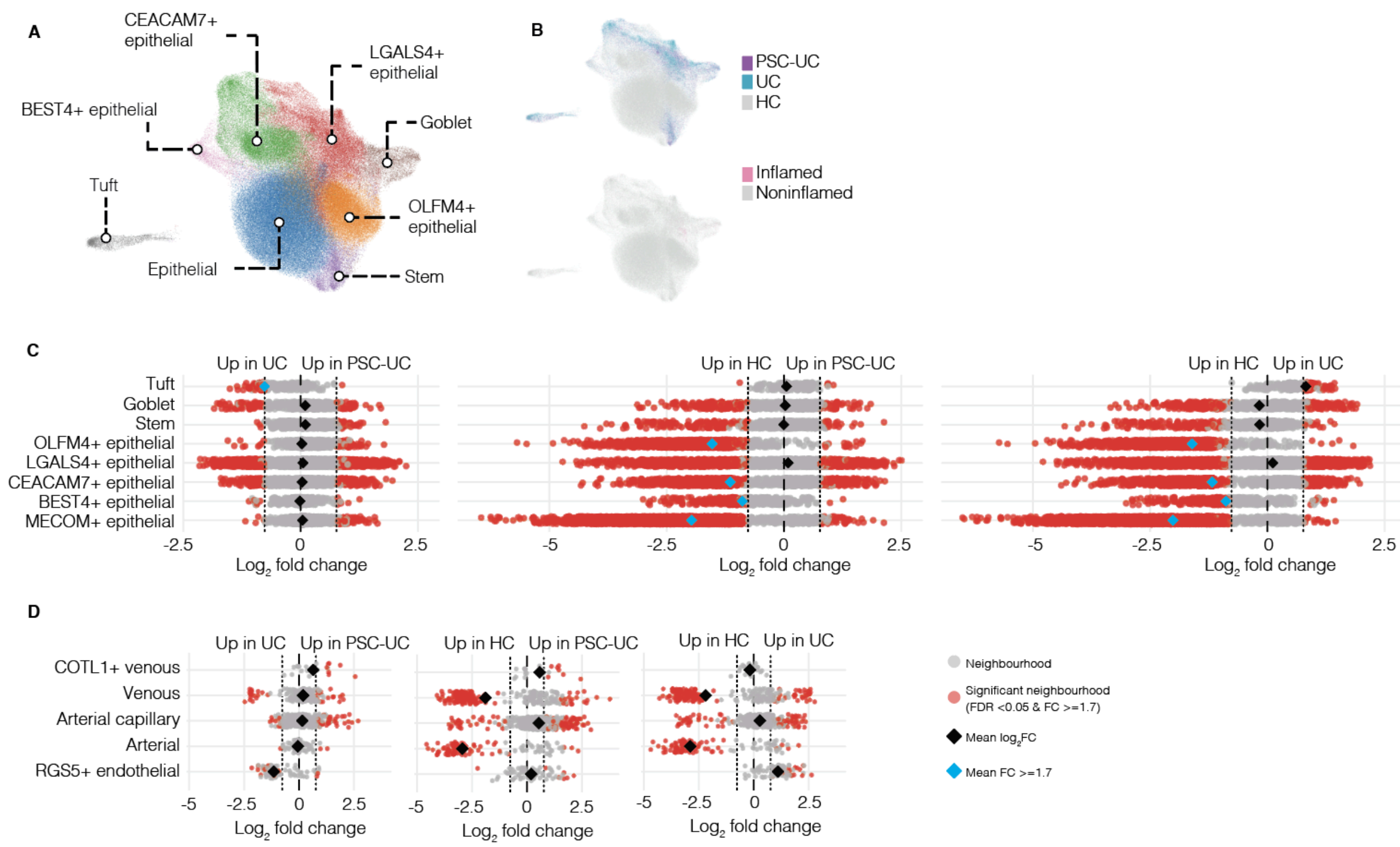

Supplementary Figure 9

A | UMAP of epithelial lineage cells from the scRNA-seq dataset coloured by annotation. **B |** UMAP of epithelial lineage cells from the scRNA-seq dataset coloured by disease and inflammation. **C |** Milo analysis showing differential epithelial subcluster abundance for UC vs. PSC-UC, PSC-UC vs. HC and UC vs. HC. Each dot represents a neighbourhood and significantly altered neighbourhoods are shown in red. **D |** Milo analysis showing differential endothelial cell subcluster abundance for UC vs. PSC-UC, PSC-UC vs. HC and UC vs. HC. Each dot represents a neighbourhood and significantly altered neighbourhoods are shown in red.

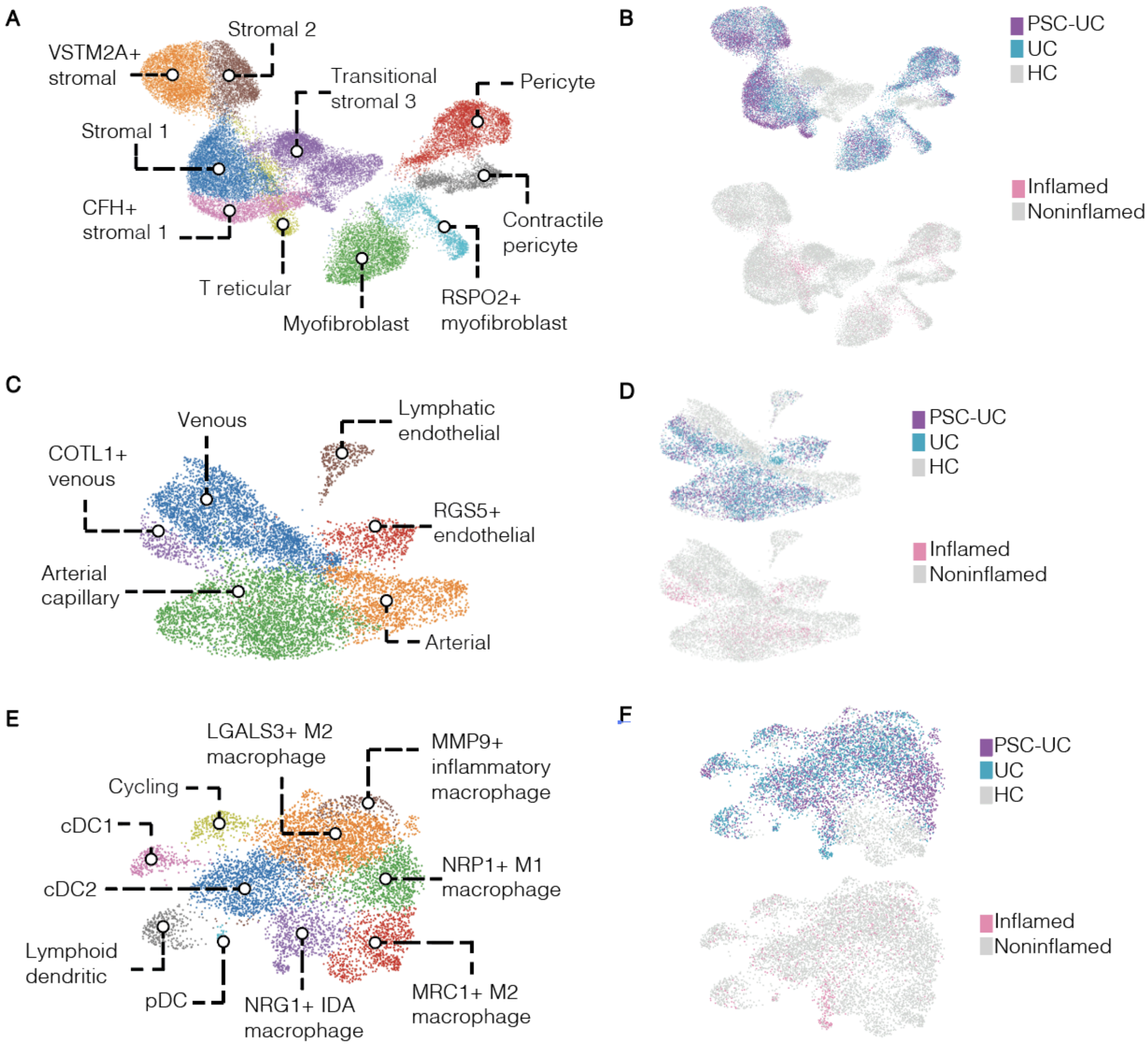

Supplementary Figure 10

A | UMAP of stromal lineage cells from the scRNA-seq dataset coloured by annotation. **B |** UMAP of stromal lineage cells from the scRNA-seq dataset coloured by disease and inflammation. **C |** UMAP of endothelial lineage cells from the scRNA-seq dataset coloured by annotation. **D |** UMAP of endothelial lineage cells from the scRNA-seq dataset coloured by disease and inflammation. **E |** UMAP of mononuclear phagocyte system cells from the scRNA-seq dataset coloured by annotation. IDA, inflammation-dependent alternative macrophage. **F |** UMAP of mononuclear phagocyte system cells from the scRNA-seq dataset coloured by disease and inflammation.

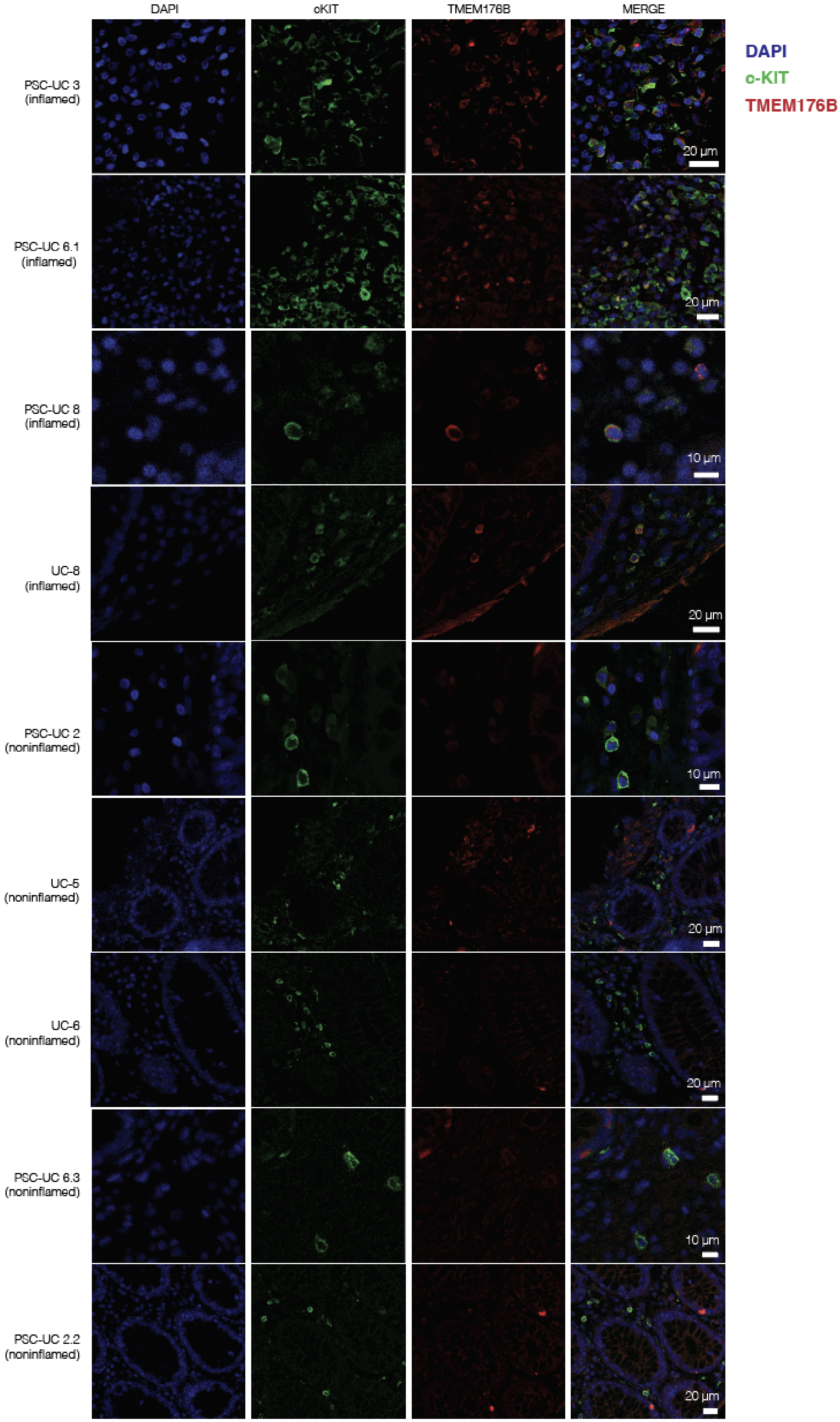

DAPI
cKIT
TMEM176B
MERGE
DAPI
c-KIT
TMEM176B
PSC-UC 3 (inflamed)
PSC-UC 6.1 (inflamed)
PSC-UC 8 (inflamed)
UC-8 (inflamed)
PSC-UC 2 (noninflamed)
UC-5 (noninflamed)
UC-6 (noninflamed)
PSC-UC 6.3 (noninflamed)
PSC-UC 2.2 (noninflamed)
20 µm
20 µm
10 µm
20 µm
10 µm
20 µm
20 µm
10 µm
20 µm

Supplementary Figure 11

Representative immunofluorescence images showing staining for TMEM176B+ inflammation-associated mast cells in inflamed and noninflamed donor colon samples.

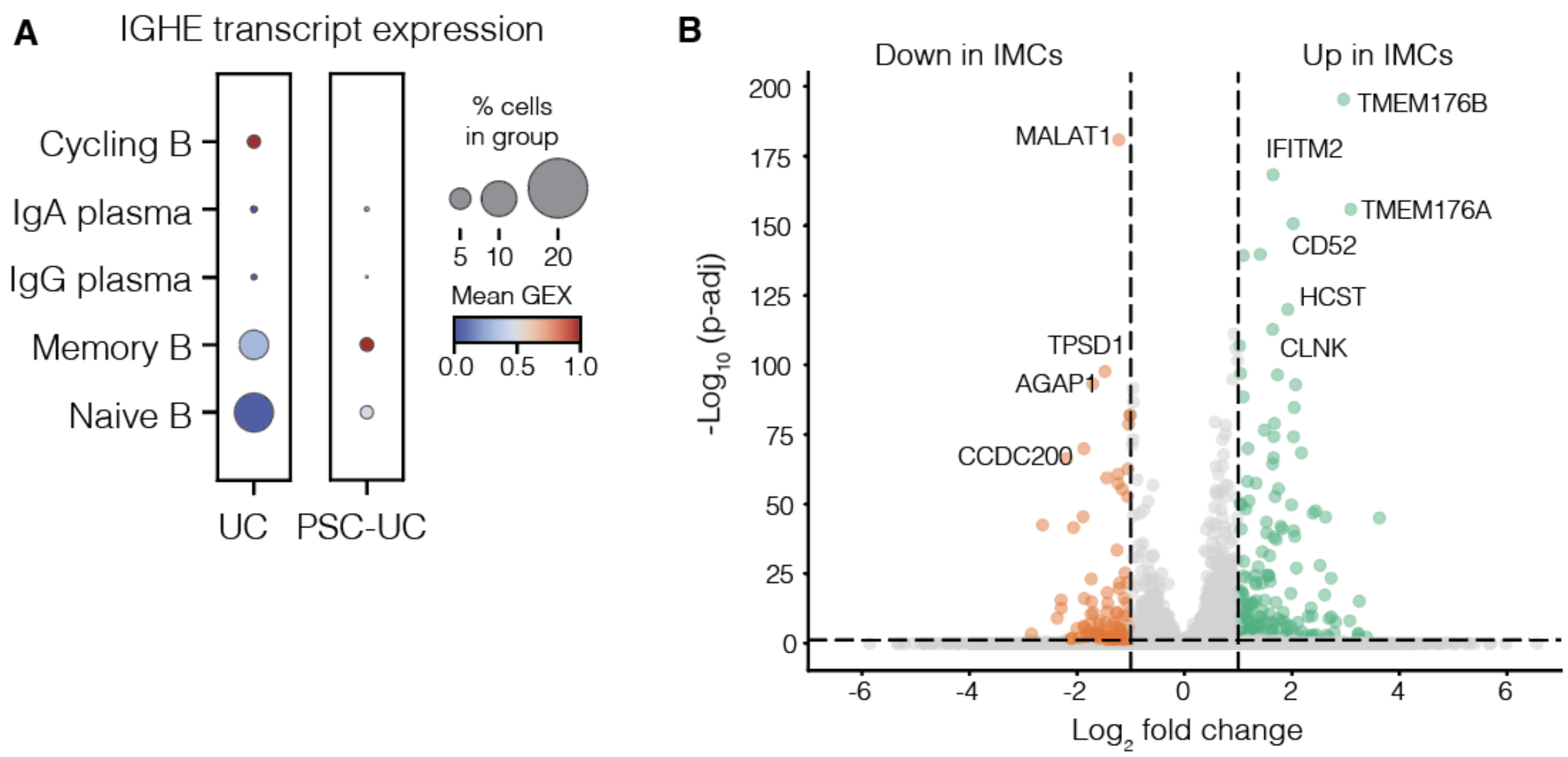

Supplementary Figure 12

A | Dot plot showing IGHE transcript expression in B lineage cells of the inflamed dataset. **B |** Volcano plot showing significantly upregulated and downregulated IMC genes from Supplementary Data 24 as determined by Scanpy's Wilcoxon sum test with Benjamini-Hochberg correction. Dotted lines at y = 0.05 and x = 1 and -1.

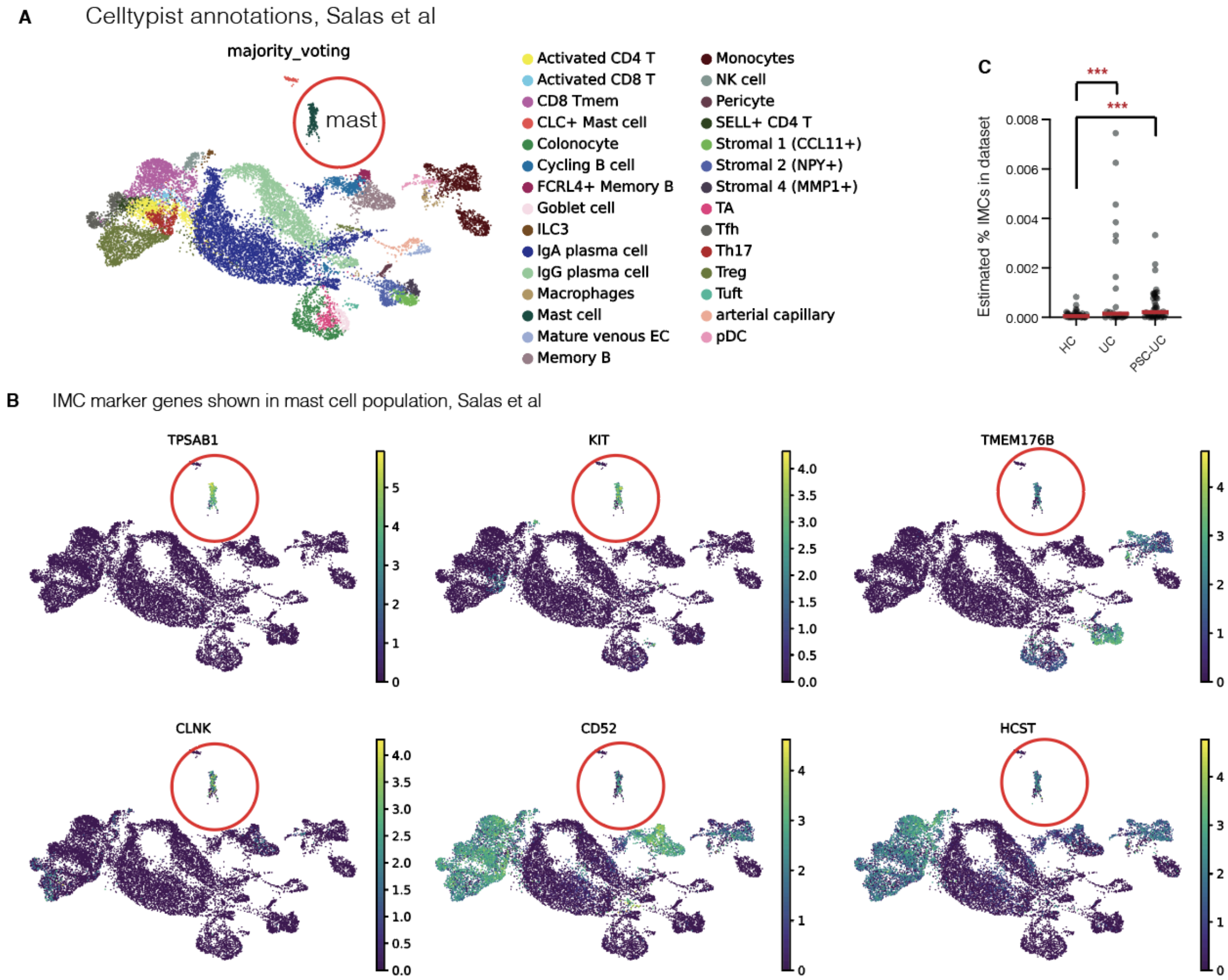

Supplementary Figure 13

A | UMAP of a published ulcerative colitis scRNA-seq dataset[23] coloured by Celltypist cell type annotation and **B |** IMC marker genes. **C |** Deconvolution of Quraishi et al.[7]'s PSC-UC, UC and healthy (HC) bulk RNA sequencing dataset using the IMC signature from our own scRNA-seq data. P values as determined by two-tailed unpaired T test = 0.0006 for HC vs. UC and 0.0004 for PSC-UC vs HC. N = 40 for HC, 24 for UC, and 40 for PSC-UC. No inflammation metadata was provided for this dataset.

Supplementary Table 1 - Primers

Table showing primers used in qPCR analyses.

Primer target	Source	Sequence	Final concentration
Rpl13a forward	KiCqStart SYBR green predesigned qPCR primers Sigma #KSPQ12012		1 μm
Rpl13a reverse			1 μm
Clnk forward			1 μm
Clnk reverse			1 μm
Tnf forward	Sigma aldrich	CCCTCACACTCAGATCATCTTCT	0.25 μm
Tnf reverse	Sigma aldrich	GCTACGACGTGGGCTACAG	0.25 μm
Il6 forward	Sigma aldrich	TAGTCCTTCCTACCCCAATTTCC	0.25 μm
Il6 reverse	Sigma aldrich	TTGGTCCTTAGCCACTCCTTC	0.25 μm
