# Supplementary material for: Primary sclerosing cholangitis displays distinct colonic mucosa topography yet a shared mast cell state with ulcerative colitis
Source: Nat Commun. 2026 Jul 7;17:10107. doi: 10.1038/s41467-026-75231-1 (PMC13597510; doi:10.1038/s41467-026-75231-1)
Supplement: Supplementary file 2 [file 41467_2026_75231_MOESM28_ESM.pdf]

nature portfolio

Corresponding author(s): *Double-anonymous peer review submissions: write DAPR and your manuscript number here instead of author names.*

Last updated by author(s): *YYYY-MM-DD*

Reporting Summary

Nature Portfolio wishes to improve the reproducibility of the work that we publish. This form provides structure for consistency and transparency in reporting. For further information on Nature Portfolio policies, see our Editorial Policies and the Editorial Policy Checklist.

Statistics

For all statistical analyses, confirm that the following items are present in the figure legend, table legend, main text, or Methods section.

n/a	Confirmed	
☐	☒	The exact sample size (*n*) for each experimental group/condition, given as a discrete number and unit of measurement
☐	☒	A statement on whether measurements were taken from distinct samples or whether the same sample was measured repeatedly
☐	☒	The statistical test(s) used AND whether they are one- or two-sided<br>*Only common tests should be described solely by name; describe more complex techniques in the Methods section.*
☐	☒	A description of all covariates tested
☐	☒	A description of any assumptions or corrections, such as tests of normality and adjustment for multiple comparisons
☐	☒	A full description of the statistical parameters including central tendency (e.g. means) or other basic estimates (e.g. regression coefficient) AND variation (e.g. standard deviation) or associated estimates of uncertainty (e.g. confidence intervals)
☐	☒	For null hypothesis testing, the test statistic (e.g. F, t, r) with confidence intervals, effect sizes, degrees of freedom and P value noted<br>*Give P values as exact values whenever suitable.*
☒	☐	For Bayesian analysis, information on the choice of priors and Markov chain Monte Carlo settings
☒	☐	For hierarchical and complex designs, identification of the appropriate level for tests and full reporting of outcomes
☒	☐	Estimates of effect sizes (e.g. Cohen's *d*, Pearson's *r*), indicating how they were calculated

Our web collection on statistics for biologists contains articles on many of the points above.

Software and code

Policy information about availability of computer code

Data collection	Single cell RNA sequencing: The Cell Ranger (v9.0) analysis pipeline using cellranger multi with default settings was used to generate counts files from gene expression and V(D)J library files and Demuxafy (v3.0) to perform SNP demultiplexing.<br>Single nucleotide polymorphism genotyping: Axiom Analysis Suite (5.3.0.45) from Thermo Fisher Scientific was used to generate VCF files.
Data analysis	Microbial amplicon sequencing data were processed using QIIME2 (v2025.7), DADA2, the SILVA v138.1 reference database, and analysed in R using phyloseq, vegan and DESeq2. Single-cell RNA sequencing analyses were performed in Python (v3.9.4) using Scanpy (v1.9.1), AnnData (v0.8.0), BBKNN, Scrublet, SCIRPY (v0.22.3), CellPhoneDB (v5.0.1), matplotlib (v3.5.2), NumPy (v1.21.6), pandas (v1.3.5), SciPy (v1.7.3) and seaborn (v0.11.2). Differential abundance testing was performed using MiloR. Cell Ranger (v9.0) was used for preprocessing of scRNA-seq and V(D)J libraries, Souporcell within the Demuxafy framework for SNP-based demultiplexing, and ArcasHLA (v0.5.0) together with Sinto (v0.10.1) for HLA analysis. Functional enrichment analysis was performed using Metascape. Bulk RNA-seq deconvolution was performed using Cellanneal (v1.1.0). B cell receptor analyses were performed using IgBLAST (v1.21), SCOPer (v1.3.0), R (v4.3.0), RStudio (2024.04.2+764) and tidyverse (v2.0.0). Spatial transcriptomic analyses were performed using the Stereo-seq Analysis Workflow (SAW), Stereopy (v1.5.1), TACCO (v0.4.0.post1) and Squidpy (v1.6.3). Figures from non-transcriptomic analyses were generated using GraphPad Prism (v10).

For manuscripts utilizing custom algorithms or software that are central to the research but not yet described in published literature, software must be made available to editors and reviewers. We strongly encourage code deposition in a community repository (e.g. GitHub). See the Nature Portfolio guidelines for submitting code & software for further information.

Data

Policy information about availability of data

All manuscripts must include a data availability statement. This statement should provide the following information, where applicable:

- Accession codes, unique identifiers, or web links for publicly available datasets
- A description of any restrictions on data availability
- For clinical datasets or third party data, please ensure that the statement adheres to our policy

The raw single-cell gene expression sequencing data generated in this study are available at ArrayExpress under accession numbers E-MTAB-14955 [https://www.ebi.ac.uk/biostudies/ArrayExpress/studies/E-MTAB-14955] and E-MAB-16401 [https://www.ebi.ac.uk/biostudies/ArrayExpress/studies/E-MTAB-16401]. The raw single-cell V(D)J sequencing data are available at ArrayExpress under accession numbers: E-MTAB-14901 [https://www.ebi.ac.uk/biostudies/ArrayExpress/studies/E-MTAB-14901], E-MTAB-14899 [https://www.ebi.ac.uk/biostudies/ArrayExpress/studies/E-MTAB-14899], E-MTAB-17148 [https://www.ebi.ac.uk/biostudies/ArrayExpress/studies/E-MTAB-17148], and E-MTAB-17213 [https://www.ebi.ac.uk/biostudies/ArrayExpress/studies/E-MTAB-17213]. Processed single-cell gene expression and V(D)J sequencing data are available at CELLxGENE at https://cellxgene.cziscience.com/collections/75e35754-bd21-4562-a4f8-39eaa4dfce36. Raw and processed STOmics spatial transcriptomics data are available at ArrayExpress under accession number E-MTAB-17162 [https://www.ebi.ac.uk/biostudies/ArrayExpress/studies/E-MTAB-17162?query=%20E-MTAB-17162]. Raw 16S ribosomal RNA sequencing data are available at ENA under accession number PRJEB86073 (ERP169448) [https://www.ebi.ac.uk/ena/browser/view/PRJEB86073] and processed genus-level counts are available in Supplementary Data 4 of this manuscript. All data are included in the Supplementary Information or available from the authors, as are unique reagents used in this Article. The raw numbers for charts and graphs are available in the Source Data file whenever possible. Previously published raw single-cell gene expression and V(D)J sequencing data were accessed from ArrayExpress under accession numbers E-MTAB-9543 [https://www.ebi.ac.uk/biostudies/ArrayExpress/studies/E-MTAB-9543?query=E-MTAB-9543] and E-MTAB-9532 [https://www.ebi.ac.uk/biostudies/ArrayExpress/studies/E-MTAB-9532?query=E-MTAB-9532]

Research involving human participants, their data, or biological material

Policy information about studies with human participants or human data. See also policy information about sex, gender (identity/presentation), and sexual orientation and race, ethnicity and racism.

Reporting on sex and gender	Findings apply to both male and female sex. UC patient cohort consists of 5 females and 6 males. PSC-UC patient cohort consists of 10 males and 1 female. Healthy control cohort consists of 5 females and 10 males. Study was designed to include both male and female tissue for each cohort. Sex was provided by treating doctors with consent from study participants or as reported for published data. Sex based analyses were not performed due to lack of statistical power, instead only disease-based analyses were performed.
Reporting on race, ethnicity, or other socially relevant groupings	Ethnicity of particpants is included in patient metadata as provided by their treating doctors. As a result of geographical location, most participants are of Australian ethnicity.
Population characteristics	All relevant patient treatment history is reported in Supplementary Data 2.
Recruitment	Suitable study participants were flagged by collaborating clinicians (Dr. Simon Ghaly and Dr. Andrew Kim) and consented to the Australian IBD Microbiome study or the Gastrointestinal inflammation multi-omics study. All patients were undergoing routine endoscopy procedures at St Vincent's Hospital in Sydney, producing geographical selection bias. This may affect microbiome data as noted in the discussion.
Ethics oversight	Use of human material in this study was conducted in accordance with relevant ethical regulations and approved by the South Eastern Sydney Local Health District Research Ethics Committee (Australian IBD Microbiome (AIM) study67; HREC ref no. 18/173) and the St Vincent's Hospital Human Research Ethics Committee (2024/ETH01125). All participants provided informed written consent prior to enrolment

Note that full information on the approval of the study protocol must also be provided in the manuscript.

Field-specific reporting

Please select the one below that is the best fit for your research. If you are not sure, read the appropriate sections before making your selection.

☒ Life sciences ☐ Behavioural & social sciences ☐ Ecological, evolutionary & environmental sciences

For a reference copy of the document with all sections, see nature.com/documents/nr-reporting-summary-flat.pdf

Life sciences study design

All studies must disclose on these points even when the disclosure is negative.

Sample size	Sample size were determined by availability of donors within the sampling time-frame. No statistical methods were used to calculate appropriate sample size. We followed standards in the field and Human Cell Atlas criteria.
Data exclusions	No exclusion was applied to the uploaded raw data in ArrayExpress. For the final count matrix, we excluded doublets and low quality cells based on pre-established criteria for single cells. This criteria is further summarised in the Methods section.
Replication	Single-cell RNA sequencing was carried at on gut tissue from 7 PSC-UC, 8 UC donors and 7 healthy control individuals. 16S amplicon sequencing was carried out on gut tissue from 8 PSC-UC, 11 UC patients and 10 healthy control individuals. All technical and biological

	replications of experiments were successful. All further in vitro experiments were performed at least 5 times.
Randomization	Sample collection was based on patient availability at time of sampling. Patients were allocated into experimental groups (HC, UC or PSC-UC) based on disease diagnosis determined previously by treating doctors.
Blinding	Sample collection, sample processing and sample analysis were all performed by Dr. Jacqueline Tearle and therefore blinding was not possible during analysis. However, all preprocessing and analysis was performed using established workflows that exclude disease state from consideration (e.g. unsupervised clustering) and only statistically significant findings were considered.

Reporting for specific materials, systems and methods

We require information from authors about some types of materials, experimental systems and methods used in many studies. Here, indicate whether each material, system or method listed is relevant to your study. If you are not sure if a list item applies to your research, read the appropriate section before selecting a response.

Materials & experimental systems

n/a	Involved in the study
☐	☒ Antibodies
☐	☒ Eukaryotic cell lines
☒	☐ Palaeontology and archaeology
☒	☐ Animals and other organisms
☐	☒ Clinical data
☒	☐ Dual use research of concern
☒	☐ Plants

Methods

n/a	Involved in the study
☒	☐ ChIP-seq
☒	☐ Flow cytometry
☒	☐ MRI-based neuroimaging

Antibodies

Antibodies used	TotalSeq C0251 (394661, BioLegend, 1:100), TotalSeq C0252 (394663, BioLegend, 1:100), DNP-IgE (Sigma D8406, SPE-7, 1:1000), anti-C-KIT (3308, Cell Signalling, Ab81, 1:2000), anti-TMEM176B (ab236860, Abcam, 1:100), goat anti-rabbit IgG AF594 (ab150080, Abcam, 1:400), goat anti-mouse IgG1 AF488(ab150113, Abcam, 1:400).
Validation	anti-C-KIT (3308, Cell Signalling, Ab81) validated with NCI-H526 cells as shown on website.<br>anti-TMEM176B (ab236860, Abcam) validated with L02 (human immortal liver cell line) cells as shown on website.

Eukaryotic cell lines

Policy information about cell lines and Sex and Gender in Research

Cell line source(s)	The Cl.MC/C57.1 cell line was generated from the BL6 mouse and provided by Professors John Hunt and Stephen Galli.
Authentication	Cell line authenticated as described in Tsai, M. Cl. MC/C57. 1 (C57) mouse mast cell line is of BALB/c origin and tumorigenic in BALB/c mice. FASEB J. 10, A1253 (1996).
Mycoplasma contamination	Cell lines were negative for mycoplasma contamination.
Commonly misidentified lines (See ICLAC register)	*Name any commonly misidentified cell lines used in the study and provide a rationale for their use.*

Clinical data

Policy information about clinical studies

All manuscripts should comply with the ICMJE guidelines for publication of clinical research and a completed CONSORT checklist must be included with all submissions.

Clinical trial registration	N/A
Study protocol	N/A
Data collection	N/A
Outcomes	N/A

Plants

Seed stocks	N/A
Novel plant genotypes	N/A
Authentication	N/A
