# Supplementary material for: Primary sclerosing cholangitis displays distinct colonic mucosa topography yet a shared mast cell state with ulcerative colitis
Source: Nat Commun. 2026 Jul 7;17:10107. doi: 10.1038/s41467-026-75231-1 (PMC13597510; doi:10.1038/s41467-026-75231-1)
Supplement: Supplementary file 3 [file 41467_2026_75231_MOESM29_ESM.pdf]

nature portfolio

Peer Review File

Primary sclerosing cholangitis colonic mucosa displays distinct topography yet a shared mast cell state with ulcerative colitis

Corresponding Author: Dr Kylie James

This file contains all reviewer reports in order by version, followed by all author rebuttals in order by version.

Version 0:

Reviewer comments:

Reviewer #1

(Remarks to the Author)
The authors are to be commended for their large body of work and several novel findings by analysing in depth biopsies from several parts of the large bowel in PSC/U and UC patients. The paper is well written and the narrative is clear, but after reading some questions and concerns remain.

Major:
In fact this manuscript contains 3 studies: one on the spatial microbiota composition and differences between PSC/UC and UC patients; one on the differences between cellular profile of PSC/UC versus UC; and one on the mast cell signature in UC. These are not really linked in the manuscript.

Why did the authors limit the sample size to only 6 patients? They state this as a limitation and this is obvious. It is acknowledged that some of the assays are very expensive, but the scope of the study and the numerous tests (I counted 53 original figures in the main text) warrants a design with sufficient power.

Also, several of the medications the patients were using are supposed to have a profound effect on white blood cells. Why did the authors not select patients without the use of these medications and without UDCA, which might alter bile acid cross-talk with the colonic mucosa ? Medication use here potentially constitutes a major confounder. It should not be too difficult to select patients either without medication or only on mesalamine when embarking on such a huge effort.

Spatial mucosa adherent microbiota differences can only be reliably assessed by colonoscopy without prior cleansing and by a protective biopsy technique in order to avoid cross-contamination in the working channel of the endoscope. This is much different than comparing biopsies from different sites collected at autopsy (ref 12).
Why did the authors not use metagenome sequencing, which yields much higher resolution enabling to compare the microbiota at strain level now that we know that metabolic output can differ per strain in various species.

Minor:
The statement in the Discussion on line 420 is rather bold and not clarified.

Reviewer #2

(Remarks to the Author)
The paper by Tearle et al. compares PSC-ulcerative colitis (UC) with common non-PSC UC using single cell mRNA and antigen receptor sequencing, 16S ribosomal RNA gene analysis and spatial transcriptomics in colonic biopsies. There is already conclusive clinical and laboratory evidence that both are related but separate diseases. The relationship of PSC and UC is also interesting because PSC appears to mitigate colitis and bacterial outer membrane vesicles from gut pathobionts trigger hepatic inflammation in PSC (Bedke et al. Gut 2024, Dorner et al. Gastroenterology 2024).
In the current report, in contrast to UC, PSC-UC patients display an enrichment of CD8 T, γδ T and natural killer cells and reduced microbial diversity in the right colon, even in the absence of active disease. During flare, there is an expansion of a

TMEM176B+ inflammatory mast cell state in both diseases.

The major problem with this paper is simply the small number of patients. Thirteen patients are listed in a somewhat “messy” Table (mixed UC and PSC-UC out of order) with and without inflammation. However, when broken down into subgroups according to localization and inflammation, the n goes down to 2-4 per group (see for example Fig. 1 F, G and I). Fig. 2E reports on 3 patients, as does Fig. 3C, 3E and 4C-F. The strict restriction to non-inflamed group comparisons, of course, is mandatory to avoid confusion with acute cellular infiltrates during relapse. As mentioned in the Table quoted above, for some reason all biopsies were taken in 2022 and none thereafter. The delta between UC and UC-PSC with respect to Th17 and activated CD8 positive cells is around 20% only (Fig. 3C) and, even if statistically significant, the biological relevance of such a small difference is unclear. Details of the statistics are not given but, to this reviewer, with so many (multiple) uncorrected comparisons (Bonferroni?) and n:3-4 any such calculation is questionable.

Thus, this report covers an interesting topic with cutting edge technology but, in my view, is much too preliminary. If and when supported by an acceptable “n” it might be reconsidered.

Version 1:

Reviewer comments:

Reviewer #2

(Remarks to the Author)
By expanding significantly the number of patients, the manuscript is much more solid. I have no further critical comments.

Reviewer #3

(Remarks to the Author)
The article by Tearle et al represents an enormous effort to characterize differences between PSC-UC, UC, and healthy colon, including a variety of parameters including spatial-omics, TCR repertoire, microbiome, and scRNAseq. Although descriptive, the study does identify interesting differences that need to be followed up in future studies. The data are very dense and can be hard to follow at times given the breadth of different methods used. As a result, the general readership of Nature Communications may find the article challenging to read.

Specific comments:

1. Are the samples studied all from subsets of the same overall group of 8, 11, and 10 PSC-UC, UC, and HC, respectively? If not, then this should be discussed, although it appears multiple assays were performed on the same cohort.

2. The authors show there is enrichment for different specific organisms in different colon locations, and between PSC-UC and UC in flare and remission. They argue that the UC microbiome is more conserved in UC than in PSC-UC, and they claim that this “may have a greater role in driving pathology”. This is interesting but speculative and not addressed in the experiments.

3. Single cell RNA-seq analysis was performed on ~320,000 cells across diseased and HC samples (5 of which were from prior datasets). The vast majority of cells came from uninflamed regions. There is an impressive volume of data in Figure 2 that would be valuable to mine further. Of note, color schemes can be challenging to discern, especially for people like this reviewer who has partial red/green color blindness. Much of the T cell subset discussion is a descriptive analysis of different known T cell subsets, where they are located in colon regions, etc. Similarly, the receptor-ligand pair analysis provides some potential interesting in silico interactions, but this would be much more interesting of spatial transcriptomics was also performed to demonstrate that the cells expressing receptor-ligand pairs were in fact spatially co-localized (eg Figure 2H). Similarly, Figures 2I-L are descriptive and don’t provide much useful information to the overall T cell story.

4. Supplementary data on B cells is largely negative or as expected, eg prominent expression of IgA at mucosal surfaces.

5. Figure 3 describes other cell types identified using scRNA-seq including stromal cells, colonocytes, tuft cells, stem cells, macrophages, dendritic cells and endothelial cell subsets. Again, this provides a wealth of data, but the figure largely provides lists of genes, what they are known to do, and then speculation as to what it means in the context of UC and PSC-SC.

6. Figure 4 focuses on inflamed mucosal cells which represent the minority of the >300,000 cells studied. Nevertheless, this is still a reasonably-sized dataset. An interesting cell population (TMEM176B+) is identified which they name Inflammation Associated Mast Cells (IAM). This cell population had some interesting transcripts associated with it, with a link to FcER signaling. They also show spatial transcriptomics from a single UC patient demonstrating enrichment of these mast cells, which were localized in close proximity to endothelial cells, which is not surprising.

7. Finally in Figure 5, they explore whether these TMEM176B+ mast cells might be important in colitis and may be pro-tumorigenic. They perform some in vitro experiments by stimulating a mast cell line through FcER and measuring TNF and IL6 by RT-PCR. They also block this using BAYK8644, but they don’t describe what this drug is or does. They show that the

drug leads to enhancement of FCER signaling and cytokine expression at the transcript level, and they suggest that this implicates TMEM176B+ as a negative regulator. They then make the speculative leap that TMEM176B+ cells could play a role in platelet activation which could promote tumorigenesis.

8. Summary of Discussion and Overall Manuscript. There is a huge amount of data in this paper. However, the work is almost completely descriptive, with almost no spatial localization (except for 1 small part of the study from a single UC patient focused on one cell type), and no functional data (except for the cell line experiment performed in Figure 5). There is no real link between the microbiome components and the different cell subsets. All of the conclusions are speculative. The datasets could be very valuable to the UC and PSC-UC fields, but there are no firm new discoveries here that will change how we diagnose, manage, or define patients with these complex disorders.

Reviewer #4

(Remarks to the Author)
I have reviewed the revised manuscript and the authors' responses to the initial concerns.
The revision is an improvement on the original submission in several respects.

The patient cohort has been substantially expanded — 7 PSC-UC and 8 UC patients for single-cell RNA sequencing, and 6 PSC-UC, 7 UC and 8 healthy controls for 16S rRNA gene sequencing — and this addresses the reviewer 1 principal concern regarding statistical power and the feasibility of subgroup comparisons.

The introduction now more clearly states the overarching aims of the study, and the transitions between the three analytical sections read more cohesively than in the original submission.

One issue, however, remains unresolved: medication as a confounder.
The authors note in the Discussion that diverse therapeutic histories in IBD patients can influence both cellular responses and microbiome composition. I agree, and this is precisely why I find it difficult to fully evaluate the manuscript without a clearer account of treatment distribution across the two groups. The rebuttal letter does not provide this, and the main text continues to defer the reader to Supplementary Tables 1 and 2 without summarising what those tables contain.
What I am missing is straightforward: which proportion of PSC-UC versus UC patients were on UDCA, biologics, immunomodulators or corticosteroids at the time of sampling, and whether these distributions differ between groups in ways that could plausibly confound the T cell or microbiome findings. I accept that restricting inclusion to untreated patients was not feasible. That is not my request. What I am asking for is a summary table of medication use in the main text, and at minimum a brief discussion of whether the key findings hold across patients with broadly comparable treatment backgrounds.
The cited precedents for including treated patients are noted, but precedent does not substitute for transparency in this specific dataset.
I remain supportive of publication contingent on this point being addressed.

Version 2:

Reviewer comments:

Reviewer #3

(Remarks to the Author)
The manuscript has been substantially improved. Although still largely descriptive, there is less speculation now in interpretation of results. The datasets themselves will be highly useful to the field as they and others mine the data over time.

Open Access This Peer Review File is licensed under a Creative Commons Attribution 4.0 International License, which permits use, sharing, adaptation, distribution and reproduction in any medium or format, as long as you give appropriate credit to the original author(s) and the source, provide a link to the Creative Commons license, and indicate if changes were made.
In cases where reviewers are anonymous, credit should be given to 'Anonymous Referee' and the source.
The images or other third party material in this Peer Review File are included in the article's Creative Commons license, unless indicated otherwise in a credit line to the material. If material is not included in the article's Creative Commons license and your intended use is not permitted by statutory regulation or exceeds the permitted use, you will need to obtain permission directly from the copyright holder.
To view a copy of this license, visit https://creativecommons.org/licenses/by/4.0/

Response to the editorial team and reviewers

We sincerely thank the editorial team and reviewers for considering our manuscript "The primary sclerosing cholangitis colonic mucosa displays distinct topography and a shared colitis-associated mast cell state". We appreciate the constructive feedback provided and have addressed each point raised by the reviewers below (our responses are in blue). In particular, we would like to highlight that we have now doubled our patient cohort and ensured each analysis is performed in a minimum of n=5 or with 5 independent biological repeats inline with the editor and reviews requests. We have streamlined our story and improved the connection between microbial, cellular and mast cell vignettes. We feel that the validity and message of the manuscript has been significantly improved through this process.

Reviewer #1 (PSC UC, IBD, liver disease) (Remarks to the Author):

The authors are to be commended for their large body of work and several novel findings by analysing in depth biopsies from several parts of the large bowel in PSC/U and UC patients. The paper is well written and the narrative is clear, but after reading some questions and concerns remain.

Major:
In fact this manuscript contains 3 studies: one on the spatial microbiota composition and differences between PSC/UC and UC patients; one on the differences between cellular profile of PSC/UC versus UC; and one on the mast cell signature in UC. These are not really linked in the manuscript.

Thank you for acknowledging the degree of work in this manuscript and the clarity of the narrative. To better link the sections of the revised manuscript, we have more clearly stated the overarching objective of this study in the introduction: "To comprehensively assess differences and similarities in PSC-UC and UC disease mechanisms,…"

And in introducing the major findings:

"reveals a more conserved microbiome between UC patients compared to HC and PSC-UC even in the absence of active inflammation. We describe disease-specific T cell composition with anatomical patterning in PSC-UC that mirrors colon regions most commonly affected by inflammation. We describe convergence of cell signatures during inflammation, with a TMEM176B+ mast cell state shared by PSC-UC and UC and evidence for the involvement of TMEM176B in directing function towards immune cell recruitment."

We have also edited the results text to more smoothly transition between sections.
E.g.,
"After deeply contrasting PSC-UC and UC noninflamed intestinal cell environments, we next explored how they compared in the context of active inflammation."

Why did the authors limit the sample size to only 6 patients? They state this as a limitation and this is obvious. It is acknowledged that some of the assays are very expensive, but the scope of the study and the numerous tests (I counted 53 original figures in the main text) warrants a design with sufficient power.

In line with Reviewer 1 and 2's request for increased statistical power, we have carried out additional single cell RNA sequencing and 16S ribosomal DNA sequencing on 3 independent PSC-UC patients, 4 independent UC patients and 2 healthy control individuals. We have also repeated all in vitro and immunofluorescent validation experiments such that n=5 biological repeats.

Also, several of the medications the patients were using are supposed to have a profound effect on white blood cells. Why did the authors not select patients without the use of these medications and without UDCA, which might alter bile acid cross-talk with the colonic mucosa ? Medication use here potentially constitutes a major confounder. It should not be too difficult to select patients either without medication or only on mesalamine when embarking on such a huge effort.

Selecting patients not on treatment was infeasible for this prospectively recruited dataset. This is especially true for UDCA, which is commonly prescribed treatment for PSC. Selecting PSC-UC patients only on mesalamine would require severely restricting the inclusion criteria for this relatively rare form of IBD, making this study infeasible. Inclusion of patients on UDCA (also referred to as ursodiol or URSO) is similarly documented in notable published studies of the PSC-UC microbiome and/or intestinal cell response:

Quraishi, et al. "A Pilot Integrative Analysis of Colonic Gene Expression, Gut Microbiota, and Immune Infiltration in Primary Sclerosing Cholangitis-Inflammatory Bowel Disease: Association of Disease With Bile Acid Pathways." J. Crohns. Colitis, 2020

Shaw, et al. "Antigen-driven colonic inflammation is associated with development of dysplasia in primary sclerosing cholangitis", Nat. Med., 2023

Ilott, et al., "Tissue-dependent transcriptional and bacterial associations in primary sclerosing cholangitis-associated inflammatory bowel disease", Wellcome Open Res, 2021

Spatial mucosa adherent microbiota differences can only be reliably assessed by colonoscopy without prior cleansing and by a protective biopsy technique in order to avoid cross-contamination in the working channel of the endoscope. This is much different than comparing biopsies from different sites collected at autopsy (ref 12).

It is infeasible to collect biopsies from the right colon without bowel prep, as fecal obstructions prevent reaching that side of the bowel. Protective biopsy techniques were used. To control for the effects of bowel prep on the microbiome, we have now included microbial sequencing from healthy control individuals collected during routine colonoscopies with bowel prep.

Why did the authors not use metagenome sequencing, which yields much higher resolution enabling to compare the microbiota at strain level now that we know that metabolic output can differ per strain in various species.

We agree that metagenomics achieves higher resolution and the possibility for functional readout of the microbiome. However, the massive difference in amount of human (typically greater than 99% of reads) and bacterial DNA poses significant challenges for metagenomic analysis from biopsies. In particular, there is low sensitivity to detect lowly abundant bacteria and, while methods exist to deplete human DNA, there is debate within the field regarding the effect this has on the resulting sequencing results (see references below). We feel the 16S analysis conducted in the current manuscript forms the basis for future targeted shotgun comparison of the mucosa-adherent bacteria between PSC-UC and UC.

References:

Marchukov, et al., "Benchmarking microbial DNA enrichment protocols from human intestinal biopsies", Frontiers in Genetics, 2023

Chen, et al., "High Sensitivity of Shotgun Metagenomic Sequencing in Colon Tissue Biopsy by Host DNA Depletion", Genomics, Proteomics & Bioinformatics, 2023

Minor:
The statement in the Discussion on line 420 is rather bold and not clarified.

We have revised the discussion and removed the line "Reduced diversity in the ascending colon could be due to the downstream effects of defective bile acid production and lipid metabolism in the small intestine of PSC-UC patients".

Reviewer #2 (IBD, Ab-based therapy) (Remarks to the Author):

The paper by Tearle et al. compares PSC-ulcerative colitis (UC) with common non-PSC UC using single cell mRNA and antigen receptor sequencing, 16S ribosomal RNA gene analysis and spatial transcriptomics in colonic biopsies. There is already conclusive clinical and laboratory evidence that both are related but separate diseases. The relationship of PSC and UC is also interesting because PSC appears to mitigate colitis and bacterial outer membrane vesicles from gut pathobionts trigger hepatic inflammation in PSC (Bedke et al. Gut 2024, Dorner et al. Gastroenterology 2024).
In the current report, in contrast to UC, PSC-UC patients display an enrichment of CD8 T, γδ T and natural killer cells and reduced microbial diversity in the right colon, even in the absence of active disease. During flare, there is an expansion of a TMEM176B+ inflammatory mast cell state in both diseases.

The major problem with this paper is simply the small number of patients. Thirteen patients are listed in a somewhat “messy” Table (mixed UC and PSC-UC out of order) with and without inflammation. However, when broken down into subgroups according to localization and inflammation, the n goes down to 2-4 per group (see for example Fig. 1 F, G and I). Fig. 2E reports on 3 patients, as does Fig. 3C, 3E and 4C-F. The strict restriction to non-inflamed group comparisons, of course, is mandatory to avoid confusion with acute cellular infiltrates during relapse. As mentioned in the Table quoted above, for some reason all biopsies were taken in 2022 and none thereafter. The delta between UC and UC-PSC with respect to Th17 and activated CD8 positive cells is around 20% only (Fig. 3C) and, even if statistically significant, the biological relevance of such a small difference is unclear. Details of the statistics are not given but, to this reviewer, with so many (multiple) uncorrected comparisons (Bonferroni?) and n:3-4 any such calculation is questionable.

Thus, this report covers an interesting topic with cutting edge technology but, in my view, is much too preliminary. If and when supported by an acceptable “n” it might be reconsidered.

We have substantially increased the sample sizes of our datasets and validation experiments in the revised manuscript. In particular, we have included the additional biological replicates:

<u>Single cell RNA sequencing:</u>
3 PSC-UC patients (all in remission)
4 UC patients (one with active inflammation of the descending colon)
2 control individuals with no reported intestinal pathology.

Total single-cell dataset: 7 PSC-UC (6 without inflammation, 1 with inflammation), 8 UC patients (5 without inflammation, 3 with inflammation) and 2 controls (plus published data), with 4 colonic regions/individual.

<u>16S rDNA sequencing:</u>
2 PSC-UC (overlapping with additional single-cell RNA sequencing patients)
3 UC (overlapping with additional single-cell RNA sequencing patients)
8 control individuals without intestinal pathology, biopsies taken during routine colonoscopy.

Total 16S rRNA gene sequencing dataset: 6 PSC-UC (1 with inflammation in all colon regions), 7 UC (3 with inflammation in one or two colon regions) and 8 controls.

Additionally, we have ensured all *in vitro* assays for the mast cell response and immunofluorescence stainings have 5 independent biological replicates.

The donor samples used for each assay are clearly documented in the revised Supplementary Table 2 with metadata for each patient in Supplementary Table 1.

All single-cell analyses have been repeated in the revised manuscript to incorporate the expanded dataset. To determine differential abundances of cell types between disease and healthy control

samples, we have adopted a statistical method specifically designed to handle the overlapping cell states and technical noise (including from batch effects) of single cell RNA sequencing data. This method, termed ‘Milo’, uses k-nearest neighbor (KNN) graphs to allocate cells to ‘neighborhoods’. Hypothesis testing among biological conditions is then performed in a multiple comparisons manner for each neighbourhood to identify differentially abundant cell states while controlling the FDR across the graph neighborhoods, therefore taking into account the spatial clustering of the cells and testing the abundance independently of the annotations. Subsequently, the neighborhoods are assigned to the cell types based on cell annotations. The abundance of a cell type is considered significant if the absolute value of the median fold change of its neighborhoods is greater than 1.7. Statistically significant differences between disease conditions and healthy controls are denoted by blue dots e.g., Figure R1 (Figure 2D of manuscript), shown below for your reference.

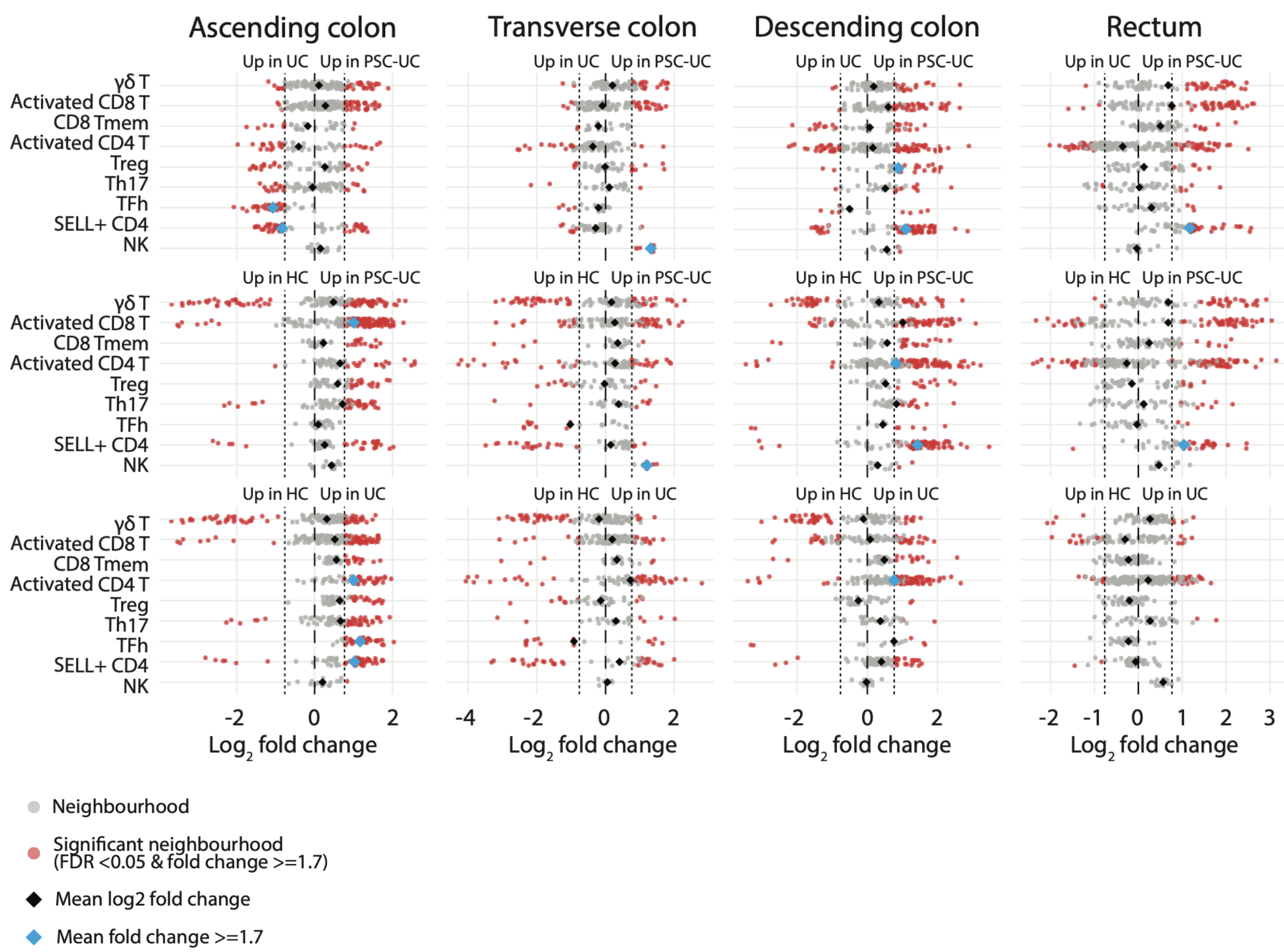

Figure R1 | Milo analysis showing differential T cell subcluster abundance of single-cell RNA sequencing analysis of the ascending colon, transverse colon, descending colon and rectum of PSC-UC (n=7) and UC (n=8) patients during remission, and HC (n=7). Top shows differential analysis for PSC-UC vs. UC, middle shows PSC-UC vs. HC, bottom shows UC vs. HC. Each dot represents a neighbourhood and significantly altered neighbourhoods are shown in red and comparisons with a median fold change of total neighborhoods >1.7 denoted in blue.

Response to the editorial team and reviewers

We sincerely thank the editorial team and reviewers for their most recent review of our manuscript "The primary sclerosing cholangitis colonic mucosa displays distinct topography and a shared colitis-associated mast cell state". We appreciate the constructive suggestions provided and have addressed each point raised by the reviewers below (our responses are in blue).

As requested by the editor, in particular, we have integrated receptor-ligand analyses with additional spatial transcriptomics to strengthen the conclusions about unique anatomical patterning of T cell signatures in PSC-UC. We have included these new results here as RF1.

RF1: Heatmaps of colocalising cell types with γδ T cells in spatial transcriptomics of 2 noninflamed biopsies of the descending colon of a PSC-UC patient. Arrows highlight cell types also predicted to interact with γδ T cells from ligand-receptor analysis of single cell RNA sequencing of biopsies from left colon of PSC-UC patients.

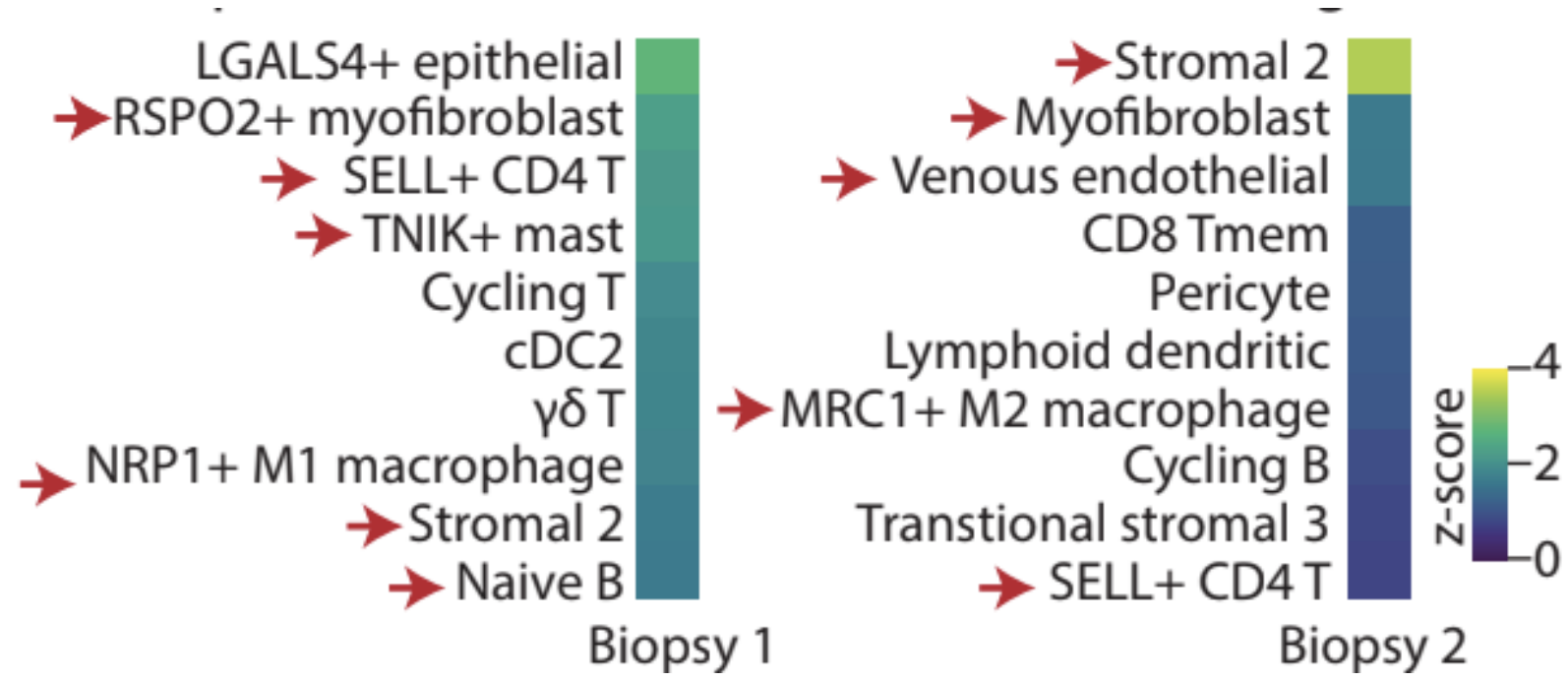

Additionally, we have reviewed all conclusions and have toned down/removed instances of speculation throughout the manuscript. We have amended the manuscript to better integrate the microbiome and single-cell sections by highlighting the associated distinct disease-specific anatomical patterning of cell and microbial signatures. For example:

"Having noted regionally distinct microbiome communities in PSC-UC and UC, we wondered whether the co-localising host cell landscapes also show regional specificity in these diseases. To this end, we performed single-cell gene expression analysis…"

Finally, as requested by Reviewer 4, we have included a summary table of medication use at the time of sampling for participants of this study in Figure 1 and below so the reader can better understand patient variability and potential confounders, and address this in the discussion.

RF2: Summary of fraction and percentage in parentheses of participants of this study on major drug classes at time of sampling.

Drug class	PSC-UC	UC	HC
UCDA	6/11 (55%)	0/11 (0%)	0/15 (0%)
Biologics	3/11 (27%)	6/11 (55%)	0/15 (0%)
Immunomodulators	2/11 (18%)	2/11 (18%)	0/15 (0%)
5-ASA	5/11 (46%)	3/11 (27%)	0/15 (0%)
Corticosteroids	0/11 (0%)	0/11 (0%)	0/15 (0%)

Reviewer #2 (Remarks to the Author):

By expanding significantly the number of patients, the manuscript is much more solid. I have no further critical comments.

Thank you for re-reviewing the manuscript and providing constructive comments.

Reviewer #3 (Remarks to the Author):

The article by Tearle et al represents an enormous effort to characterize differences between PSC-UC, UC, and healthy colon, including a variety of parameters including spatial-omics, TCR repertoire, microbiome, and scRNAseq. Although descriptive, the study does identify interesting differences that need to be followed up in future studies. The data are very dense and can be hard to follow at times given the breadth of different methods used. As a result, the general readership of Nature Communications may find the article challenging to read.

Specific comments:

1. Are the samples studied all from subsets of the same overall group of 8, 11, and 10 PSC-UC, UC, and HC, respectively? If not, then this should be discussed, although it appears multiple assays were performed on the same cohort.

As the reviewer suggests, there is overlap in the participants for the different assays. For clarity, we have now provided a summary of which participants overlap the different assays in Figure 1c and as RF3 for your convenience.

RF3: Summary table of participant identifiers used in major assays throughout the manuscript. PSC: Primary sclerosing chologitis, UC: ulcerative colitis, HC: healthy controls, *HC from a published single-cell RNA seq dataset.

scRNA-seq (GEX + VDJ)	16S rRNA seq	Immunofluorescence	STOmics
PSC-1, PSC-2.1, PSC-3, PSC-4, PSC-5, PSC-6.2, PSC-7, UC-1, UC-2, UC-3, UC-4, UC-7, UC-8, UC-9, UC-10, HC-1, HC-2, 411c*, 414c*, 417c*, 432c*, 440c*	PSC-1, PSC-2.1, PSC-3, PSC-4, PSC-6.2, PSC-7, UC-1, UC-2, UC-3, UC-4, UC-7, UC-8, UC-9, HC-3, HC-4, HC-5, HC-6, HC-7, HC-8, HC-9, HC-10	PSC-3, PSC-2.1, PSC-2.2, PSC-6.1, PSC-6.3, PSC-8, UC-5, UC-6, UC-8	PSC-8 UC-11

2. The authors show there is enrichment for different specific organisms in different colon locations, and between PSC-UC and UC in flare and remission. They argue that the UC microbiome is more conserved in UC than in PSC-UC, and they claim that this “may have a greater role in driving pathology”. This is interesting but speculative and not addressed in the experiments.

We have removed the suggestion that a more conserved microbiome in UC may suggest a greater role for the microbiome in driving pathology. We include the revised sentence here:

“These results highlight disease-specific mucosal-adherent microbiomes in remission and during flare, with a more conserved microbiome between UC patients, pointing to fundamentally different microenvironments in PSC-UC and UC.”

3. Single cell RNA-seq analysis was performed on ~320,000 cells across diseased and HC samples (5 of which were from prior datasets). The vast majority of cells came from uninflamed regions. There is an impressive volume of data in Figure 2 that would be valuable to mine further. Of note, color schemes can be challenging to discern, especially for people like this reviewer who has partial red/green color blindness. Much of the T cell subset discussion is a descriptive analysis of different known T cell subsets, where they are located in colon regions, etc. Similarly, the receptor-ligand pair analysis provides some potential interesting in silico interactions, but this would be much more interesting of spatial transcriptomics was also performed to demonstrate that the cells expressing receptor-ligand pairs were in fact spatially co-localized (eg Figure 2H). Similarly, Figures 2I-L are descriptive and don’t provide much useful information to the overall T cell story.

We appreciate the reviewers request to change the colour scheme to benefit readers with colour-blindness. We have made these changes and include an example here as RF4.

RF4: Percentage of γδ T and activated CD8+ T cells among total unconventional and conventional T cells within single-cell RNA sequencing data of right (pooled ascending and transverse colon) and left (pooled descending colon and rectum) of PSC-UC colon. *p=0.05, **p=0.01

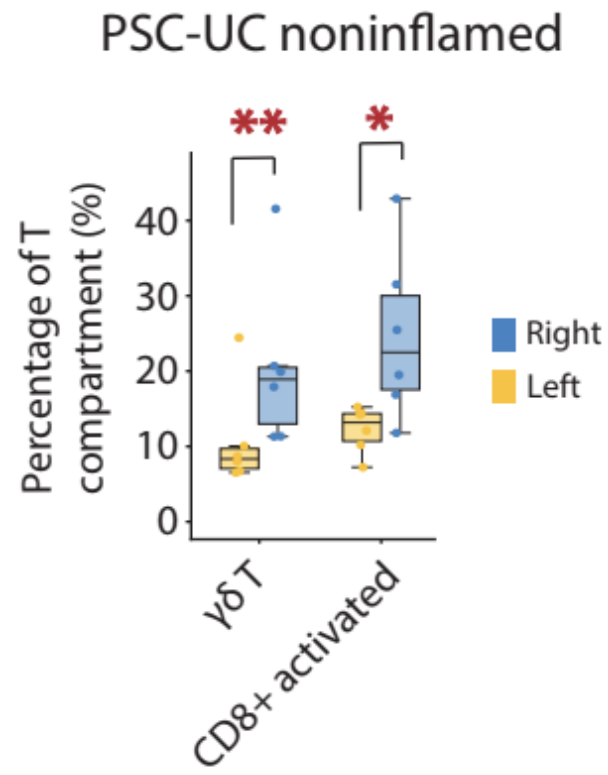

We also appreciated the reviewer's suggestion to support the ligand-receptor analysis with spatial transcriptomics and have included additional spatial transcriptomics of 2 biopsies from an uninflamed PSC-UC patient to support T cell co-localising with signaling partners (RF1). We agree that this additional data strengthens our conclusions about γδ T cell interactions with neighbouring cell types.

RF1: Heatmaps of colocalising cell types with γδ T cells in spatial transcriptomics of two noninflamed biopsies of the descending colon of a PSC-UC patient. Arrows highlight cell types also predicted to interact with γδ T cells from ligand-receptor analysis of single cell RNA sequencing of biopsies from left colon of PSC-UC patients.

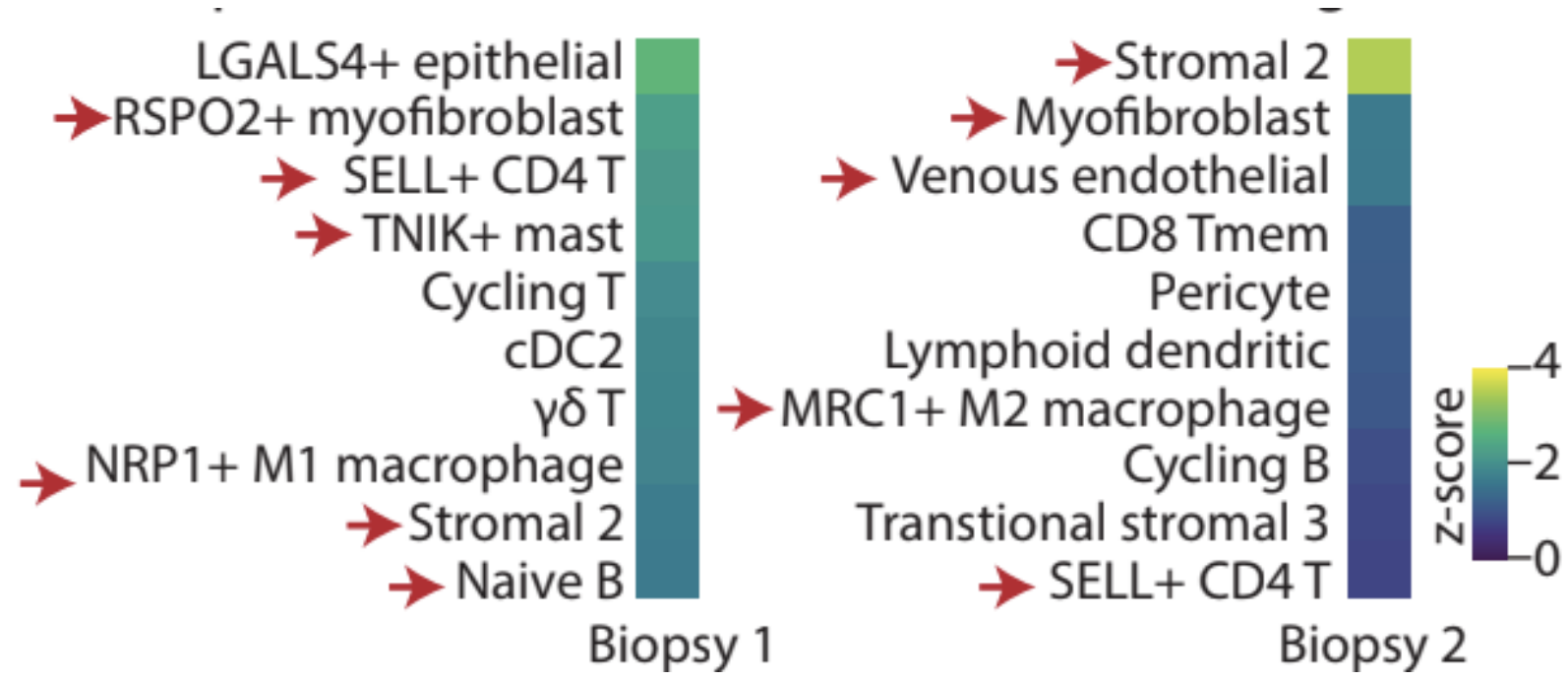

4. Supplementary data on B cells is largely negative or as expected, eg prominent expression of IgA at mucosal surfaces.

We have simplified the results section on B cells and highlighted that the results are as expected. Amended text is included here for your convenience.

"Analysis of B cell receptor sequences revealed an increase in IgG1 usage for both disease cohorts, as expected, and no difference in the clonal diversity (Shannon equitability) compared to HCs for either colon side."

5. Figure 3 describes other cell types identified using scRNA-seq including stromal cells, colonocytes, tuft cells, stem cells, macrophages, dendritic cells and endothelial cell subsets.

Again, this provides a wealth of data, but the figure largely provides lists of genes, what they are known to do, and then speculation as to what it means in the context of UC and PSC-SC.

We agree that the section '*Distinct non-immune cellular and transcriptional signatures of the remitting PSC-UC colonic mucosa*' is descriptive in nature and provides many interesting signatures to be verified/followed that are beyond the scope of this study.

6. Figure 4 focuses on inflamed mucosal cells which represent the minority of the >300,000 cells studied. Nevertheless, this is still a reasonably-sized dataset. An interesting cell population (TMEM176B+) is identified which they name Inflammation Associated Mast Cells (IAM). This cell population had some interesting transcripts associated with it, with a link to FcER signaling. They also show spatial transcriptomics from a single UC patient demonstrating enrichment of these mast cells, which were localized in close proximity to endothelial cells, which is not surprising.

We thank the reviewer for their positive assessment of our TMEM176B+ mast cell data. While we agree that the localisation of mast cells near endothelial cells is a known occurrence, our findings provide at least two key new insights, 1) unique inflammation-associated mast cell signature validated in independent published UC single-cell RNA seq and bulk RNA seq datasets, and 2) ligand-receptor interactions and gene expression evidence to support the functional consequence of these inflammation-associated mast cells near endothelial cells during IBD - in immune recruitment and vascular remodeling.

7. Finally in Figure 5, they explore whether these TMEM176B+ mast cells might be important in colitis and may be pro-tumorigenic. They perform some in vitro experiments by stimulating a mast cell line through FcER and measuring TNF and IL6 by RT-PCR. They also block this using BAYK8644, but they don't describe what this drug is or does. They show that the drug leads to enhancement of FCER signaling and cytokine expression at the transcript level, and they suggest that this implicates TMEM176B+ as a negative regulator. They then make the speculative leap that TMEM176B+ cells could play a role in platelet activation which could promote tumorigenesis.

We apologise for neglecting to describe BayK8644 and have now done this in text and below:

"We next assessed the role of TMEM176B in mast cell activation via the use of pharmacological inhibitor BayK8644, a L-type voltage-dependent calcium channel agonist shown to inhibit TMEM176B activity."

We have better supported our conclusion that TMEM176B+ cells could play a role in platelet activation and tumorigenesis by referring to our observation of their expression of *THBS1* and gene set enrichment analysis and making better reference to the link between IMCs and colorectal cancer. The concluding sentence of the IMC section here for your convenience:

"...possibly permitting orchestration of non-canonical mast cell functions, including platelet activation, as highlighted by gene set enrichment and expression of THBS1, which may in turn contribute to a possible pro-tumourigenic potential of IMC."

8. Summary of Discussion and Overall Manuscript. There is a huge amount of data in this paper. However, the work is almost completely descriptive, with almost no spatial localization (except for 1 small part of the study from a single UC patient focused on one cell type), and no functional data (except for the cell line experiment performed in Figure 5). There is no real link between the microbiome components and the different cell subsets. All of the conclusions are speculative. The datasets could be very valuable to the UC and PSC-UC fields, but there are no firm new discoveries here that will change how we diagnose, manage, or define patients with these complex disorders.

We thank the reviewer for their detailed assessment of our manuscript and for appreciating its value to the field. To improve the readability of the manuscript, we have better integrated the microbiome and single-cell sections through the associated distinct disease-specific anatomical patterning of cell and microbial signatures. For example:

"Having noted regionally distinct microbiome communities in PSC-UC and UC, we wondered whether the co-localising host cell landscapes also show regional specificity in these diseases. To this end, we performed single-cell gene expression analysis…"

In addition, we have toned down/removed instances of speculation throughout the manuscript.

Reviewer #4 (Remarks to the Author):

I have reviewed the revised manuscript and the authors' responses to the initial concerns.
The revision is an improvement on the original submission in several respects.

The patient cohort has been substantially expanded — 7 PSC-UC and 8 UC patients for single-cell RNA sequencing, and 6 PSC-UC, 7 UC and 8 healthy controls for 16S rRNA gene sequencing — and this addresses the reviewer 1 principal concern regarding statistical power and the feasibility of subgroup comparisons.

The introduction now more clearly states the overarching aims of the study, and the transitions between the three analytical sections read more cohesively than in the original submission.

One issue, however, remains unresolved: medication as a confounder.
The authors note in the Discussion that diverse therapeutic histories in IBD patients can influence both cellular responses and microbiome composition. I agree, and this is precisely why I find it difficult to fully evaluate the manuscript without a clearer account of treatment distribution across the two groups. The rebuttal letter does not provide this, and the main text continues to defer the reader to Supplementary Tables 1 and 2 without summarising what those tables contain.
What I am missing is straightforward: which proportion of PSC-UC versus UC patients were on UDCA, biologics, immunomodulators or corticosteroids at the time of sampling, and whether these distributions differ between groups in ways that could plausibly confound the T cell or microbiome findings. I accept that restricting inclusion to untreated patients was not feasible. That is not my

request. What I am asking for is a summary table of medication use in the main text, and at minimum a brief discussion of whether the key findings hold across patients with broadly comparable treatment backgrounds.

The cited precedents for including treated patients are noted, but precedent does not substitute for transparency in this specific dataset.

I remain supportive of publication contingent on this point being addressed.

We thank Reviewer 4 for their support of the manuscript and their assessment of the transparency of patient cohorts. We agree this is of paramount importance and have now included a summary table of the use of therapeutic classes at the time of sampling for our cohorts as Figure 1B and here as RF2. This shows comparable use by the disease groups except for UDCA. We have also summarised in text what Supplementary tables 1 and 2 show and expanded our discussion of treatment history and their possible effects on key findings in our manuscript. We include this discussion here for your convenience:

"Analysis of patient samples in this study presents notable limitations. Firstly, it was infeasible to limit this prospective study to treatment naive IBD patients, and their diverse therapeutic histories likely impact their cellular responses and microbiome compositions. Biologic, immunomodulator and 5-ASA use at the time of sampling are broadly comparable between disease cohorts in our study. Therefore, these specific therapeutic differences are unlikely to be the primary drivers of the difference in immune and microbial compositions reported between PSC-UC and UC. To further limit the effects of diverse therapeutic histories and strengthen the likelihood that reported signatures reflect fundamental, underlying disease biology, we performed inpatient comparisons of right vs. left when analysing anatomical differences between diseases and prioritised results consistent across all participants in a cohort. The primary exception to therapeutic comparability is UDCA, which was exclusively used by a subset of PSC-UC patients. UDCA, a therapeutic bile acid commonly used to treat chronic cholestatic liver diseases, has previously been demonstrated to alter the gut microbiome composition[60], and therefore presents a potential confounder for the observed PSC-UC microbiome signature."

RF2: Summary of fraction and percentage in parentheses of participants of this study on major drug classes at time of sampling.

Drug class	PSC-UC	UC	HC
UCDA	6/11 (55%)	0/11 (0%)	0/15 (0%)
Biologics	3/11 (27%)	6/11 (55%)	0/15 (0%)
Immunomodulators	2/11 (18%)	2/11 (18%)	0/15 (0%)
5-ASA	5/11 (46%)	3/11 (27%)	0/15 (0%)
Corticosteroids	0/11 (0%)	0/11 (0%)	0/15 (0%)
