# Supplementary material for: Primary sclerosing cholangitis displays distinct colonic mucosa topography yet a shared mast cell state with ulcerative colitis
Source: Nat Commun. 2026 Jul 7;17:10107. doi: 10.1038/s41467-026-75231-1 (PMC13597510; doi:10.1038/s41467-026-75231-1)
Supplement: Supplementary file 4 [file 41467_2026_75231_MOESM2_ESM.pdf]

Description of Additional Supplementary Files

File name: **Supplementary Data 1**
Description: Summaries of mean demographic, clinical and medication comparisons between disease cohorts in the study.

File name: **Supplementary Data 2**
Description: Full per-patient clinical metadata for all tissue samples used in the study.

File name: **Supplementary Data 3**
Description: A summary of inflammatory status, tissue processing and analytical assays applied to each tissue sample.

File name: **Supplementary Data 4**
Description: Genus-level raw counts for 16S RNA sequencing data from each biopsy and negative control samples. ACL, ascending colon; TCL, transverse colon; DCL, descending colon; REC, rectum.

File name: **Supplementary Data 5**
Description: PSC-UC vs. HC significantly abundant bacterial genera determined using DESeq2's negative binomial generalised linear model. Log2 fold changes are shown relative to the comparison group with Benjamini-Hochberg adjusted P values (padj).

File name: **Supplementary Data 6**
Description: PSC-UC vs. UC significantly abundant bacterial genera determined using DESeq2's negative binomial generalised linear model. Log2 fold changes are shown relative to the comparison group with Benjamini-Hochberg adjusted P values (padj).

File name: **Supplementary Data 7**
Description: UC vs. HC significantly abundant bacterial genera determined using DESeq2's negative binomial generalised linear model. Log2 fold changes are shown relative to the comparison group with Benjamini-Hochberg adjusted P values (padj).

File name: **Supplementary Data 8**
Description: Conserved differentially expressed genes for PSC-UC vs. UC samples across T cell subsets identified using Scanpy's Wilcoxon rank-sum test. Only genes with Benjamini–Hochberg adjusted P values < 0.05 were considered significant. To minimise batch effects, analyses were performed independently on the 2022 (fresh) and 2025 (frozen) datasets, and only genes that were significantly differentially expressed in both datasets with concordant direction of change were retained. Log fold changes, adjusted P values, direction of change and average absolute log fold changes are reported.

File name: **Supplementary Data 9**

Description: Conserved differentially expressed genes for PSC-UC vs. HC samples across T cell subsets identified using Scanpy's Wilcoxon rank-sum test. Only genes with Benjamini–Hochberg adjusted P values < 0.05 were considered significant. To minimise batch effects, analyses were performed independently on the 2022 (fresh) and 2025 (frozen) datasets, and only genes that were significantly differentially expressed in both datasets with concordant direction of change were retained. Log fold changes, adjusted P values, direction of change and average absolute log fold changes are reported.

File name: **Supplementary Data 10**
Description: Conserved differentially expressed genes for UC vs. HC samples across T cell subsets identified using Scanpy's Wilcoxon rank-sum test. Only genes with Benjamini–Hochberg adjusted P values < 0.05 were considered significant. To minimise batch effects, analyses were performed independently on the 2022 (fresh) and 2025 (frozen) datasets, and only genes that were significantly differentially expressed in both datasets with concordant direction of change were retained. Log fold changes, adjusted P values, direction of change and average absolute log fold changes are reported.

File name: **Supplementary Data 11**
Description: Predicted ligand-receptor interactions unique to right-sided PSC-UC γδ T cells. Significantly enriched ligand-receptor interactions involving γδ T cells identified using CellPhoneDB in the right colon of noninflamed PSC-UC patients. Only interactions unique to the right colon are shown. Interacting ligand-receptor pairs, interacting cell types and significant mean interaction scores are reported.

File name: **Supplementary Data 12**
Description: Predicted ligand-receptor interactions unique to left-sided PSC-UC γδ T cells. Significantly enriched ligand-receptor interactions involving γδ T cells identified using CellPhoneDB in the right colon of noninflamed PSC-UC patients. Only interactions unique to the left colon are shown. Interacting ligand-receptor pairs, interacting cell types and significant mean interaction scores are reported.

File name: **Supplementary Data 13**
Description: Conserved differentially expressed genes for UC vs. HC samples across epithelial cell subsets identified using Scanpy's Wilcoxon rank-sum test. Only genes with Benjamini–Hochberg adjusted P values < 0.05 were considered significant. To minimise batch effects, analyses were performed independently on the 2022 (fresh) and 2025 (frozen) datasets, and only genes that were significantly differentially expressed in both datasets with concordant direction of change were retained. Log fold changes, adjusted P values, direction of change and average absolute log fold changes are reported.

File name: **Supplementary Data 14**
Description: Conserved differentially expressed genes for PSC-UC vs. HC samples across epithelial cell subsets identified using Scanpy's Wilcoxon rank-sum test. Only genes with Benjamini–Hochberg adjusted P values < 0.05 were considered significant. To minimise batch

effects, analyses were performed independently on the 2022 (fresh) and 2025 (frozen) datasets, and only genes that were significantly differentially expressed in both datasets with concordant direction of change were retained. Log fold changes, adjusted P values, direction of change and average absolute log fold changes are reported.

File name: **Supplementary Data 15**
Description: Conserved differentially expressed genes for PSC-UC vs. UC samples across epithelial cell subsets identified using Scanpy's Wilcoxon rank-sum test. Only genes with Benjamini–Hochberg adjusted P values < 0.05 were considered significant. To minimise batch effects, analyses were performed independently on the 2022 (fresh) and 2025 (frozen) datasets, and only genes that were significantly differentially expressed in both datasets with concordant direction of change were retained. Log fold changes, adjusted P values, direction of change and average absolute log fold changes are reported.

File name: **Supplementary Data 16**
Description: Conserved differentially expressed genes for PSC-UC vs. UC samples across stromal cell subsets identified using Scanpy's Wilcoxon rank-sum test. Only genes with Benjamini–Hochberg adjusted P values < 0.05 were considered significant. To minimise batch effects, analyses were performed independently on the 2022 (fresh) and 2025 (frozen) datasets, and only genes that were significantly differentially expressed in both datasets with concordant direction of change were retained. Log fold changes, adjusted P values, direction of change and average absolute log fold changes are reported.

File name: **Supplementary Data 17**
Description: Conserved differentially expressed genes for PSC-UC vs. HC samples across stromal cell subsets identified using Scanpy's Wilcoxon rank-sum test. Only genes with Benjamini–Hochberg adjusted P values < 0.05 were considered significant. To minimise batch effects, analyses were performed independently on the 2022 (fresh) and 2025 (frozen) datasets, and only genes that were significantly differentially expressed in both datasets with concordant direction of change were retained. Log fold changes, adjusted P values, direction of change and average absolute log fold changes are reported.

File name: **Supplementary Data 18**
Description: Conserved differentially expressed genes for UC vs. HC samples across stromal cell subsets identified using Scanpy's Wilcoxon rank-sum test. Only genes with Benjamini–Hochberg adjusted P values < 0.05 were considered significant. To minimise batch effects, analyses were performed independently on the 2022 (fresh) and 2025 (frozen) datasets, and only genes that were significantly differentially expressed in both datasets with concordant direction of change were retained. Log fold changes, adjusted P values, direction of change and average absolute log fold changes are reported.

File name: **Supplementary Data 19**
Description: Conserved differentially expressed genes for PSC-UC vs. HC samples across endothelial cell subsets identified using Scanpy's Wilcoxon rank-sum test. Only genes with

Benjamini–Hochberg adjusted P values < 0.05 were considered significant. To minimise batch effects, analyses were performed independently on the 2022 (fresh) and 2025 (frozen) datasets, and only genes that were significantly differentially expressed in both datasets with concordant direction of change were retained. Log fold changes, adjusted P values, direction of change and average absolute log fold changes are reported.

File name: **Supplementary Data 20**
Description: Conserved differentially expressed genes for UC vs. HC samples across endothelial cell subsets identified using Scanpy's Wilcoxon rank-sum test. Only genes with Benjamini–Hochberg adjusted P values < 0.05 were considered significant. To minimise batch effects, analyses were performed independently on the 2022 (fresh) and 2025 (frozen) datasets, and only genes that were significantly differentially expressed in both datasets with concordant direction of change were retained. Log fold changes, adjusted P values, direction of change and average absolute log fold changes are reported.

File name: **Supplementary Data 21**
Description: Conserved differentially expressed genes for PSC-UC vs. UC samples across endothelial cell subsets identified using Scanpy's Wilcoxon rank-sum test. Only genes with Benjamini–Hochberg adjusted P values < 0.05 were considered significant. To minimise batch effects, analyses were performed independently on the 2022 (fresh) and 2025 (frozen) datasets, and only genes that were significantly differentially expressed in both datasets with concordant direction of change were retained. Log fold changes, adjusted P values, direction of change and average absolute log fold changes are reported.

File name: **Supplementary Data 22**
Description: Gene ontology (GO) biological process and reactome pathway enrichment analyses of genes upregulated in IMCs, performed using Metascape. Enriched terms were identified using the accumulative hypergeometric test with Benjamini–Hochberg false discovery rate correction. Log(P) values, enrichment factors, Z-scores, gene counts and log-transformed adjusted q-values are reported.

File name: **Supplementary Data 23**
Description: Significantly enriched ligand-receptor interactions involving γδ T cells identified using CellPhoneDB. Only interactions unique to IMCs (compared to other mast cells) are shown. Interacting ligand-receptor pairs, interacting cell types and significant mean interaction scores are reported.

File name: **Supplementary Data 24**
Description: Differentially expressed genes for mast cell subtypes vs. rest mast cells identified using Scanpy's Wilcoxon rank-sum test. Log fold change, p values and Benjamini-Hochberg adjusted p values are reported.

File name: **Supplementary Data 25**

Description: Sample pools, batch and multiplexing information for single cell RNA sequencing analyses.
